# Supplementary material for: Peptidyl nitroalkene inhibitors of main protease rationalized by computational and crystallographic investigations as antivirals against SARS-CoV-2
Source: Commun Chem. 2024 Jan 18;7:15. doi: 10.1038/s42004-024-01104-7 (PMC10796436; doi:10.1038/s42004-024-01104-7)
Supplement: Supplementary file 2 — Supplementary Information [file 42004_2024_1104_MOESM2_ESM.pdf]

Supplementary Material

Peptidyl Nitroalkene Inhibitors of Main Protease rationalized by Computational and Crystallographic Investigations as Antivirals against SARS-CoV-2

Francisco J. Medrano[1]*, Sergio de la Hoz-Rodríguez[2], Sergio Martí[3], Kemel Arafet[3], Tanja Schirmeister[4], Stefan J. Hammerschmidt[4], Christin Müller[5], Águeda González-Martínez[1], Elena Santillana[1], John Ziebuhr[5], Antonio Romero[1], Collin Zimmer[4], Annabelle Weldert[4], Robert Zimmermann[4], Alessio Lodola[6], Katarzyna Świderek[3], Vicent Moliner[3]*, Florenci V. González[2]*

[1]Centro de Investigaciones Biológicas Margarita Salas (CSIC), Ramiro de Maeztu 9, 28040 Madrid, Spain.

[2]Departament de Química Inorgànica i Orgànica, Universitat Jaume I, 12071-Castelló, Spain.
[3]Departament de Química Física i Analítica, Universitat Jaume I, 12071-Castelló, Spain.
[4]Institute of Pharmaceutical and BiomedicalSciences, Johannes Gutenberg-University Mainz, Staudinger Weg 5, 55128 Mainz, Germany.
[5]Institute of Medical Virology, Justus Liebig University Giessen, Schubertstrasse 81, 35392 Giessen, Germany.
[6]Dipartimento di Scienze degli Alimenti e del Farmaco, Università degli Studi di Parma, Italy.

INDEX

Experimental Methods	S2
Enzymatic assays	S4
X-ray studies	S10
Thermal denaturation	S16
Experimental procedures for the preparation of compounds	S17
Computational Methods	S22
Pharmacokinetic assays	S39
Supplementary References	S50

Supplementary Methods

Experimental Methods

Cloning of M^{pro} gene. The M^{pro} gene was cloned in two different vectors with a similar strategy. First, the sequence of the gene coding for M^{pro} (nsp5) SARS-CoV-2 was optimized for *Escherichia coli* expression and synthesized and cloned directly into pUCIDTKan vector by the company Integrated DNA Technologies (IDT, Leuven, Belgium) and named pUCIDTKan-M^{pro}. The M^{pro} gene was amplified from the vector pUCIDTKan-M^{pro} using the following primers: 5'-CGGGCGCCATATGTCTGCTGTTCTGCAGAGTG-3' (NdeI site) and 5'-CCGCTCGAGTTAATGGTGATGGTGATGG-3' (XhoI site) and cloned into the vector pET21a (Novagen) named pET21-M^{pro}. The cloned gene possess one M^{pro} autocleavage site SAVLQ↓SGFRK (arrow indicates the cleavage site) at the N-terminus, and at the C-terminus, the construct codes for the human rhinovirus 3C PreScission protease cleavage site (SGVTFQ↓GP) connected to a His6 tag. The authentic N-terminus is generated by M^{pro} autoprocessing during expression, whereas the authentic C-terminus is generated by the treatment with PreScission protease. Second, the M^{pro} gene was inserted into the pMal plasmid harboring the C-terminal hexahistidine-tagged sequence of SARS-CoV-2 Mpro named pMal-M^{pro} (Prof. John Ziebuhr, Justus Liebig University Gießen, Germany). The sequence contained the native nsp4/nsp5 M^{pro} cleavage site between MBP and Mpro as well as the native nsp5/nsp6 cleavage site between M^{pro} and the hexahistidine tag, thus enabling the purification of native M^{pro}.

Protein expression and purification. ***SARS-CoV-2 M^{pro}*.** The vector pET21-M^{pro} was transformed into *E. coli* Tuner (DE3) cells (Novagen, Merck, Madrid, Spain). These cells were grown in 2xYT medium supplemented with ampicillin (100 mg/L) at 37 °C. When the cells attained an OD_{600} of 0.6–0.8 the temperature was then dropped to 20 °C. When the temperature stabilized (approx., 15 min) the expression of the protein was induced by the addition of 0.1 mM isopropyl-β-D-thiogalactopyranoside (IPTG) and let to grow for an additional 16 h. The cells were harvested by centrifugation and resuspended in the lysis buffer containing 10 mM TRIS-HCl at pH 8.0 and 1% (v/v) TritonX-100. The cells were lysed by sonication and the insoluble fraction was removed by centrifugation at 45,000 × g for 1 hour; the supernatant was then loaded onto a HisTrap FF column (GE Healthcare). The column was washed extensively first with 10 mM TRIS-HCl buffer at pH 8.0 containing 5 mM imidazole, 0.5 M NaCl and 2 mM β-mercaptoethanol (β-ME) and subsequently with 10 mM TRIS-HCl buffer at pH 8.0 containing 50 mM imidazole, 0.5 M NaCl and 2 mM β-ME. The protein was eluted from the column in 10 mM TRIS-HCl buffer at pH 8 containing 300 mM imidazole, 0.5 M NaCl and 2 mM β-ME. Just after the elution of the protein, the concentration of the

β-ME was raised to 10 mM. The fractions containing the protease were then pooled, and PreScission protease containing a hexahistidine tag was added at a 500:1 molar ratio. The mixture was then dialyzed against a solution containing 10 mM TRIS-HCl at pH 8, 100 mM NaCl and 2 mM β-ME for 18 h at 4 °C. The PreScission-treated M^{pro} solution was applied to a HisTrap FF column to remove the PreScission protease, the C-terminal tag, and M^{pro} with uncleaved hexahistidine tag. The processed M^{pro} was collected in the flow-through and concentrated to 10 mg/mL. The generation of the proper N-terminal residue was confirmed by N-terminal sequencing by Edman degradation performed by the Protein Chemistry facility of the Centro de Investigaciones Biológicas (https://www.cib.csic.es/facilities/scientific-facilities/protein-chemistry). The expression of SARS-CoV-2 M^{pro} using the vector pMal-M^{pro} was performed exactly as described previously[1]. ***Human matriptase.*** Recombinant expression and purification were mainly performed as described previously[2]. The pQE30 plasmid, containing the human matriptase (membrane-type serine protease 1, MT-SP1, prostamin) was kindly provided by Prof. Torsten Steinmetzer (Philipps University Marburg, Germany). Since MT-SP1 is expressed as inclusion bodies, no leakage suppression was needed and, hence, the plasmid was transformed in *E. coli* BL21-Gold (DE3) (Agilent Technologies, Santa Clara, CA, USA) cells. After growing them in LB medium supplemented with ampicillin (100 mg/mL) to an OD_{600} of 0.6–0.8, overexpression was induced by addition of 1 mM IPTG over night (o.n.) at 20 °C. Cells were harvested by centrifugation, flash frozen in liquid N_2 and stored at −80 °C until further usage. For protein refolding and purification from inclusion bodies, cell pellets were resuspended in lysis buffer (50 mM TRIS−HCl pH 8.0, 300 mM NaCl, 10% (v/v) glycerol and 1 mM β-ME), supplemented with lysozyme and DNase and stirred for 1 h at room temperature (rt). After that, cells were further lysed by sonication (Sonoplus HD 2200; Bandelin, Berlin, Germany) and again centrifuged. The supernatant was discarded, and the pellet was washed with lysis buffer. Proteins were solubilized in a denaturing solubilization buffer (50 mM TRIS−HCl at pH 8.0, 6 M urea, 10% (v/v) glycerol and 1 mM β-ME) by stirring o.n. at rt. The suspension was again centrifuged to remove cell debris. The supernatant was subjected to IMAC on a HisTrap HP 5 ml column (Cytiva Europe GmbH, Freiburg im Breisgau. Germany), using IMAC buffer A (50 mM TRIS−HCl pH 8.0, 6 M urea, 20 mM imidazole and 1 mM β-ME) in a linear gradient with IMAC buffer B (50 mM TRIS−HCl at pH 8.0, 6 M urea, 250 mM imidazole and 1 mM β-ME). The fractions, containing eluted MT-SP1 were refolded by a 2-step dialysis over 12 h each at 4 °C in dialysis buffer A (50 mM TRIS−HCl at pH 9.0, 3 M urea and 1 mM β-ME) and anion exchange (IEX) buffer A (50 mM TRIS−HCl at pH 9.0, 1 mM β-ME) prior to IEX chromatography on a Resource Q 1 ml column (Cytiva Europe GmbH, Freiburg im Breisgau. Germany), using IEX buffer A in a linear gradient with IEX buffer B (50 mM TRIS−HCl at pH 9.0, 1 M NaCl and 1 mM β-ME). Eluted MT-SP1 was flash

frozen in liquid N_2 and stored at −80 °C. ***Zika Virus 2 NS2B$_{CF}$/NS3$_{pro}$.*** The bivalently expressed ZIKV protease was expressed and purified as described previously[3]. Briefly, the pETDUET vector containing bZiPro (purchased from Addgene) was transformed into competent *E. coli* BL21 Gold (DE3) cells (Agilent Technologies, Santa Clara, CA, USA) and grown in LB medium containing ampicillin at 37 °C until they attained an optical density (OD_{600}) of 0.8. Overexpression was induced o.n. by addition of 1 mM IPTG at 20 °C. After harvesting, cells were flash frozen in liquid N_2 and stored at −80 °C until protein purification. Herein, cell pellets were resuspended in lysis buffer (20 mM TRIS−HCl at pH 8.0, 300 mM NaCl, 20 mM imidazole, 0.1% (v/v) Triton X-100, RNase, DNase, lysozyme and 1 mM DTT) and lysed by sonication. After centrifugation, bZiPro from the cleared supernatant was purified by IMAC on a HisTrap HP 5 ml column with a step-gradient of washing buffer (20 mM TRIS−HCl at pH 8.0, 300 mM NaCl and 20 mM imidazole) and elution buffer (20 mM TRIS−HCl at pH 8.0, 300 mM NaCl and 250 mM imidazole). The eluted fractions, containing bZiPro were subjected to a gel filtration step (HiLoad 16/600 Superdex 75; GE Healthcare, Chicago, IL, USA) in SEC buffer (50 mM TRIS−HCl at pH 8.0 and 150 mM NaCl). Eluted bZiPro was flash frozen in liquid N_2 and stored at −80 °C. ***Cruzain.*** Cruzain (CRZ) was kindly provided by Dr. Avninder S. Bhambra (De Montfort University, Leicester, UK). ***Cathepsin L, Cathepsin B.*** Both cathepsin L (CatL) and cathepsin B (CatB) were purchased from Calbiochem (Merck Millipore, Burlington, Massachusetts). ***Rhodesain.*** Rhodesain (RhD) was recombinantly expressed and purified as reported previously[4].

Enzymatic assays. Proteolytic activity was determined by cleavage of fluorescence resonance energy transfer (FRET) peptide substrates. Fluorescence was measured using a Fluorolog-3 (Horiba Jobin Yvon, France) photon counting spectrofluorometer and a TECAN Infinite F2000 PRO plate reader (Agilent Technologies, Santa Clara, USA). The Fluorolog-3 spectrofluorometer was used to measure the activity of M^{pro} using the substrate Dabcyl-KTSAVLQ↓SGFRKME-(Edans)-Amid (Biosyntan, Berlin, Germany). They were carried out with excitation wavelength of 360 nm (4 nm band pass) and emission wavelength of 460 nm (8 nm band pass) using 5 x 10 mm cells at 25 °C, a protein concentration of 0.05 mg/mL (1.47 μM) and a substrate concentration of 5 μM with various concentrations of the inhibitors. The inhibitor was added into the M^{pro} solution in the reaction buffer, mixing and allowing the mixture to equilibrate for 10 sec, and then initiated by adding the substrate solution. Compounds in Table 1 were diluted in DMSO (**FGA145, FGA146** and **FGA147**), N,N-dimethylformamide (**FGA86** and **FGA177**) and ethanol (**FGA159**). Due to the deleterious effect of the solvents on the activity of M^{pro} and for consistency of the data, the concentration of solvent was kept constant at 1% (v/v) in all experiments. The fluorescence time course of the reaction mixture

was recorded continuously for 2 min in 10 mM TRIS-HCl buffer at pH 8.0 containing 0.1 M NaCl and 2 mM β-ME. The slope of the curve of fluorescence intensity with time quantitatively reflects the activity of the enzyme. The proteolytic reaction initial velocity in the presence or absence of the inhibitors was determined by linear regression using the data points from the first 10 sec of the kinetic progress curves. The IC_{50} was calculated by adjusting a sigmoidal curve to the initial velocities plotted against the inhibitor concentration with the program Origin2018 (https://www.originlab.com). All measurements were made in triplicate. The K_i values were calculated using Eq. 2. For measurements using the TECAN Infinite F200 PRO plate reader each well contained 200 µL, composed of 180 µL buffer, 5 µL enzyme in buffer, 10 µL inhibitor in DMSO or ethanol, and 5 µL substrate in DMSO (measuring conditions for all the proteases are summarized in Table S1). The amount of solvent in these experiments was 7.5%. The reaction was monitored for 10 min, fluorescence readout was performed in 30 s intervals. All measurements were made in triplicate. IC_{50} values were calculated using GRAFIT (Version 6.0.12; Erithacus Software Limited, East Grinstead, West Sussex, UK) by fitting the remaining enzymatic activity to the four-parameter IC_{50} equation (Eq. 1):[5,6]

$$Y = \frac{Y_{max} - Y_{min}}{1 + \left(\frac{[I]}{IC_{50}}\right)^s} \qquad \text{(Eq. 1)}$$

with Y as the substrate hydrolysis rate obtained as fluorescence increase over time (ΔF/min), Y_{max} as maximum value of the dose–response curve, measured at inhibitor concentrations of [I] = 0 µM, Y_{min} as the minimum value of the dose–response curve, obtained at high inhibitor concentrations, and s as the Hill coefficient. The K_i value was calculated using Eq. 2:[6]

$$K_i = \frac{IC_{50}}{1 + \frac{[S]}{K_m}} \qquad \text{(Eq. 2)}$$

with [S] being the used substrate concentration and K_m as the substrate concentration reaching half maximal hydrolysis activity (determined in a separate experiment).

FGA146, the indole harboring compound, was the only one revealing a strong fluorescence in this assay at higher concentrations. To rule out that bleaching of this fluorescence interferes with our readout by overlaying the fluorescence increase caused by the enzymatic substrate cleavage, control measurements were performed. Therefore, the assay was repeated without addition of substrate, hence, the negative slope due to bleaching of **FGA146** was determined. The relative activity values were then corrected by the negative slope of each inhibitor concentration (Figure S1).

Cell-based antiviral activity and cytotoxicity assays. Huh-7 cells that overexpress human angiotensin-converting enzyme 2 (ACE2) (Huh-7-ACE2; kindly provided by Friedemann Weber (Institute of Virology, Justus Liebig University Giessen)) were grown in Dulbecco's modified Eagle's medium (DMEM) supplemented with 10% fetal bovine serum (FBS) and antibiotics (100 U/mL penicillin and 100 µg/mL streptomycin) at 37 °C in an atmosphere containing 5% CO_2. The SARS-CoV-2 isolate Munich 929[7] was kindly provided by Christian Drosten (Institute of Virology, Charité-Universitätsmedizin, Berlin). Cytotoxic concentrations 50% (CC_{50}) of the compounds used in antiviral activity assays were determined using MTT assays as described previously[8]. To determine effective concentrations 50% (EC_{50}) of the respective compounds, Huh-7-ACE2 cells were inoculated with SARS-CoV-2 at a multiplicity of infection (MOI) of 0.1 plaque-forming units (pfu) per cell. After incubation for 1 h at 33 °C, the virus inoculum was replaced with fresh cell culture medium containing the test compounds at the indicated concentration. After 23 h at 33 °C, the cell culture supernatants were collected and virus titers were determined by virus plaque assay as described previously[8].

Crystallization data collection and structure determination. Crystallization trials were performed at 295 K using the sitting-drop vapor-diffusion method with commercial screening solutions including JBScreen Classic and Wizard I–IV (Jena Bioscience, Jena, Germany) in 96-well sitting-drop plates (Swissci MRC; Molecular Dimensions, Suffolk, England). Drops were set up by mixing equal volumes (0.2 µL) of protein-containing solution (8 mg/mL) and reservoir solution using a Cartesian Honeybee System (Genomic Solutions, Irvine, USA) nano-dispenser robot and equilibrated against 50 µL reservoir solution. Both crystals of the apo form of the protein were obtained in 0.1 M sodium HEPES at pH 7.0 containing 22% PEG 4000 and 3% DMSO. The complex with compound **FGA146** gave single well-diffracting crystals that were obtained in 0.1 M Bis-TRIS at pH 6.5 containing 18% PEG 3350, and the complex with compound **FGA147** gave single well-diffracting crystals that were obtained in 0.1 M TRIS-HCl at pH 8.5 containing 20% PEG 2000 MME and 10 mM $NiCl_2$.

For data collection, crystals were cryo-protected with a cryo-solution containing the reservoir supplemented with 30 % (v/v) glycerol and flash-cooled in liquid nitrogen. X-Ray data collection experiments were performed at the ALBA Synchrotron (Cerdanyola del Vallès, Spain) BL13 XALOC beamline, and at the ESRF Synchrotron (Grenoble, France) ID30B beamline. Data were indexed and integrated, scaled and merged using XDS[9]. The structures were solved by molecular replacement using the previously reported SARS-CoV2 M^{pro} structure (PDB: 7K3T) with Molrep[10]. The initial model was first refined using Refmac5[11] and alternating manual building with Coot[12]. MolRep and

Refmac5 are part of the CCP4 suite[13] The final model was obtained by repetitive cycles of refinement; solvent molecules were added automatically and inspected visually for chemically plausible positions. The inhibitor molecule was added manually. The stereochemical quality of the model was assessed with MolProbity[14]. The structural figures were generated using the Pymol program (http://www.pymol.org). Data processing and refinement statistics are listed in Table S2.

Circular dichroism. Circular dichroism measurements were carried out on a JASCO J-720 (Jasco, Tokyo, Japan) spectropolarimeter equipped with a Peltier type temperature controller and a thermostatized cuvette cell linked to a thermostatic bath. Spectra were recorded in 0.1 cm path length quartz cells with a response time of 4 sec and a band width of 2 nm. The protein concentration used was 0.15 mg/mL in 20 mM Tris-HCl buffer at pH 7.5 and 100 mM NaCl. The observed ellipticities were converted into the molar ellipticities [θ] based on a mean molecular mass per residue of 110.45 Da. Thermal denaturation experiments were performed by increasing the temperature from 20 to 80 °C at 30 °C/hour. Tm represents the temperature at the midpoint of the unfolding transition. The CD signal was followed at 230 nm and the concentration of organic solvent was kept constant at 2.5%. Two concentrations of each compound were used (25 and 100 μM).

Table 1. Parameters for enzyme inhibition assays.

Enzyme	Buffer	[Enzyme]	Substrate	[Substrate]	Ex./Em. (nm)
M^{pro}	20 mM TRIS−HCl pH 7.5, 100 mM NaCl, 2 mM β-ME	1.48 μM	Dabcyl-KTSAVLQSGFRKME-(Edans)-Amid[a]	5 μM (K_m: 9.12 μM)	360/460
M^{pro} [1]	20 mM TRIS−HCl pH 7.5, 200 mM NaCl, 0.1 mM EDTA, 1 mM DTT	250 nM	Dabcyl-KTSAVLQSGFRKME-Edans[b]	5 μM (K_m: 34 μM)	355/493
MT-SP1	50 mM TRIS−HCl pH 8.0, 150 mM NaCl, 5 mM $CaCl_2$, 0.01% (v/v) T_{x-100}	1.25 nM	Boc-LRR-AMC[c]	85 μM (K_m: 36 μM)	380/460
bZiPro[15,16]	50 mM TRIS pH 9.0, 20% (v/v) Glycerol, 1 mM Chaps	2 nM	Phac-LKKR-AMC[d]	10 μM (K_m: 6.5 μM)	380/460
RhD[17]	50 mM Na-acetate pH 5.5, 5 mM EDTA, 200 mM NaCl, 0.005% (m/v) Brij 35	0.5 nM	Cbz-FR-AMC[c]	10 μM (K_m: 0.62 μM)[18]	380/460
CRZ	50 mM Na-acetate pH 5.5, 5 mM EDTA, 200 mM NaCl, 0.005% (m/v) Brij 35	0.5 nM	Cbz-FR-AMC[c]	5 μM (K_m: 1.5 μM)	380/460
CatL[19]	50 mM TRIS−HCl pH 6.5, 5 mM EDTA, 200 mM	~50 ng/mL	Cbz-FR-AMC[c]	5 μM	380/460

	NaCl, 0.005% (m/v) Brij 35			(K_m: 6.5 μM)[20]	
CatB[19]	50 mM TRIS−HCl pH 6.5, 5 mM EDTA, 200 mM NaCl, 0.005% (m/v) Brij 35	~60 pg/mL	Cbz-FR-AMC[c]	80 μM (K_m: 150 μM)[20]	380/460
[a]purchased from Biosyntan GmbH (Berlin, Germany). [b]purchased from Genescript (New Jersey, USA). [c]purchased from Bachem (Bubendorf, Switzerland). [d]provided by Prof. Torsten Steinmetzer (Institute of Pharmaceutical Chemistry, Philipps University, Marburg, Germany).					

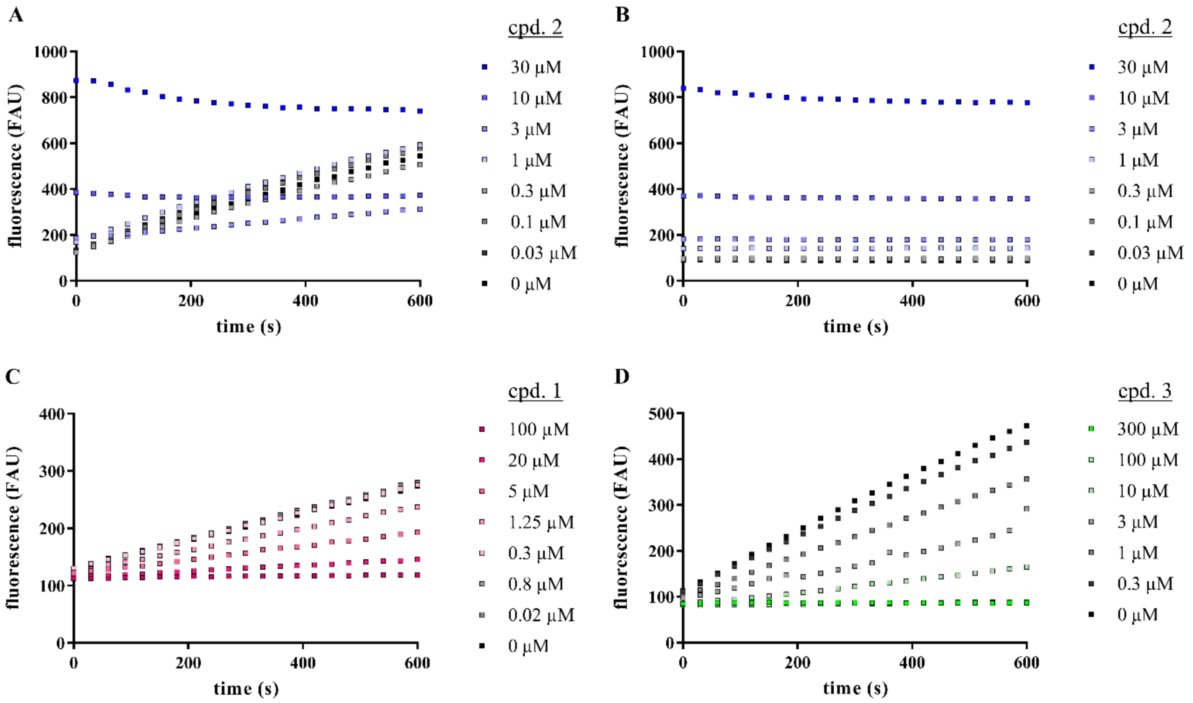

Figure 1. Exemplarily enzyme activity curves for the IC_{50} determination of FGA145, FGA146 and FGA147 against M^{pro} measured using the plate read assay. All measurements were performed for 10 min and fluorescence readout in 30 s increments. **A FGA146** (blue) showed strong fluorescence with higher concentrations as can be observed by the higher values for t=0 s. **B** The measurement of A was repeated without addition of substrate to correct for possibly occurring bleaching effects. **C,D FGA145** (pink) and **FGA147** (green) showed no self-fluorescence.

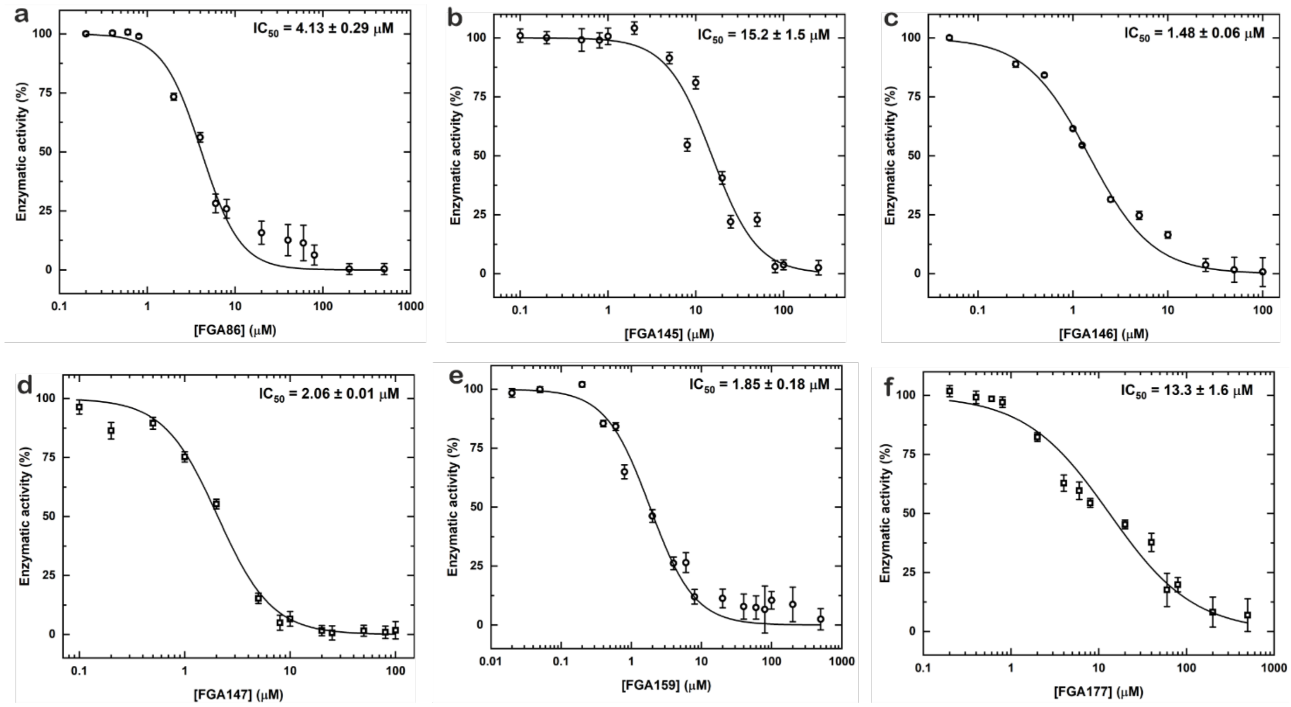

Figure 2. Inhibition of the M^{pro} enzymatic activity by the peptidyl nitroalkene compounds measured by the continuous fluorometric assay. Inhibition profiles for **FGA86** (**a**), **FGA145** (**b**), **FGA146** (**e**), **FGA147** (**d**), **FGA159** (**e**) and **FGA177** (**f**) against the SARS-CoV-2 M^{pro}. All data are mean values ± standard deviation of three technical replicates.

Table 2 Data collection and refinement statistics.

	FGA146	FGA147
Synchrotron (Beamline)	ESRF (ID30B)	ALBA (XALOC)
**Data collection**		
Space group	P 2_1	P $2_1 2_1 2$
Cell dimensions		
a, b, c (Å)	54.54, 98.71, 58.98	44.72, 62.66, 106.13
α, β, γ (˚)	90.00, 107.31, 90.00	90.00, 90.00, 90.00
Resolution(Å)[a]	48.97-1.98 (2.10-1.98)	41.21-1.62 (1.68-1.62)
Total reflections	270122 (39803)	496655 (76698)
Unique reflections	40887 (6215)	38449 (5978)
R_{merge}	0.125 (1.676)	0.059 (2.620)
R_{meas}	0.136 (1.822)	0.061 (2.728)
CC 1/2	99.7 (41.9)	99.9 (59.4)
Completeness (%)	98.7 (92.8)	99.4 (97.1)
$\langle I/\sigma(I)\rangle$	9.13 (1.04)	21.94 (0.99)
Wilson B-factor	47.04	39.09
Multiplicity	6.61 (6.40)	12.9 (12.8)
Monomers per ASU	2	1
**Refinement**		
R_{work}	0.192	0.190
R_{free}	0.232	0.243
Reflections used	38830	36608
No. atoms (non-hydrogens)	4922	2572
Protein	4687	2368
Ligands	70	32
Water	165	172
Protein residues	606	306
Average B factor (Å^2)	44.90	36.41
Protein atoms	44.73	36.00
Ligands	49.87	40.39
Water	47.50	41.45
R.m.s. deviations		
Bond lengths (Å)	0.010	0.012
Bond angles (˚)	1.611	1.768
Ramachandran statistics		
Favored (%)	96.51	98.03
Allowed (%)	3.16	1.64
Outliers (%)	0.33	0.33
Clashscore	3.32	3.61
PDB code	8BGA	8BGD

[a]Values in parentheses are for the highest-resolution shell.

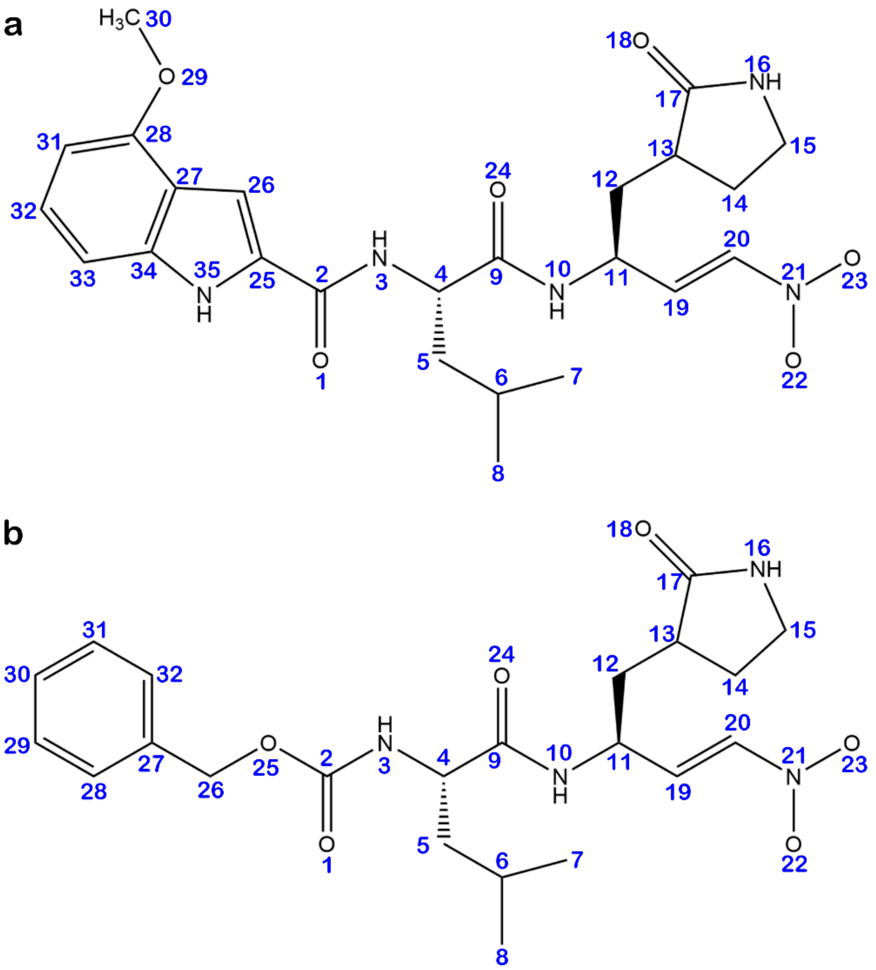

Figure 3. Molecular structure and numbering scheme for compounds **FGA146** (a) and **FGA147** (b).

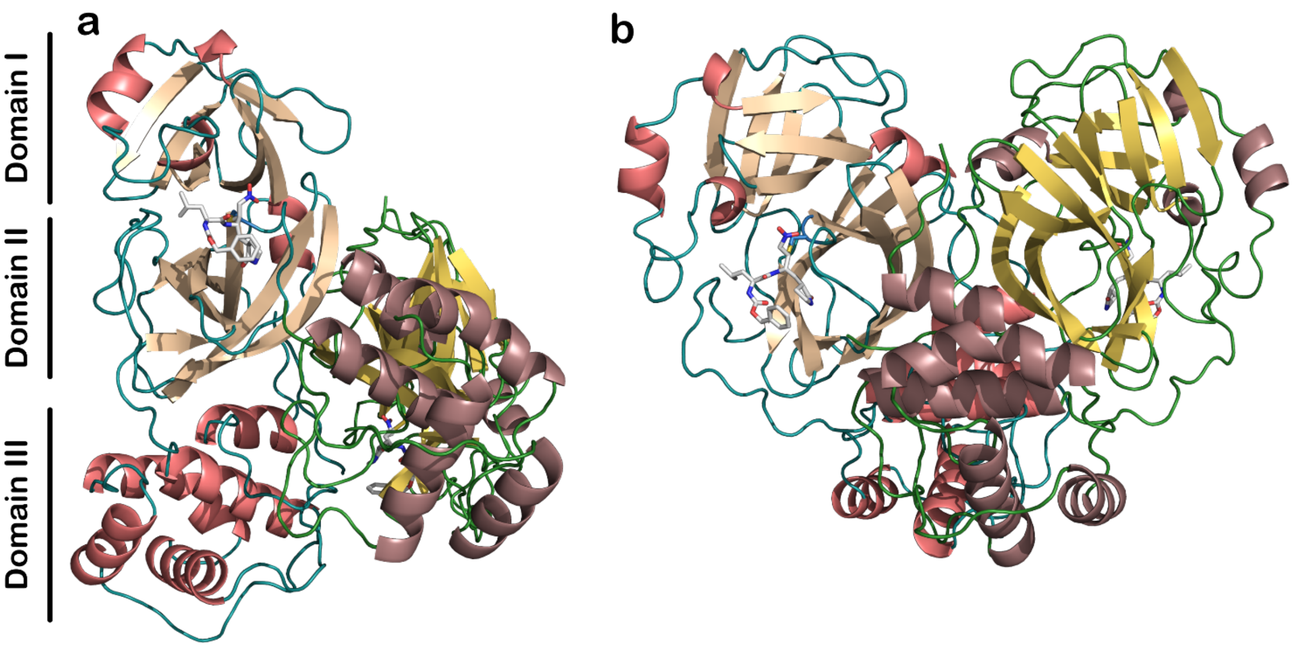

Figure 4. Domain structure of the M^{pro}. a Structure of M^{pro} in complex with **FGA147** with indication of the three domains of the monomer. The active site is made up by domains I and II, while domain III is the dimerization domain. **b** Rotation of 90 ° where it can be observed domains I and II from both monomers in the upper side and the two domains III one in front of the other.

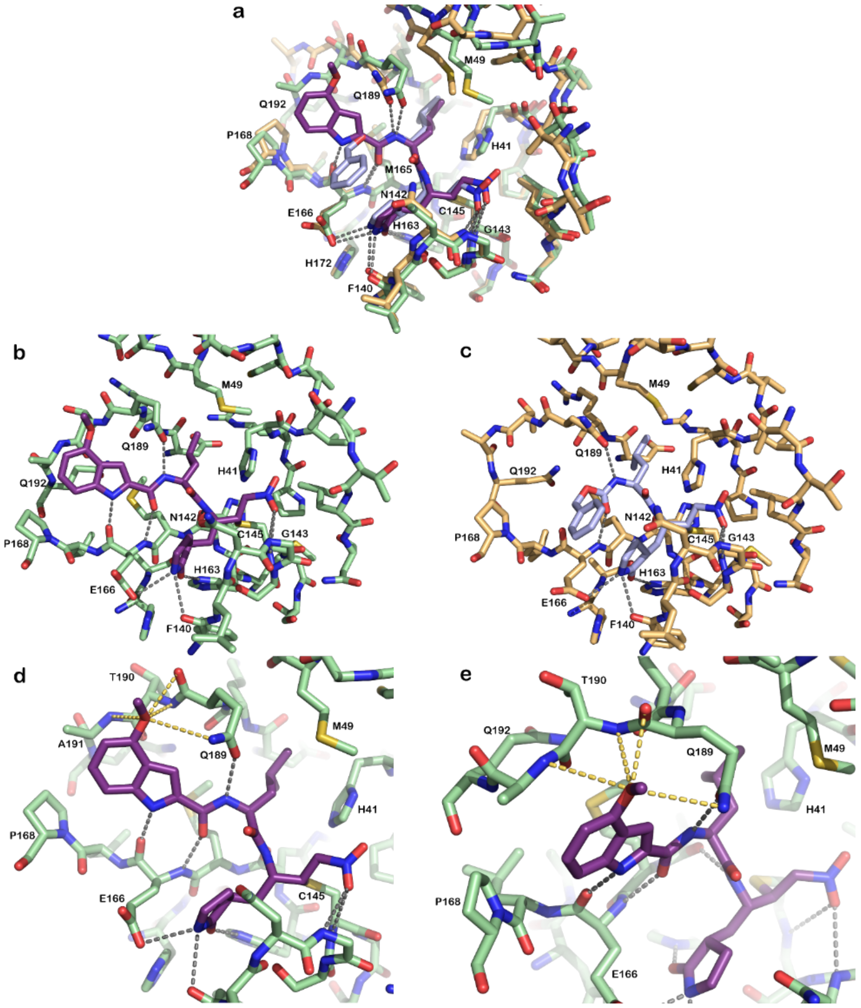

Figure 5. Interactions FGA146 and FGA147 with the active site of M^{pro}. a Active site superposition of the structures of M^{pro} in complex with **FGA146** (Inhibitor in violet and protein residues in light green) and **FGA147** (Inhibitor in light blue and protein residues in yellow orange). **b** Active site of the structure of M^{pro} in complex with **FGA146**. **c** Active site of the structure of M^{pro} in complex with **FGA147**. **d FGA146** encased into the S4 site. **e** Interactions of **FGA146** with the S4 site. Yellow dashed lines show how the methoxy group is trapped by residues 189-191 of the protein without hydrogen bond interactions, due to long distance or improper geometry. Dashed lines represent interactions between protein atoms and inhibitor atoms. Gray dashed lines represent hydrogen bond interactions. Distances and description of the interactions are shown in Table S3.

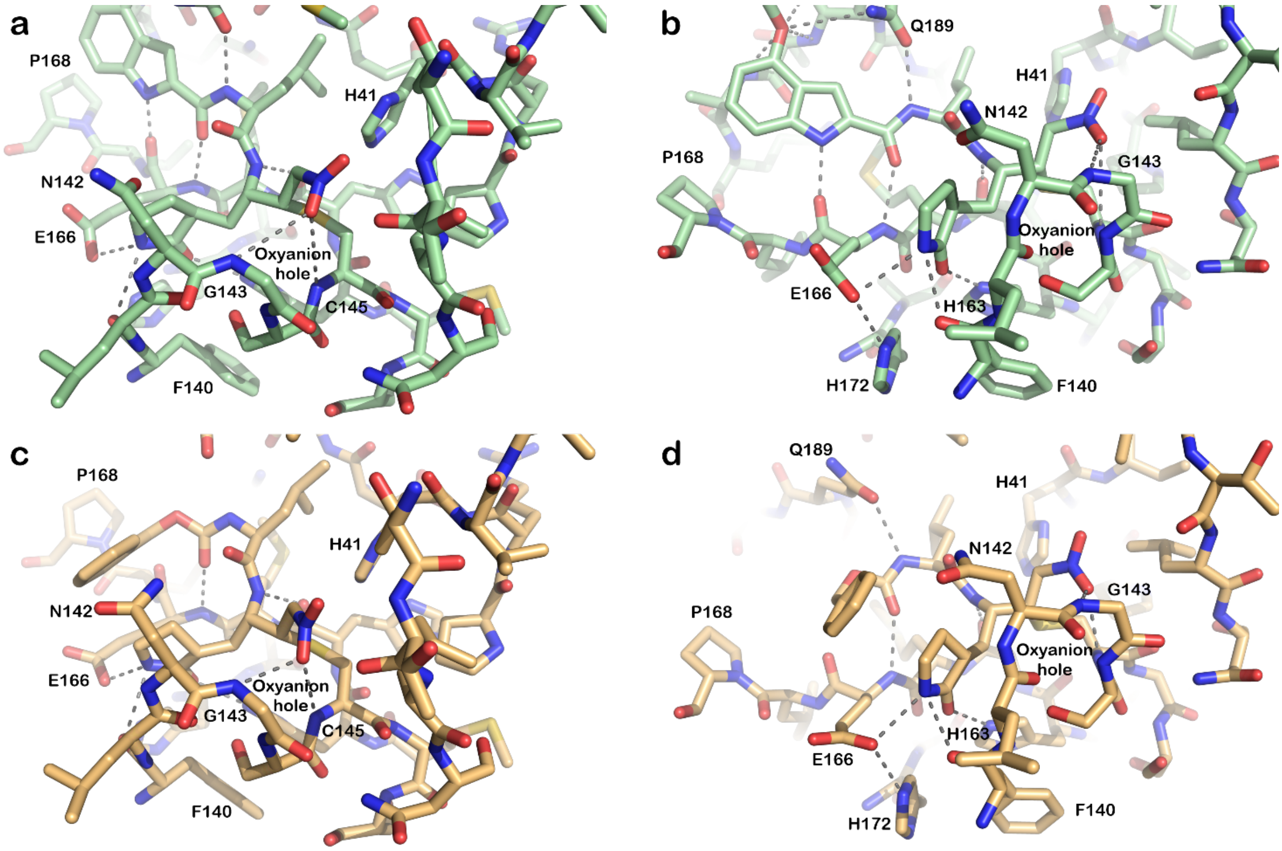

Figure. 6 Interactions between protein atoms and inhibitor atoms. The nitro group of the warhead is located in between the "oxyanion hole" made up by Gly143, Ser144 and Cys145, and His41 that together with Cys145 forms the catalytic dyad (**a**,**c**). Interactions of residues in positions P1 and P3 with protein atoms (**b**,**d**). Panels **a** and **b** show the structure of M^{pro} in complex with **FGA146** and panels **c** and **d** show the structure of M^{pro} in complex with **FGA147**.

Table 3 Interactions between residues of the M^{pro} active site and the inhibitors.

Protein residue	Protein atom	FGA146	Distance (Å) Mon. A	Mon. B	FGA147	Distance (Å)
C145	N	O23	3.87	3.25#	O22	3.13#
G143	N	O23	3.58	2.97#	O22	2.99#
F140	O	N16	3.27#	3.26#	N16	3.18#
H41	NE2	O22	3.42#	4.05	O23	4.14
E166	OE1	N16	3.22#	2.99#	N16	2.95#
H163	NE2	O18	2.72#	2.75#	O18	2.68#
H164	O	N10	2.93#	2.81#	N10	2.92#
Q189	OE1	N3	2.79#	3.09#	N3	2.98#
E166	N	O1	2.87#	2.86#	O1	2.94#
E166	O	N35	2.56#	2.56#		
Q189	O	O29	3.37	3.42		
Q189	NE2	O29	3.98	4.63		
T190	N	O29	3.56	3.30		
A191	N	O29	4.01	3.91		

Predicted hydrogen bond interactions between protein atoms and inhibitor atoms.

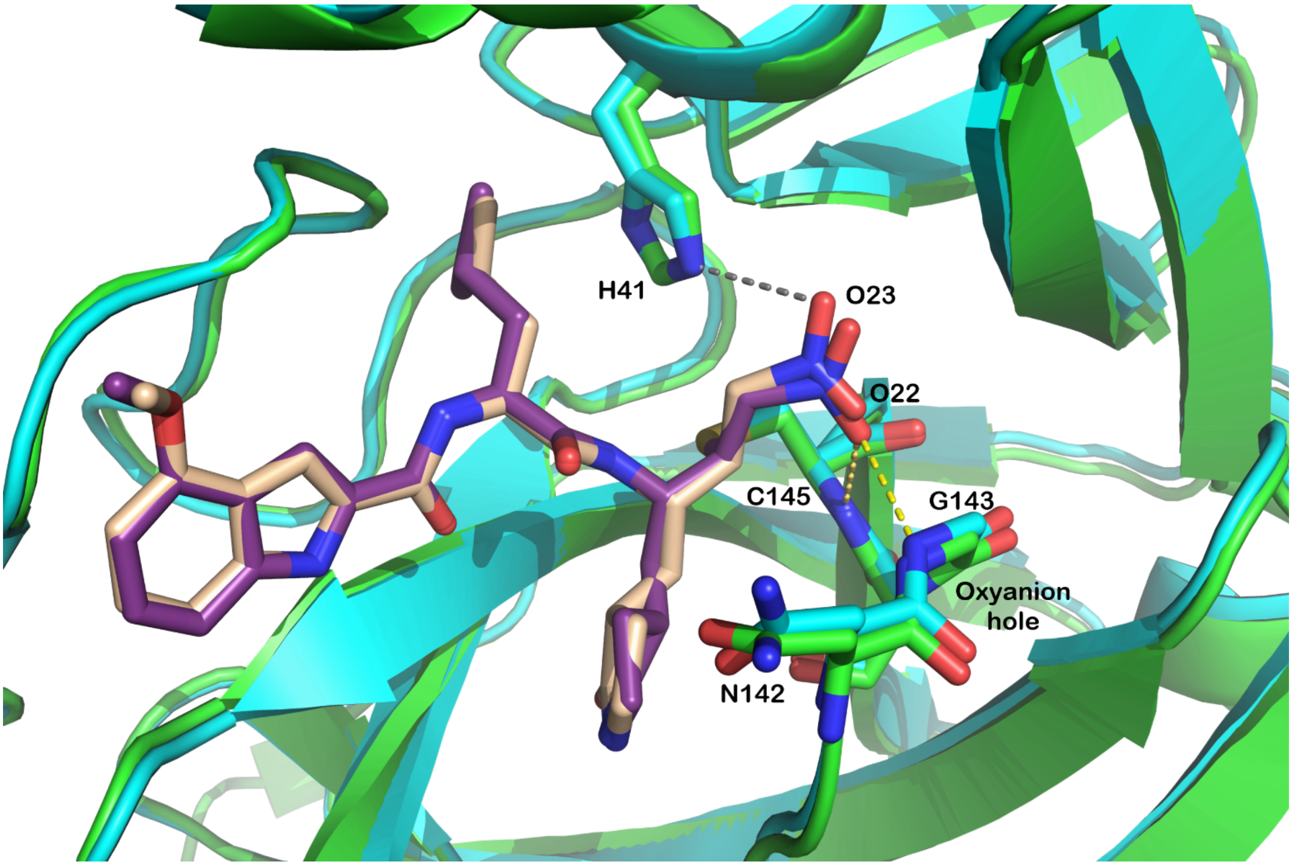

Figure 7 Interaction of the warhead of FGA146 with His41. The two monomers of M^{pro} interact differently with the nitro group of the warhead. The nitro group of the inhibitor in monomer A (brown) moves away from the oxyanion hole and interacts with His41 (gray dashed line) while this group in monomer B (purple) is located closer to the residues of the "oxyanion hole" and interacts with them through two hydrogen bonds (yellow dashed lines).

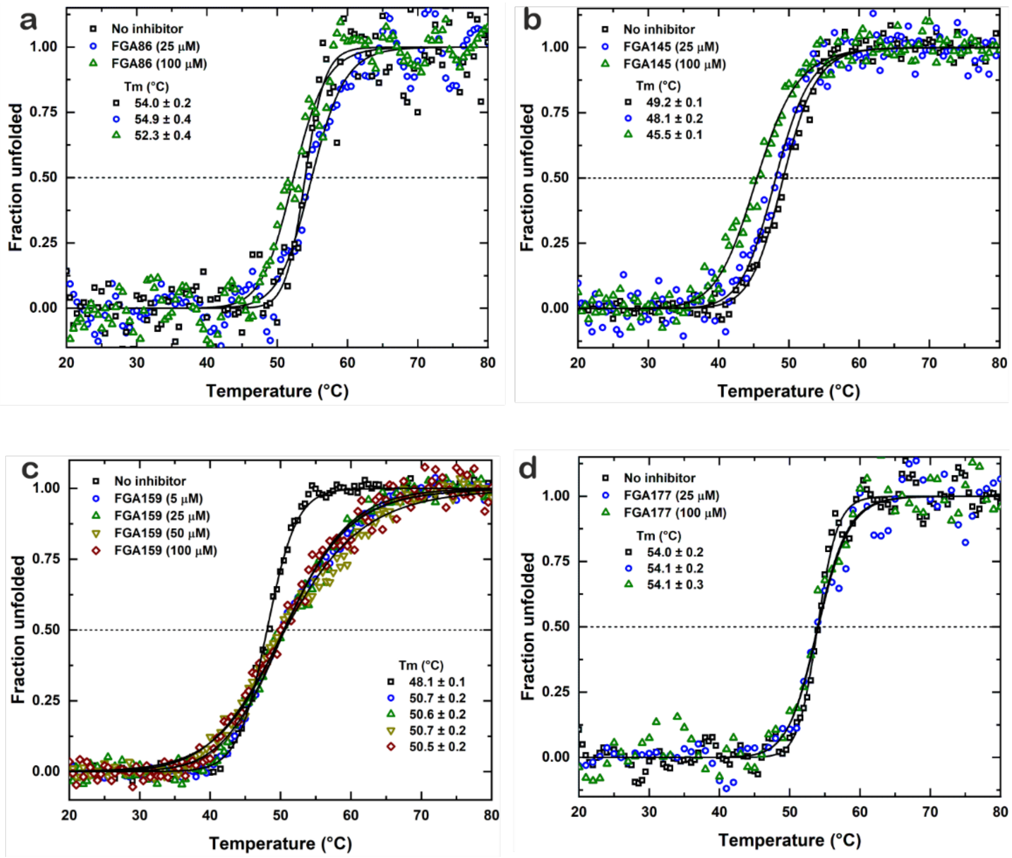

Figure 8 Effect of the inhibitors on the thermal denaturation of M^{pro}.

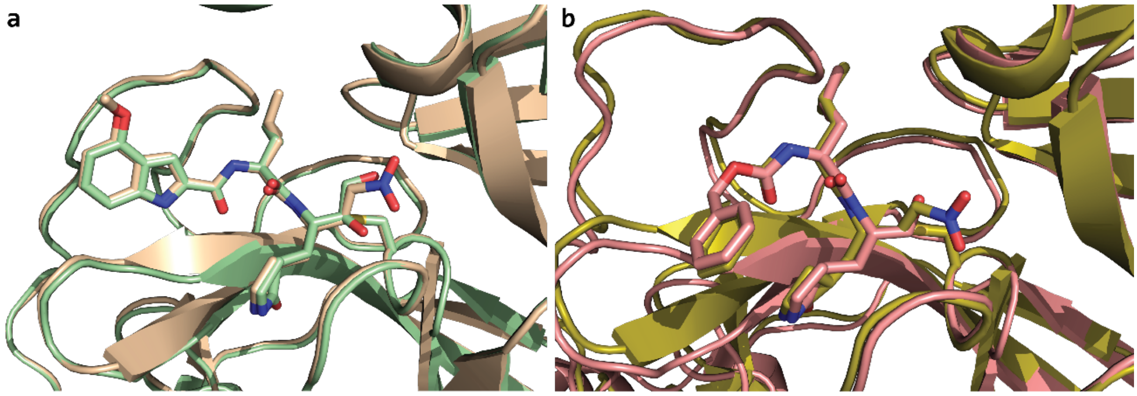

Figure 9. Comparison of the M^{pro} structure in complex with FGA146 and FGA147 with those of GC376 and PF-00835231. a) Structural alignment of M^{pro} in complex of FGA146 (wheat) and in complex with PF-00835231 (8DSU, pale green)[21]. b) Structural alignment of M^{pro} in complex of FGA147 (olive) and in complex with GC376 (7CB7, salmon)[22].

SYNTHESIS

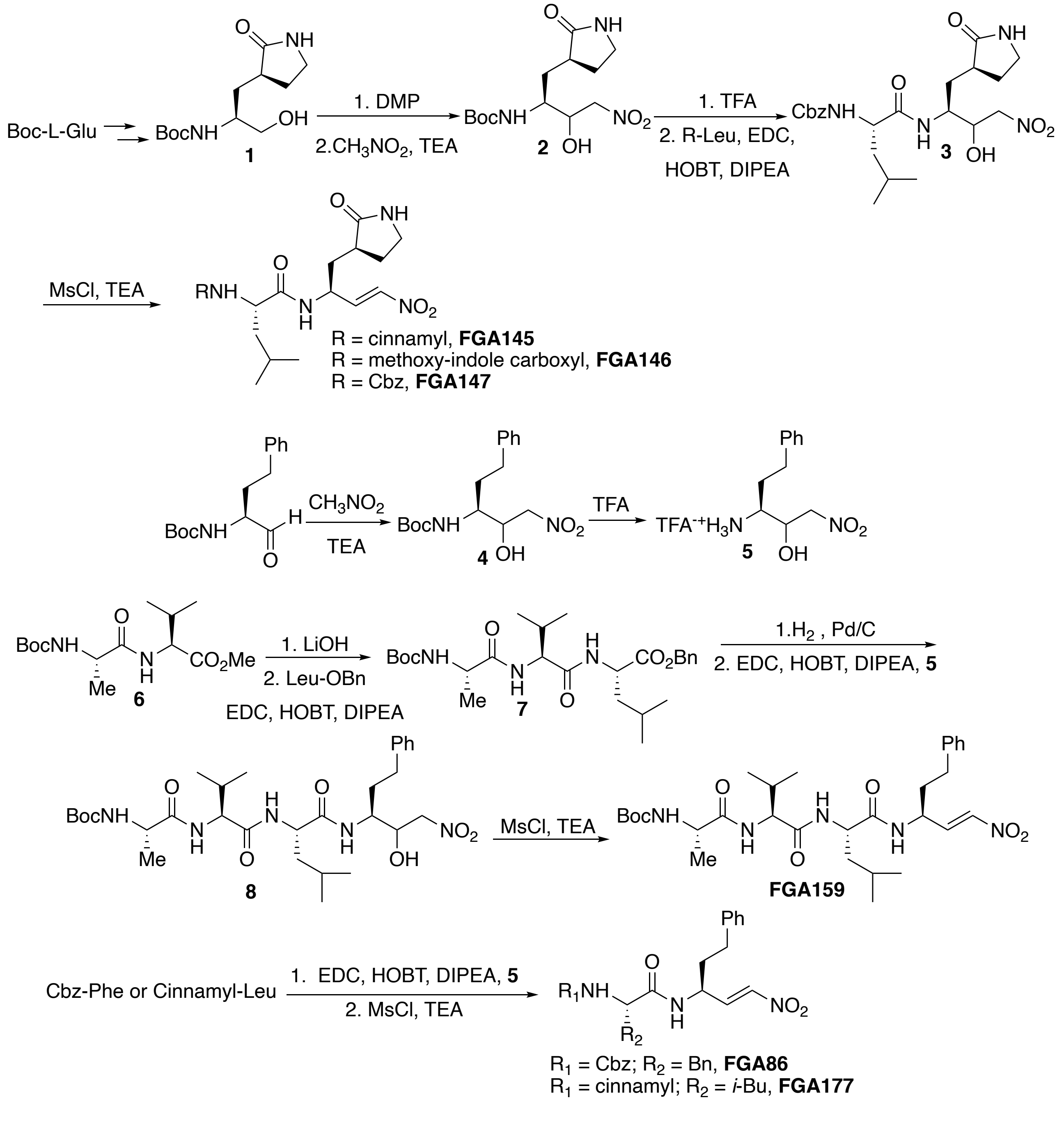

General information

Unless otherwise specified, all reactions were carried out under nitrogen atmosphere with magnetic stirring. All solvents and reagents were obtained from commercial sources and were purified according to standard procedures before use. ^{1}H NMR spectra and ^{13}C NMR spectra were measured in $CDCl_3$ (^{1}H, 7.24 ppm; ^{13}C 77.0 ppm) or in CD_3OD (^{1}H, 3.35, 4.78 ppm; ^{13}C 49.3 ppm) solution at 30 °C on a 300 MHz or a 400 MHz NMR spectrometer. Mass spectra were measured in a QTOF I (quadrupole-hexapole-TOF) mass spectrometer with an orthogonal Z-spray electrospray interface.

EM Science Silica Gel 60 was used for column chromatography while TLC was performed with precoated plates (Kieselgel 60, F254, 0.25 mm).

General procedure for the preparation of nitroalkenes

To a stirred solution of alcohol **1** (1.05 g, 4 mmol) in dichloromethane (32 mL) was added Dess-Martin periodinane (1.82 g, 4.3 mmol) and sodium bicarbonate (361mg, 4.3 mmol). The resulting mixture was stirred at room temperature for 1 h. Then the reaction mixture was cold with an ice-bath and triethylamine (0.17 mL, 1.21 mmol) and nitromethane (1.33 mL, 24.4 mmol) were. Then the mixture was stirred for 15 h at room temperature and then was quenched with a saturated aqueous solution of NH_4Cl (10 mL), the mixture was extracted with CH_2Cl_2 (3 x 15 mL) and the combined organic layers were washed with HCl 1M, then with a saturated aqueous solution of sodium bicarbonate and then dried over Na_2SO_4. Then the solvent was evaporated and the residue was purified by column chromatography (silica gel, CH_2Cl_2/MeOH (99:1 to 85:15) to afford the desired product as a yellow oil (71%).

The corresponding nitroaldol (0.73 mmol) was dissolved in dichloromethane (2.1 mL) and placed in an ice bath. Then trifluoroacetic acid (1.1 mL) in dichloromethane (1.1 mL) was added dropwise and the mixture was stirred at room temperature for 3 h. The reaction mixture was evaporated in vacuo to give the product as a colorless solid. The resulting mixture was submitted to the next step without any further purification.

To a solution of the ammonium trifluoroacetate (0.80 mmol) and the carboxylic acid (0.89 mmol) in dichloromethane (8 mL) cold with an ice-bath, HOBt·H_20 (121 mg, 0.89 mmol) was added. After 15 min at the same temperature, DIPEA (0.56 mL, 3.23 mmol) was added dropwise. After another 15 min, EDC (186.2 mg, 0.97 mmol) was added and the mixture was stirred for 16 h at room temperature. Then the mixture was quenched with saturated ammonium chloride solution (10 mL) and extracted with dichloromethane (3 x 20 mL). The combined organic layers were washed with HCl 1M, with a saturated aqueous sodium bicarbonate solution and then dried over Na_2SO_4. Then the solvent was evaporated and the residue was purified by column chromatography (silica gel, CH_2Cl_2/MeOH (100:0 to 85:15) to afford the desired product (64%, two steps).

To an ice bath cold solution of peptidyl nitroaldol (0.66 mmol) in dichloromethane (6.6 mL) was added DIPEA (0.24 mL, 1.39 mmol), then methanesulfonyl chloride (0.056 mL, 0.73 mmol). The resulting mixture was stirred overnight, then it was quenched with a saturated aqueous solution of NH_4Cl (10 mL) and extracted with dichloromethane (3 x 20 mL). The combined organic layers were washed with HCl 1M then with a saturated aqueous sodium bicarbonate solution and then dried over

Na_2SO_4. Then the solvent was evaporated and the residue was purified by column chromatography (silica gel, CH_2Cl_2/MeOH (99:1 to 9:1) to afford the desired product (68-81%).

For the preparation of all the compounds, the coupling steps and nitroalkene formation were done following the experimental procedure detailed above.

For the preparation of compound **FGA159**, the hydrolysis and hydrogenation steps were done following standard experimental procedures.

***tert*-Butyl ((2*S*)-1-hydroxy-3-(2-oxopyrrolidin-3-yl)propan-2-yl)carbamate 1**
$[\alpha]^D_{25}$ = -22.0° (c = 1, MeOH)
white solid (m p 152–153 °C)
^{1}H NMR (400 MHz, MeOD) δ 6.48 (d, *J* = 8.8 Hz, 1H), 3.68 - 3.60 m, 1H), 3.54 (dd, *J* = 10.9, 5.2 Hz, 2H), 3.47 (dd, *J* = 10.9, 6.2 Hz, 2H), 3.39 - 3.29 (m, 2H), 2.50 - 2.36 (m, 2H), 1.90 - 1.78 (m, 2H), 1.62 - 1.51 (m, 1H), 1.46 (s, 9H) ppm.
^{13}C NMR (101 MHz, MeOD) δ 181.37, 156.96, 78.64, 64.48, 50.46, 40.16, 38.31, 32.64, 27.49, 27.43 ppm.
IR δ 3450, 3265, 3250, 1685, 1542, 1291, 1304, 1257, 902, 723, 654 cm^{-1}.
HRMS (ESI) calcd for $C_{12}H_{23}N_2O_4$ $[M+H]^+$=259.1653 found $[M+H]^+$=259.1658 (1.9 ppm)

***tert*-Butyl ((2*S*)-3-hydroxy-4-nitro-1-(2-oxopyrrolidin-3-yl)butan-2-yl)carbamate 2.**
white solid (m p 62–64 °C)
^{1}H NMR (400 MHz, CD3OD) δ 4.67–4.53 (m, 1H), 4.43–4.28 (m, 2H), 3.85–3.76 (m, 1H), 2.50 (ddd, *J* = 18.2, 8.6, 4.8 Hz, 1H), 2.34 (tdd, *J* = 12.6, 5.7, 3.9 Hz, 1H), 2.09 (dt, *J* = 14.1, 5.2 Hz, 1H), 1.91 (dq, *J* = 12.5, 9.0 Hz, 1H), 1.66–1.53 (m, 1H), 1.47 (s, 9H) ppm.
^{13}C NMR (101 MHz, CD3OD) δ 180.8, 156.5, 79.1, 78.7, 69.7, 51.6, 40.1, 39.0, 32.1, 27.4, 27.3 ppm.
HRMS (ESI) calcd for $[M+Na]^+$=340.1485 found $[M+Na]^+$=340.1490 (1.5 ppm)

Benzyl ((2*S*)-1-(((2*S*)-3-hydroxy-4-nitro-1-(2-oxopyrrolidin-3-yl)butan-2-yl)amino)-4-methyl-1-oxopentan-2-yl)carbamate 3.
white solid (m p 185–187 °C)
^{1}H NMR (300 MHz, CD3OD) δ 4.66-3.86 (m, 5H), 3.38-3.24 (m, 2H), 2.49–2.19 (m, 3H), 2.05–1.66 (3H, m), 1.52–1.57 (m, 2H), 1.47 (s, 9H), 1.00–0.94 (m, 6H) ppm.
^{13}C NMR (75 MHz, CD3OD) δ 181.1, 175.1, 156.5, 79.4, 78.7, 70.2, 53.8, 49.0, 40.1, 38.1, 32.6, 31.4, 27.4, 24.7, 22.0, 20.6 ppm.
HRMS (ESI) calcd for [M+Na]+=469.2063 found $[M+Na]^+$=469.2071 (1.7 ppm)

***tert*-Butyl ((3*S*)-2-hydroxy-1-nitro-5-phenylpentan-3-yl)carbamate 4.**
Viscous oil
^{1}H NMR (400 MHz, CD3OD) δ 7.28–7.15 (m, 5H), 6.29 (d, J = 9.3 Hz, 1H), 4.60–4.51 (m, 1H), 4.44–4.17 (m, 2H), 3.63–3.50 (m, 1H), 2.80–2.55 (m, 2H), 1.91–1.67 (m, 2H), 1.48 (s, 9H) ppm.
^{13}C NMR (101 MHz, CD3OD) δ 156.9, 141.6, 128.1, 125.7, 79.3, 79.0, 70.4, 53.5, 52.4, 33.1, 27.6 ppm.
HRMS (ESI) calcd for $[M+Na]^+$= 347.1583 found $[M+Na]^+$= 347.1578 (1.4 ppm)

Methyl (*tert*-butoxycarbonyl)-*L*-alanyl-*L*-valinate 6.
white solid (m p 88–90 °C)
$[\alpha]^D_{25}$ = -76.61° (c = 0.60, MeOH)
IR δ 3327, 2951, 2844, 2362, 1657, 1537, 1450, 1368, 1253, 1270, 1172, 1015, 472 cm^{-1}.

^{1}H NMR (400 MHz, $CDCl_3$) δ 6.58 (br s, 1H), 4.92 (br s, 1H), 4.46 (dd, *J* = 8.9, 5.0 Hz, 1H), 4.24–3.99 (m, 1H), 3.67 (s, 3H), 2.18–2.03 (m, 1H), 1.38 (s, 9H), 1.29 (d, *J* = 7.1 Hz, 3H), 0.86 (d, *J* = 6.9 Hz, 3H), 0.84 (d, *J* = 6.9 Hz, 3H) ppm.
^{13}C NMR (101 MHz, $CDCl_3$) δ 172.5, 172.2, 155.6, 80.2, 57.1, 52.1, 50.1, 31.3, 28.3, 18.9, 17.7 ppm.
HRMS (ESI) calcd for $C_{14}H_{26}N_2NaO_5^+$ $[M+Na]^+$=325.1739, found $[M+Na]^+$=325.1734 (1.5 ppm)

Benzyl (*tert*-butoxycarbonyl)-*L*-alanyl-*L*-valyl-*L*-leucinate 7.
white solid (m p 68–72 °C)
$[\alpha]^D{}_{25}$ = -116.24° (c = 0.54, MeOH)
IR δ 3302, 2946, 2833, 2367, 1656, 1643, 1427, 1118, 1020, 622, 516 cm^{-1}.
^{1}H NMR (400 MHz, CD3OD) δ 6.53–6-67 (m, 5H), 3.73–3.80 (m, 1H), 4.44 (d, *J* = 12.2 Hz, 1H), 4.40 (d, *J* = 12.2 Hz, 1H), 3.33–3.83 (m, 1H), 3.47 (d, *J* = 7.2 Hz, 1H), 0.87–0.97 (m, 3H), 1.29 (dq, *J* = 13.6, 6.8 Hz, 1H), 0.55 (d, *J* = 7.2 Hz, 3H), 0.14–0.21 (m, 12H) ppm.
^{13}C NMR (101 MHz, CD3OD) δ 175.7, 173.6, 173.5, 157.8, 137.2, 129.6, 129.5, 129.4, 80.4, 67.9, 59.5, 52.2, 41.4, 32.4, 28.7, 25.9, 23.2, 21.8, 19.7, 18.5, 18.1 ppm.
HRMS (ESI) calcd for $C_{26}H_{41}N_3NaO_6^+$ $[M+Na]^+$=514.2893, found $[M+Na]^+$=514.2895 (0.4 ppm)

***tert*-Butyl ((2*S*)-1-(((2*S*)-1-(((2*S*)-1-(((3*S*)-2-hydroxy-1-nitro-5-phenylpentan-3-yl)amino)-4-methyl-1-oxopentan-2-yl)amino)-3-methyl-1-oxobutan-2-yl)amino)-1-oxopropan-2-yl)carbamate 8.**
white solid (m p 196–198 °C)
^{1}H NMR (400 MHz, CD3OD) δ 7.38–7.06 (m, 5H), 4.66–3.75 (m, 7H), 2.79–2.67 (m, 1H), 2.65–2.51 (m, 1H), 2.15 (dd, *J* = 8.8, 6.4 Hz, 1H), 1.94 (dd, *J* = 14.8, 7.7 Hz, 1H), 1.77–1.63 (m, 2H), 1.52–1.41 (m, 9H), 1.31 (d, J = 7.2 Hz, 3H), 1.05–0.91 (m, 12H) ppm.
HRMS (ESI) calcd. for $[M+Na]^+$=630.3479 $[M+Na]^+$=630.3488 (1.4 ppm)

FGA86
white solid (m p 150–153 °C)
$[\alpha]^D{}_{25}$ = +46.82° (c = 0.71, MeOH)
IR δ 3298, 2947, 2836, 2198, 1647, 1529, 1453, 1400, 1119, 1018, 697, 501 cm^{-1}.
^{1}H NMR (400 MHz, CD_3OD) δ 7.08-7.34 (m, 15H), 5.12 (d, *J* = 12.8 Hz, 1H), 5.08 (d, *J* = 12.4 Hz, 1H), 4.51 (ddt, *J* = 14.0, 9.3, 4.8 Hz, 1H), 4.39 (t, *J* = 7.8 Hz, 1H), 2.95–3.08 (m, 2H), 2.56–2.75 (m, 1H), 2.32 (t, *J* = 7.7 Hz, 1H), 1.65–1.96 (m, 2H) ppm.
^{13}C NMR (101 MHz, CD3OD) δ 172.41, 156.57, 139.95, 139.47, 136.72, 129.06, 128.24, 128.17, 128.14, 128.04, 127.57, 126.76, 126.63, 125.71, 66.54, 57.21, 34.99, 34.64, 31.62 ppm.
HRMS (ESI) calcd. for $C_{28}H_{30}N_3O_5^+$ $[M+H]^+$ = 488.2185, found 488.2174 (2.3 ppm)

FGA145
white solid (m p 107–109 °C)
$[\alpha]^D{}_{25}$ = -21.05° (c = 0.57, MeOH)
IR δ 3072, 3069, 2945, 2892, 2370, 1655, 1643, 1555, 1353, 1160, 1216, 980, 768, 690, 578 cm^{-1}.
^{1}H NMR (400 MHz, $CDCl_3$) δ 7.38–7.41 (m, 3H), 7.24–6.99 (m, 5H), 6.53 (d, *J* = 15.9 Hz, 1H), 4.59 (td, *J* = 11.4, 4.7 Hz, 1H), 4.29 (dd, *J* = 9.3, 5.6 Hz, 1H), 3.16–3.18 (m, 2H), 2.28 (ddd, *J* = 13.6, 9.7, 4.3 Hz, 1H), 2.14 (tdd, *J* = 15.5, 8.9, 3.2 Hz, 1H), 1.92 (ddd, *J* = 16.0, 12.1, 4.5 Hz, 1H), 1.43-1.74 (m, 5H), 0.84 (d, *J* = 6.4 Hz, 3H), 0.80 (d, *J* = 6.4 Hz, 3H) ppm.
^{13}C NMR (75 MHz, CD3OD) δ 190.53, 180.27, 173.83, 173.73, 167.40, 141.27, 140.95, 140.07, 135.32, 134.89, 129.54, 128.56, 127.50, 120.05, 52.68, 46.02, 40.48, 40.10, 38.34, 34.24, 27.25, 24.73, 21.95, 20.67 ppm.
HRMS (ESI) calcd. for $C_{23}H_{31}N_4O_5^+$ $[M+H]^+$ = 443.2294, found 443.2288 (1.4 ppm)

FGA146

white solid (m p 140–142 °C)

$[\alpha]^{D}_{25}$ = -6.62° (c = 0.56, MeOH)

IR δ 3293, 2957, 2941, 2878, 2360, 1672, 1662, 1634, 1430, 1357, 1257, 1101, 1099, 764 cm^{-1}.

^{1}H NMR (400 MHz, CD3OD) δ 7.03–7.19 (m, 4H), 6.93 (d, *J* = 8.3 Hz, 1H), 6.41 (d, *J* = 7.6 Hz, 1H), 4.65–4.68 (m, 1H), 4.48 (dd, *J* = 9.7, 5.2 Hz, 1H), 3.83 (s, 3H), 3.09–3.19 (m, 2H), 2.50 (ddd, *J* = 18.6, 9.9, 4.4 Hz, 1H), 2.18 (dddd, *J* = 12.4, 8.6, 6.8, 2.6 Hz, 1H), 2.00 (ddd, *J* = 14.0, 11.5, 4.4 Hz, 1H), 1.55–1.74 (m, 5H), 0.93 (d, *J* = 6.2 Hz, 3H), 0.89 (d, *J* = 6.3 Hz, 3H) ppm.

^{13}C NMR (101 MHz, CD3OD) δ 180.49, 173.91, 162.78, 154.28, 141.26, 139.85, 138.44, 128.85, 124.96, 118.72, 104.78, 101.73, 98.97, 54.32, 52.48, 45.93, 40.25, 40.05, 38.21, 34.18, 27.32, 24.83, 21.95, 20.63 ppm.

HRMS (ESI) calcd. for $C_{24}H_{32}N_5O_6^+$ $[M+H]^+$ = 486.2353, found 486.2353 (0.0 ppm)

FGA147

Pale yellow solid (m p 69–71 °C)

$[\alpha]^{D}_{25}$ = -12.69° (c = 0.53, MeOH)

IR δ 3325, 2949, 1665, 1558, 1452, 1401, 1267, 1118, 1021, 698, 601 cm^{-1}.

^{1}H NMR (400 MHz, CD3OD) δ 7.48–7.09 (m, 5H), 5.26–5.02 (m, 2H), 4.93–4.78 (m, 1H), 4.26–4.06 (m, 1H), 3.50–3.12 (m, 2H), 2.64–2.06 (m, 3H), 1.90–1.49 (m, 5H), 1.05–0.86 (m, 6H) ppm.

^{13}C NMR (101 MHz, CD3OD) δ 180.1, 174.2, 157.1, 141.4, 139.8, 136.9, 128.1, 127.6, 127.4, 66.4, 54.1, 45.8, 40.5, 40.1, 38.2, 34.3, 27.9, 24.6, 22.0 ppm.

HRMS (ESI) calcd. for $C_{22}H_{30}N_4NaO_6^+$ $[M+Na]^+$=469.2063, found $[M+Na]^+$=469.2071 (1.7 ppm)

FGA159

white solid (m p 172–175 °C)

$[\alpha]^{D}_{25}$ = -19.37° (c = 1.04, MeOH)

IR δ 3282, 2959, 2929, 2867, 1637, 1533, 1455, 1350, 1255, 1171, 1019, 701, 509 cm^{-1}.

^{1}H NMR (400 MHz, CD3OD) δ 7.21–6.99 (m, 5H), 4.55–4.44 (m, 1H), 4.34 (dd, *J* = 11.2, 3.3 Hz, 1H), 4.02 (d, *J* = 5.3 Hz, 1H), 3.87 (dd, *J* = 13.9, 6.9 Hz, 1H), 2.67 (ddd, *J* = 14.3, 8.5, 6.0 Hz, 1H), 2.56 (dt, *J* = 13.9, 8.2 Hz, 1H), 2.06 (td, *J* = 12.8, 6.5 Hz, 1H), 1.91 (m, 2H), 1.67–1.50 (m, 3H), 1.36 (s, 9H), 1.19 (d, *J* = 7.2 Hz, 1H), 0.95–0.78 (m, 12H) ppm.

^{13}C NMR (101 MHz, CD3OD) δ 175.6, 173.1, 172.3, 156.9, 141.3, 140.7, 139.8, 128.2, 128.1, 128.0, 127.9, 125.8, 79.6, 59.7, 52.1, 51.5, 40.0, 34.6, 31.6, 30.0, 27.4, 24.5, 22.1, 20.1, 18.1, 17.4, 16.1 ppm.

HRMS (ESI) calcd. for $C_{35}H_{48}N_5O_7^+$ $[M+H]^+$ = 590.3554, found 590.3547 (1.2 ppm)

FGA177

white solid (m p 162–164 °C)

$[\alpha]^{D}_{25}$ = -56.76° (c = 0.48, MeOH)

IR δ 3266, 2957, 2925, 2359, 1650, 1613, 1555, 1531, 1351, 1228, 749, 702 cm^{-1}.

^{1}H NMR (300 MHz, $CDCl_3$) δ 7.59 (d, *J* = 15.6 Hz, 1H), 7.44–6.96 (m, 15H), 6.37 (d, *J* = 15.6 Hz, 1H), 6.07 (d, *J* = 8.0 Hz, 1H), 4.68–4.58 (m, 1H), 4.56–4.47 (m, 1H), 2.72–2.55 (m, 2H), 1.72–1.57 (m, 3H), 0.90 (d, *J* = 6.0 Hz, 2H), 0.87 (d, *J* = 6.0 Hz, 3H) ppm.

^{13}C NMR (101 MHz, CD3OD) δ 173.5, 167.3, 141.3, 141.0, 140.0, 139.8, 134.9, 128.6, 128.2, 128.1, 127.5, 120.0, 52.3, 40.6, 34.8, 31.5, 24.7, 21.9, 20.6 ppm.

HRMS (ESI) calcd. for $C_{26}H_{31}N_3NaO_4^+$ $[M+Na]^+$ = 472.2212, found: 472.2210

Computational Methods

The coordinates for the starting point were taken from the X-Ray structure of the SARS-CoV-2 M^{pro} complexed with the N3 inhibitor (PDB ID 6LU7)[23]. Biological assemble (homodimer) was build using Discovery Studio Visualizer 19. The N3 inhibitor was then manually modified leading to the two new enzyme-inhibitor models. This X-Ray structure was already used in our laboratory to carry out different computational studies related to the SARS-CoV-2 M^{pro} [24-26]. The missing force field parameters for each model were generated using the Antechamber program[27], available in the AmberTools package (see Table S4 and S5). The protonation states of the titratable amino acids were determined using the empirical program PropKa ver. 3.0.3[28], while the histidine residues were assigned by detailed visual inspection. See a list of all the pKa values in Table S6. Then, each model was neutralized by adding 8 sodium counter ions and was placed in a box of 92.154 × 102.242 × 97.285 $Å^3$ of TIP3P[29] water molecules for both models, (generated using the solvateBox command of the tLeap facility with a value of 10 Å for the *distance* and 0.8 Å for the *closeness*).

The next step for each model consisted of 10^5 steps of conjugate-gradient minimization, followed by series of molecular dynamics (MD) simulations in the NVT ensemble with the AMBER ff03 force field[30], as implemented in NAMD software:[31] 100 ps for heating up to 310 K, followed by 10 ns of equilibration, using the Langevin thermostat[32]. All simulations made use of the PME algorithm for the electrostatic interactions with a force-switch scheme ranging from 14.5 to 16 Å, and a time step of 1 fs. Analysis of the time evolution of the root-mean-square deviations (RMSD) of the backbone atoms of the protein models (see Figure S10), using the cpptraj facility[33], confirmed that the two models become equilibrated.

QM/MM simulations.

After setting up the molecular models, an additive hybrid QM/MM scheme was employed for constructing of the total Hamiltonian, where the total energy is obtained as a sum of different terms:

$$E_{\frac{QM}{MM}} = E_{QM} + E_{\frac{QM}{MM}}^{elect} + E_{\frac{QM}{MM}}^{vdW} + E_{MM} \quad \text{(Eq. 3)}$$

In Equation (3), E_{QM} describes the atoms in the QM region, $E_{QM/MM}$ defines the interaction between the QM and MM region (both electrostatic and dispersion terms), and E_{MM} describes the rest of the MM region. The QM subset of atoms includes the P1' and P1 positions of the inhibitor, together with C145 and H41 residues of the protein. Four link atoms were inserted where the QM/MM boundary intersected covalent bonds in the positions indicated on Figure S11. Thus, QM part consisted of 57 atoms for both inhibitors. All the calculations were performed with the QMCube suite[34], for which the combination of the OpenMM and Gaussian09[35] programs was used for constructing the potential energy function. The AMBER ff03[30] and the TIP3P[29] force fields were selected to describe the MM

atoms, and the Minnesota functional M06-2X[36] with the split-valence 6-31+G(d,p) basis set[37] were used to treat the QM subset of atoms. This functional has been tested and shown to be suitable for modelling this type of reactivity[24-26,38-42]. The position of any atom over 20 Å from the substrate was fixed to speed up the calculations.

Reaction mechanisms for each inhibitor were initially explored using the nudged elastic band (NEB)[43] approach to set up plausible starting geometries for the transition structures. For this purpose, a total of 60 geometries were considered, derived from linear interpolation between the optimized geometries of the E:I and the E-I$^{(-)}$, and from the later to the E-I. Those geometries were restrained with the preceding and the subsequent ones using a harmonic constant of 200 kJ·mol^{-1}·Å^{-2}. Then, each energy maxima were characterized as a transition structure by hessian matrix inspection. The information obtained in this stage was used in the fine-tuning of the calculation of the free energy surface, in terms of potential of mean force (PMF). The PMF for each chemical step was obtained using the combination of the umbrella sampling (US) approach[44] with the weighted histogram analysis method (WHAM)[45]. Series of MD simulations were performed adding a restraint along the collective/path reaction coordinate *s,* with an umbrella force constant of 3000 kJ·mol^{-1}·Å^{-2}. In every window, QM/MM MD-NVT simulations were performed with a total of 4.75 ps at 310 K with a time step of 0.5 fs (a total of 9500 steps). The definition of the *s* coordinate was reduced to a combination of distances, following the equation:

$$s(\theta) \approx \frac{\sum_{i=0}^{N-1} i\delta_z e^{-\frac{|\theta-z|}{\delta_z}}}{\sum_{i=0}^{N-1} e^{-\frac{|\theta-z|}{\delta_z}}} \quad \begin{cases} \delta_z = \langle |z_{i+1} - z_i| \rangle = \frac{L}{N-1} \\ |\theta - z| = [(\theta - z)^T M^{-1} (\theta - z)]^{\frac{1}{2}} \qquad M_{i,j} = \sum_{k=1}^{3n} \frac{\partial\theta_i}{\partial x_k}\frac{\partial\theta_j}{\partial x_k} \end{cases} \qquad \text{(Eq. 4)}$$

were θ is defined as a set of distances (or internal coordinates in a broader sense), z follows the same formulation, but along the N milestones identifying the chemical transformation (remaining constant along all the simulations and being L the total length of z). The calculation of the distance between θ and z is performed in the metric space defined by the matrix M, which comprises the Jacobians of θ with respect to the cartesian coordinates of the involved atoms (x_k).

Since we are considering the same inhibition mechanism for both **FGA146** and **FGA147** inhibitors, the following internal coordinates were included in the s coordinate: d(Sγ, C_{19}), d(Sγ, Hγ), d(Hγ, Nε) and d(Hγ, C_{20}). All the information needed to define the equally distributed milestones were obtained from the analysis of the different NEBs previously traced.

Finally, the interaction energy was computed as a contribution of each residue of the protein to the interaction energy with the QM part of the substrate was computed using the following expression:

$$E_{\frac{QM}{MM}}^{Int} = \sum \left\langle \Psi \middle| \frac{q_{MM}}{r_{e,MM}} \middle| \Psi \right\rangle + \sum\sum \frac{Z_{QM} q_{MM}}{r_{QM,MM}} + E_{\frac{QM}{MM}}^{vdW} \qquad \text{(Eq. 5)}$$

This interaction energy can be exactly decomposed in a sum over residues provided that the polarized wave function (Ψ) is employed to evaluate this energy contribution. Because of the large number of structures that must be evaluated to obtain a representative population, the QM sub-set of atoms were described by the semiempirical Hamiltonian AM1[46] in these QM/MM MD calculations.

Table 4. Atom types, charges and parameters obtained for the inhibitor **FGA146** generated on the **E:I** complex using antechamber package included in AmberTools. The partial charges were obtained by means of AM1-BCC calculations with the SQM program from the same suite.

<table>
<tr><th>Atom name</th><th>Atom type</th><th>Charge</th><th>Parameters</th></tr>
<tr><td>C26</td><td>cc</td><td>-0.098200</td><td rowspan="50">NONBON
cc 1.9080 0.0860
ha 1.4590 0.0150
ca 1.9080 0.0860
os 1.6837 0.1700
c3 1.9080 0.1094
h1 1.3870 0.0157
na 1.8240 0.1700
hn 0.6000 0.0157
cd 1.9080 0.0860
c 1.9080 0.0860
o 1.6612 0.2100
n 1.8240 0.1700
hc 1.4870 0.0157
c2 1.9080 0.0860
h4 1.4090 0.0150
no 1.8240 0.1700

BOND
cc-ha 349.10 1.084
ca-cc 385.10 1.456
cc-cd 500.90 1.373
ca-ca 461.10 1.398
ca-os 376.60 1.370
c3-os 308.60 1.432
c3-h1 330.60 1.097
ca-ha 345.80 1.086
ca-na 420.50 1.384
hn-na 408.40 1.010
cd-na 425.80 1.380
c -cd 371.00 1.468
c -o 637.70 1.218
c -n 427.60 1.379
hn-n 403.20 1.013
c3-n 328.70 1.462
c3-c3 300.90 1.538
c -c3 313.00 1.524
c3-hc 330.60 1.097
c2-c3 326.80 1.510
c2-c2 569.40 1.334
c2-ha 343.10 1.088
c2-h4 344.60 1.087
c2-no 343.00 1.448
no-o 741.80 1.226

ANGLE
ca-ca-cc 65.000 120.790
cc-cd-na 73.400 106.990
c -cd-cc 65.100 121.350
ca-cc-ha 45.800 124.040
cd-cc-ha 48.500 121.760
ca-cc-cd 67.600 113.510</td></tr>
<tr><td>H15</td><td>ha</td><td>0.162000</td></tr>
<tr><td>C27</td><td>ca</td><td>-0.142800</td></tr>
<tr><td>C28</td><td>ca</td><td>0.187100</td></tr>
<tr><td>O29</td><td>os</td><td>-0.323900</td></tr>
<tr><td>C30</td><td>c3</td><td>0.112700</td></tr>
<tr><td>H01</td><td>h1</td><td>0.047367</td></tr>
<tr><td>H02</td><td>h1</td><td>0.047367</td></tr>
<tr><td>H03</td><td>h1</td><td>0.047367</td></tr>
<tr><td>C31</td><td>ca</td><td>-0.247000</td></tr>
<tr><td>H06</td><td>ha</td><td>0.139000</td></tr>
<tr><td>C32</td><td>ca</td><td>-0.061000</td></tr>
<tr><td>H07</td><td>ha</td><td>0.134000</td></tr>
<tr><td>C33</td><td>ca</td><td>-0.181000</td></tr>
<tr><td>H08</td><td>ha</td><td>0.143000</td></tr>
<tr><td>C34</td><td>ca</td><td>0.004800</td></tr>
<tr><td>N35</td><td>na</td><td>-0.148400</td></tr>
<tr><td>H14</td><td>hn</td><td>0.324700</td></tr>
<tr><td>C25</td><td>cd</td><td>-0.159300</td></tr>
<tr><td>C2</td><td>c</td><td>0.699300</td></tr>
<tr><td>O1</td><td>o</td><td>-0.637100</td></tr>
<tr><td>N3</td><td>n</td><td>-0.558900</td></tr>
<tr><td>H50</td><td>hn</td><td>0.327500</td></tr>
<tr><td>C4</td><td>c3</td><td>0.047700</td></tr>
<tr><td>C5</td><td>c3</td><td>-0.083400</td></tr>
<tr><td>C6</td><td>c3</td><td>-0.069700</td></tr>
<tr><td>C8</td><td>c3</td><td>-0.093600</td></tr>
<tr><td>H55</td><td>hc</td><td>0.038033</td></tr>
<tr><td>H56</td><td>hc</td><td>0.038033</td></tr>
<tr><td>H57</td><td>hc</td><td>0.038033</td></tr>
<tr><td>C7</td><td>c3</td><td>-0.093600</td></tr>
<tr><td>H58</td><td>hc</td><td>0.038033</td></tr>
<tr><td>H59</td><td>hc</td><td>0.038033</td></tr>
<tr><td>H60</td><td>hc</td><td>0.038033</td></tr>
<tr><td>H54</td><td>hc</td><td>0.047700</td></tr>
<tr><td>H52</td><td>hc</td><td>0.069200</td></tr>
<tr><td>H53</td><td>hc</td><td>0.069200</td></tr>
<tr><td>H51</td><td>h1</td><td>0.094700</td></tr>
<tr><td>C9</td><td>c</td><td>0.640100</td></tr>
<tr><td>O24</td><td>o</td><td>-0.620100</td></tr>
<tr><td>N10</td><td>n</td><td>-0.567900</td></tr>
<tr><td>H61</td><td>hn</td><td>0.348500</td></tr>
<tr><td>C11</td><td>c3</td><td>0.128900</td></tr>
<tr><td>C19</td><td>c2</td><td>-0.102200</td></tr>
<tr><td>C20</td><td>c2</td><td>-0.216100</td></tr>
<tr><td>H29</td><td>h4</td><td>0.208000</td></tr>
<tr><td>N21</td><td>no</td><td>0.275100</td></tr>
<tr><td>O22</td><td>o</td><td>-0.212000</td></tr>
<tr><td>O23</td><td>o</td><td>-0.212000</td></tr>
<tr><td>H71</td><td>ha</td><td>0.165000</td></tr>
</table>

H62	h1	0.089700
C12	c3	-0.080400
H63	hc	0.071700
H64	hc	0.071700
C13	c3	-0.140700
H65	hc	0.091700
C14	c3	-0.095400
H66	hc	0.060700
H67	hc	0.060700
C15	c3	0.092000
H68	h1	0.044200
H69	h1	0.044200
N16	n	-0.585900
H70	hn	0.335500
C17	c	0.697500
O18	o	-0.630500

ca-ca-os	69.600	119.200
ca-ca-ca	66.600	120.020
ca-ca-na	69.100	118.340
c3-os-ca	62.500	117.960
ca-ca-ha	48.200	119.880
h1-c3-os	50.800	109.780
h1-c3-h1	39.200	108.460
ca-na-hn	46.600	125.540
ca-na-cd	67.400	113.150
c -cd-na	68.700	123.270
cd-na-hn	46.800	125.500
cd-c -o	69.100	123.930
cd-c -n	69.100	112.700
c -n -hn	48.300	117.550
c -n -c3	63.400	120.690
n -c -o	74.200	123.050
c3-c3-n	65.900	111.610
h1-c3-n	49.800	108.880
c -c3-n	67.000	109.060
c3-n -hn	45.800	117.680
c3-c3-c3	62.900	111.510
c3-c3-hc	46.300	109.800
c3-c -o	67.400	123.200
c3-c -n	66.800	115.180
c3-c3-h1	46.400	109.560
c -c3-c3	63.300	111.040
hc-c3-hc	39.400	107.580
c -c3-h1	47.000	108.220
c2-c3-n	66.700	111.290
c2-c2-c3	64.100	123.630
c3-c2-ha	45.900	115.680
c2-c3-h1	47.100	109.960
c2-c3-c3	63.400	111.560
c2-c2-h4	49.400	122.670
c2-c2-no	67.400	123.460
c2-c2-ha	49.900	120.430
c2-no-o	69.400	117.670
h4-c2-no	49.300	113.380
o -no-o	76.700	125.080
c -c3-hc	46.900	108.770

Parameters

DIHEDRALS

cc-ca-ca-os	4	14.500	180.000	2.000
ca-ca-ca-cc	4	14.500	180.000	2.000
cc-ca-ca-na	4	14.500	180.000	2.000
o -c -cd-cc	4	11.500	180.000	2.000
n -c -cd-cc	4	11.500	180.000	2.000
ca-ca-cc-ha	4	2.800	180.000	2.000
ha-cc-cd-na	4	16.000	180.000	2.000
ha-cc-cd-c	4	16.000	180.000	2.000
ca-cc-cd-na	4	16.000	180.000	2.000
ca-cc-cd-c	4	16.000	180.000	2.000
ca-ca-os-c3	2	1.800	180.000	2.000
ca-ca-ca-ha	4	14.500	180.000	2.000
ca-ca-ca-ca	4	14.500	180.000	2.000
ca-ca-na-hn	4	1.200	180.000	2.000
ca-ca-na-cd	4	1.200	180.000	2.000
ca-ca-ca-na	4	14.500	180.000	2.000
h1-c3-os-ca	3	1.150	0.000	3.000
ha-ca-ca-os	4	14.500	180.000	2.000
ca-ca-ca-os	4	14.500	180.000	2.000
ha-ca-ca-ha	4	14.500	180.000	2.000
ha-ca-ca-na	4	14.500	180.000	2.000
cc-cd-na-ca	4	6.800	180.000	2.000
c -cd-na-ca	4	6.800	180.000	2.000
o -c -cd-na	4	11.500	180.000	2.000
n -c -cd-na	4	11.500	180.000	2.000

DIHEDRALS

c3-c3-c3-c3	1	0.200	180.000	1.000
c3-c3-c3-hc	1	0.160	0.000	3.000
c3-c -n -hn	4	10.000	180.000	2.000
c3-c -n -c3	1	0.000	0.000	-2.000
c3-c -n -c3	1	1.500	180.000	1.000
o -c -c3-c3	6	0.000	180.000	2.000
n -c -c3-c3	1	0.100	0.000	-4.000
n -c -c3-c3	1	0.070	0.000	2.000
hc-c3-c3-hc	1	0.150	0.000	3.000
c3-c3-c3-h1	9	1.400	0.000	3.000
h1-c3-c3-hc	9	1.400	0.000	3.000
o -c -c3-h1	1	0.800	0.000	-1.000
o -c -c3-h1	1	0.000	0.000	-2.000
o -c -c3-h1	1	0.080	180.000	3.000
n -c -c3-h1	6	0.000	180.000	2.000
c -c3-c3-c3	9	1.400	0.000	3.000
c -c3-c3-hc	9	1.400	0.000	3.000
c2-c3-n -c	6	0.000	0.000	2.000
c2-c2-c3-n	6	0.000	0.000	2.000
ha-c2-c3-n	6	0.000	0.000	2.000
c2-c3-n -hn	6	0.000	0.000	2.000
c3-c2-c2-h4	4	26.600	180.000	2.000
c3-c2-c2-no	4	26.600	180.000	2.000
c2-c3-c3-hc	9	1.400	0.000	3.000
c2-c3-c3-c3	9	1.400	0.000	3.000

cc-cd-na-hn 4 6.800 180.000 2.000	c2-c2-no-o 4 3.000 180.000 2.000
c -cd-na-hn 4 6.800 180.000 2.000	h4-c2-no-o 4 3.000 180.000 2.000
ca-ca-cc-cd 4 2.800 180.000 2.000	h4-c2-c2-ha 4 26.600 180.000 2.000
cd-c -n -hn 4 10.000 180.000 2.000	ha-c2-c2-no 4 26.600 180.000 2.000
cd-c -n -c3 4 10.000 180.000 2.000	c2-c2-c3-h1 6 0.000 0.000 2.000
c3-c3-n -c 1 0.500 180.000 -4.000	ha-c2-c3-h1 6 0.000 0.000 2.000
c3-c3-n -c 1 0.150 180.000 -3.000	c2-c2-c3-c3 6 0.000 0.000 2.000
c3-c3-n -c 1 0.000 0.000 -2.000	ha-c2-c3-c3 6 0.000 0.000 2.000
c3-c3-n -c 1 0.530 0.000 1.000	n -c -c3-hc 6 0.000 180.000 2.000
h1-c3-n -c 6 0.000 0.000 2.000	o -c -c3-hc 1 0.800 0.000 -1.000
c -c3-n -c 1 0.850 180.000 -2.000	o -c -c3-hc 1 0.000 0.000 -2.000
c -c3-n -c 1 0.800 0.000 1.000	o -c -c3-hc 1 0.080 180.000 3.000
o -c -n -hn 1 2.500 180.000 -2.000	
o -c -n -hn 1 2.000 0.000 1.000	**IMPROPER**
o -c -n -c3 4 10.000 180.000 2.000	ca-cd-cc-ha 1.1 180.0 2.0
c3-c3-c3-n 9 1.400 0.000 3.000	ca-ca-ca-cc 1.1 180.0 2.0
hc-c3-c3-n 9 1.400 0.000 3.000	ca-ca-ca-os 1.1 180.0 2.0
o -c -c3-n 6 0.000 180.000 2.000	ca-ca-ca-ha 1.1 180.0 2.0
n -c -c3-n 1 1.700 180.000 -1.000	ca-ca-ca-na 1.1 180.0 2.0
n -c -c3-n 1 2.000 180.000 2.000	ca-cd-na-hn 1.1 180.0 2.0
c3-c3-n -hn 6 0.000 0.000 2.000	c -cc-cd-na 1.1 180.0 2.0
h1-c3-n -hn 6 0.000 0.000 2.000	cd-n -c -o 10.5 180.0 2.0
c -c3-n -hn 6 0.000 0.000 2.000	c -c3-n -hn 1.1 180.0 2.0
c3-c3-c3-c3 1 0.180 0.000 -3.000	c3-n -c -o 10.5 180.0 2.0
c3-c3-c3-c3 1 0.250 180.000 -2.000	c2-c3-c2-ha 1.1 180.0 2.0
	c2-h4-c2-no 1.1 180.0 2.0

Table 5. Atom types, charges and parameters obtained for the inhibitor **FGA147** generated on the **E:I** covalent complex using antechamber package included in AmberTools. The partial charges were obtained by means of AM1-BCC calculations with the SQM program from the same suite.

Atom name	Atom type	Charge	Parameters
C32	ca	-0.133500	**NONBON**
C31	ca	-0.107500	ca 1.9080 0.0860
H16	ha	0.140000	ha 1.4590 0.0150
H17	ha	0.134000	c3 1.9080 0.1094
C30	ca	-0.120000	h1 1.3870 0.0157
H18	ha	0.133000	os 1.6837 0.1700
C29	ca	-0.133500	c 1.9080 0.0860
H19	ha	0.134000	o 1.6612 0.2100
C28	ca	-0.107500	n 1.8240 0.1700
H20	ha	0.140000	hn 0.6000 0.0157
C27	ca	-0.102300	hc 1.4870 0.0157
C26	c3	0.195700	c2 1.9080 0.0860
H21	h1	0.069700	no 1.8240 0.1700
H22	h1	0.069700	h4 1.4090 0.0150
O25	os	-0.433900	**BOND**
C2	c	0.743100	ca-ca 461.10 1.398
O1	o	-0.608000	ca-ha 345.80 1.086
N3	n	-0.551900	c3-ca 321.00 1.516
H24	hn	0.341500	c3-h1 330.60 1.097
C4	c3	0.057700	c3-os 308.60 1.432
C5	c3	-0.084400	c -os 390.80 1.358
C6	c3	-0.069700	c -o 637.70 1.218
C7	c3	-0.093600	c -n 427.60 1.379
H28	hc	0.038533	hn-n 403.20 1.013
H29	hc	0.038533	c3-n 328.70 1.462
H30	hc	0.038533	c3-c3 300.90 1.538
C8	c3	-0.093600	c -c3 313.00 1.524
H32	hc	0.038533	c3-hc 330.60 1.097
H33	hc	0.038533	c2-c3 326.80 1.510
H34	hc	0.038533	c2-c2 569.40 1.334
H35	hc	0.046700	c2-ha 343.10 1.088
H36	hc	0.072200	c2-no 343.00 1.448
H37	hc	0.072200	c2-h4 344.60 1.087

H38	h1	0.092700
C9	c	0.640100
O24	o	-0.617100
N10	n	-0.567900
H40	hn	0.348500
C11	c3	0.127900
C19	c2	-0.101200
C20	c2	-0.216100
N21	no	0.275100
O23	o	-0.211000
O22	o	-0.211000
H61	h4	0.206000
H62	ha	0.166000
H56	h1	0.091700
C12	c3	-0.080400
H54	hc	0.072200
H55	hc	0.072200
C13	c3	-0.141700
H53	hc	0.091700
C14	c3	-0.095400
H51	hc	0.060700
H52	hc	0.060700
C15	c3	0.092000
H49	h1	0.044700
H50	h1	0.044700
N16	n	-0.585900
H48	hn	0.335500
C17	c	0.697500
O18	o	-0.630500

no-o 741.80 1.226

ANGLE

ca-ca-ha 48.200 119.880
ca-ca-ca 66.600 120.020
c3-ca-ca 63.500 120.770
ca-c3-h1 47.000 109.560
ca-c3-os 68.300 108.950
c -os-c3 63.300 115.980
h1-c3-h1 39.200 108.460
h1-c3-os 50.800 109.780
o -c -os 75.300 123.250
n -c -os 75.300 109.220
c -n -hn 48.300 117.550
c -n -c3 63.400 120.690
n -c -o 74.200 123.050
c3-c3-n 65.900 111.610
h1-c3-n 49.800 108.880
c -c3-n 67.000 109.060
c3-n -hn 45.800 117.680
c3-c3-c3 62.900 111.510
c3-c3-hc 46.300 109.800
c3-c -o 67.400 123.200
c3-c -n 66.800 115.180
c3-c3-h1 46.400 109.560
c -c3-c3 63.300 111.040
hc-c3-hc 39.400 107.580
c -c3-h1 47.000 108.220
c2-c3-n 66.700 111.290
c2-c2-c3 64.100 123.630
c3-c2-ha 45.900 115.680
c2-c3-h1 47.100 109.960
c2-c3-c3 63.400 111.560
c2-c2-no 67.400 123.460
c2-c2-h4 49.400 122.670
c2-c2-ha 49.900 120.430
c2-no-o 69.400 117.670
h4-c2-no 49.300 113.380
o -no-o 76.700 125.080
c -c3-hc 46.900 108.770

Parameters

DIHEDRALS

ca-ca-ca-ca 4 14.500 180.000 2.000
c3-ca-ca-ca 4 14.500 180.000 2.000
ca-ca-ca-ha 4 14.500 180.000 2.000
h1-c3-ca-ca 6 0.000 0.000 2.000
os-c3-ca-ca 6 0.000 0.000 2.000
c3-ca-ca-ha 4 14.500 180.000 2.000
ha-ca-ca-ha 4 14.500 180.000 2.000
ca-c3-os-c 3 1.150 0.000 3.000
o -c -os-c3 1 2.700 180.000 -2.000
o -c -os-c3 1 1.400 180.000 1.000
n -c -os-c3 2 5.400 180.000 2.000
h1-c3-os-c 3 1.150 0.000 3.000
os-c -n -hn 4 10.000 180.000 2.000
os-c -n -c3 4 10.000 180.000 2.000
c3-c3-n -c 1 0.500 180.000 -4.000
c3-c3-n -c 1 0.150 180.000 -3.000
c3-c3-n -c 1 0.000 0.000 -2.000
c3-c3-n -c 1 0.530 0.000 1.000
h1-c3-n -c 6 0.000 0.000 2.000
c -c3-n -c 1 0.850 180.000 -2.000
c -c3-n -c 1 0.800 0.000 1.000

DIHEDRALS

o -c -c3-c3 6 0.000 180.000 2.000
n -c -c3-c3 1 0.100 0.000 -4.000
n -c -c3-c3 1 0.070 0.000 2.000
hc-c3-c3-hc 1 0.150 0.000 3.000
c3-c3-c3-h1 9 1.400 0.000 3.000
h1-c3-c3-hc 9 1.400 0.000 3.000
o -c -c3-h1 1 0.800 0.000 -1.000
o -c -c3-h1 1 0.000 0.000 -2.000
o -c -c3-h1 1 0.080 180.000 3.000
n -c -c3-h1 6 0.000 180.000 2.000
c -c3-c3-c3 9 1.400 0.000 3.000
c -c3-c3-hc 9 1.400 0.000 3.000
c2-c3-n -c 6 0.000 0.000 2.000
c2-c2-c3-n 6 0.000 0.000 2.000
ha-c2-c3-n 6 0.000 0.000 2.000
c2-c3-n -hn 6 0.000 0.000 2.000
c3-c2-c2-no 4 26.600 180.000 2.000
c3-c2-c2-h4 4 26.600 180.000 2.000
c2-c3-c3-hc 9 1.400 0.000 3.000
c2-c3-c3-c3 9 1.400 0.000 3.000
c2-c2-no-o 4 3.000 180.000 2.000

o -c -n -hn 1 2.500 180.000 -2.000	h4-c2-no-o 4 3.000 180.000 2.000
o -c -n -hn 1 2.000 0.000 1.000	ha-c2-c2-no 4 26.600 180.000 2.000
o -c -n -c3 4 10.000 180.000 2.000	h4-c2-c2-ha 4 26.600 180.000 2.000
c3-c3-c3-n 9 1.400 0.000 3.000	c2-c2-c3-h1 6 0.000 0.000 2.000
hc-c3-c3-n 9 1.400 0.000 3.000	ha-c2-c3-h1 6 0.000 0.000 2.000
o -c -c3-n 6 0.000 180.000 2.000	c2-c2-c3-c3 6 0.000 0.000 2.000
n -c -c3-n 1 1.700 180.000 -1.000	ha-c2-c3-c3 6 0.000 0.000 2.000
n -c -c3-n 1 2.000 180.000 2.000	n -c -c3-hc 6 0.000 180.000 2.000
c3-c3-n -hn 6 0.000 0.000 2.000	o -c -c3-hc 1 0.800 0.000 -1.000
h1-c3-n -hn 6 0.000 0.000 2.000	o -c -c3-hc 1 0.000 0.000 -2.000
c -c3-n -hn 6 0.000 0.000 2.000	o -c -c3-hc 1 0.080 180.000 3.000
c3-c3-c3-c3 1 0.180 0.000 -3.000	**IMPROPER**
c3-c3-c3-c3 1 0.250 180.000 -2.000	ca-ca-ca-ha 1.1 180.0 2.0
c3-c3-c3-c3 1 0.200 180.000 1.000	c3-ca-ca-ca 1.1 180.0 2.0
c3-c3-c3-hc 1 0.160 0.000 3.000	n -o -c -os 1.1 180.0 2.0
c3-c -n -hn 4 10.000 180.000 2.000	c -c3-n -hn 1.1 180.0 2.0
c3-c -n -c3 1 0.000 0.000 -2.000	c3-n -c -o 10.5 180.0 2.0
c3-c -n -c3 1 1.500 180.000 1.000	c2-c3-c2-ha 1.1 180.0 2.0
	c2-h4-c2-no 1.1 180.0 2.0

Table 6. Predicted pK_a values of all titratable residues as computed with PropKa ver 3.0.3. Each residue is identified by its name, residue number and chain. Histidine residues have been labeled according to their protonation states: HID for a protonated N_δ atom, or HIE for a N_ε one.

Residue	pK_a	Residue	pK_a	Residue	pK_a	Residue	pK_a
ASP-33-A	4.3	GLU-270-A	4.0	CYS-38-B	13.0	LYS-137-A	10.1
ASP-34-A	3.7	GLU-288-A	5.1	CYS-44-B	11.4	LYS-236-A	10.6
ASP-48-A	3.5	GLU-290-A	4.2	CYS-85-B	11.9	LYS-269-A	10.0
ASP-56-A	3.8	GLU-14-B	6.6	CYS-117-B	13.0	LYS-5-B	10.7
ASP-92-A	3.9	GLU-47-B	4.7	CYS-128-B	12.1	LYS-12-B	11.0
ASP-153-A	4.4	GLU-55-B	4.0	CYS-145-B	14.9	LYS-61-B	10.5
ASP-155-A	3.7	GLU-166-B	1.9	CYS-156-B	9.7	LYS-88-B	10.0
ASP-176-A	3.7	GLU-178-B	4.9	CYS-160-B	13.0	LYS-90-B	10.6
ASP-187-A	3.7	GLU-240-B	6.7	CYS-265-B	12.1	LYS-97-B	10.3
ASP-197-A	3.5	GLU-270-B	4.1	CYS-300-B	10.9	LYS-100-B	10.5
ASP-216-A	3.9	GLU-288-B	5.4	TYR-37-A	12.2	LYS-102-B	11.0
ASP-229-A	3.5	GLU-290-B	1.9	TYR-54-A	15.3	LYS-137-B	9.8
ASP-245-A	4.0	HID-41-A	3.9	TYR-101-A	11.1	LYS-236-B	10.5
ASP-248-A	3.5	HIE-64-A	6.3	TYR-118-A	12.2	LYS-269-B	10.4
ASP-263-A	3.4	HID-80-A	5.7	TYR-126-A	14.7	ARG-4-A	13.1
ASP-289-A	2.8	HIE-163-A	1.4	TYR-154-A	10.1	ARG-40-A	14.2
ASP-295-A	5.2	HIE-164-A	1.9	TYR-161-A	15.6	ARG-60-A	12.6
ASP-33-B	3.9	HIE-172-A	4.6	TYR-182-A	13.5	ARG-76-A	12.5
ASP-34-B	3.6	HIE-246-A	5.4	TYR-209-A	12.7	ARG-105-A	13.1
ASP-48-B	3.1	HID-41-B	3.7	TYR-237-A	10.1	ARG-131-A	16.0
ASP-56-B	4.1	HIE-64-B	6.3	TYR-239-A	12.6	ARG-188-A	12.3
ASP-92-B	2.5	HID-80-B	5.7	TYR-37-B	11.7	ARG-217-A	12.2
ASP-153-B	3.7	HIE-163-B	2.2	TYR-54-B	15.0	ARG-222-A	12.5
ASP-155-B	3.0	HIE-164-B	1.5	TYR-101-B	12.3	ARG-279-A	12.1
ASP-176-B	3.8	HIE-172-B	5.3	TYR-118-B	12.2	ARG-298-A	11.4

ASP-187-B	4.1	HIE-246-B	4.8	TYR-126-B	14.9	ARG-4-B	13.3
ASP-197-B	3.8	CYS-16-A	12.0	TYR-154-B	10.5	ARG-40-B	14.5
ASP-216-B	3.6	CYS-22-A	9.9	TYR-161-B	15.6	ARG-60-B	12.5
ASP-229-B	3.7	CYS-38-A	12.8	TYR-182-B	13.5	ARG-76-B	13.3
ASP-245-B	3.9	CYS-44-A	11.5	TYR-209-B	12.4	ARG-105-B	13.1
ASP-248-B	4.2	CYS-85-A	12.2	TYR-237-B	10.3	ARG-131-B	15.9
ASP-263-B	3.5	CYS-117-A	13.1	TYR-239-B	12.6	ARG-188-B	12.4
ASP-289-B	3.5	CYS-128-A	11.4	LYS-5-A	9.5	ARG-217-B	12.4
ASP-295-B	4.9	CYS-145-A	14.9	LYS-12-A	10.3	ARG-222-B	12.5
GLU-14-A	5.6	CYS-156-A	9.5	LYS-61-A	11.0	ARG-279-B	12.2
GLU-47-A	4.7	CYS-160-A	13.0	LYS-88-A	9.9	ARG-298-B	10.4
GLU-55-A	4.7	CYS-265-A	12.2	LYS-90-A	10.5	NTerminal-A	7.8
GLU-166-A	1.1	CYS-300-A	11.5	LYS-97-A	10.1	NTerminal-B	6.5
GLU-178-A	4.9	CYS-16-B	12.0	LYS-100-A	10.8		
GLU-240-A	6.0	CYS-22-B	11.0	LYS-102-A	10.7		

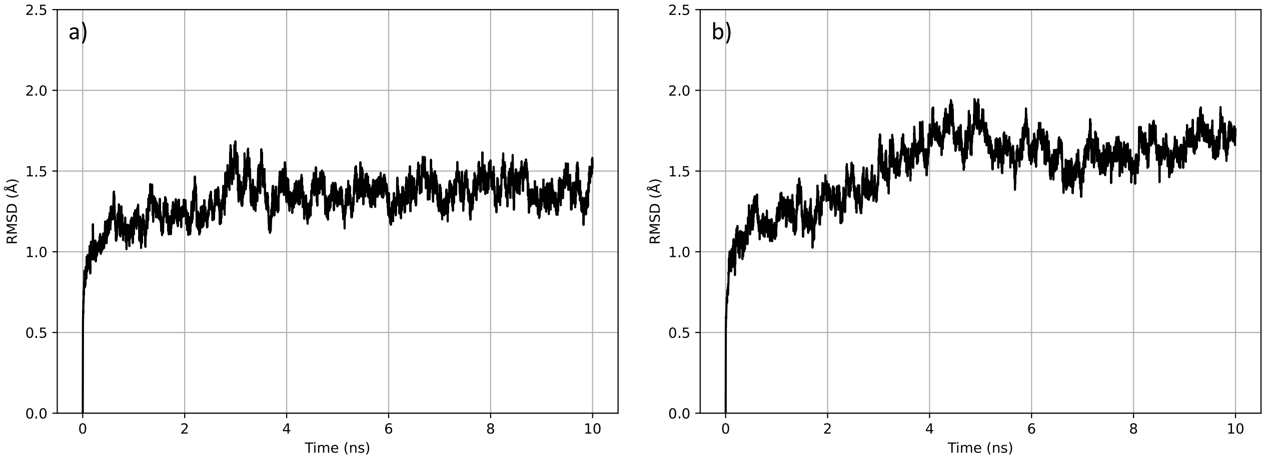

Figure 10. RMSD along the classical 10 ns MD simulation for the backbone atoms of the SARS-CoV-2 M^{pro} cysteine protease. Simulations performed on the non-covalent reactant state corresponding to the E:I reactant complex. a) **FGA146**. b) **FGA147**.

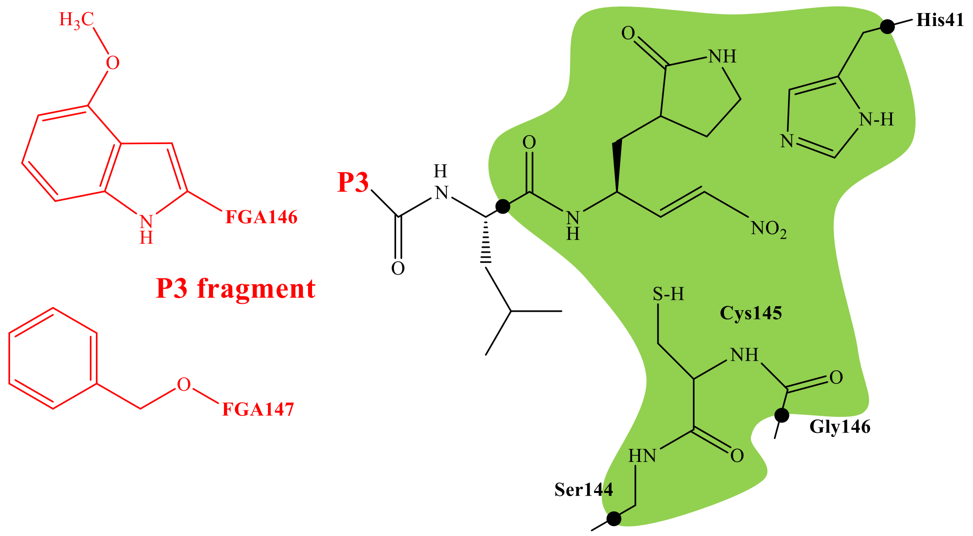

Figure 11. Details of the atoms included in the QM region (green region) in QM/MM calculations on both **FGA146** and **FGA147** inhibitors. The black dots represent the hydrogen link atoms.

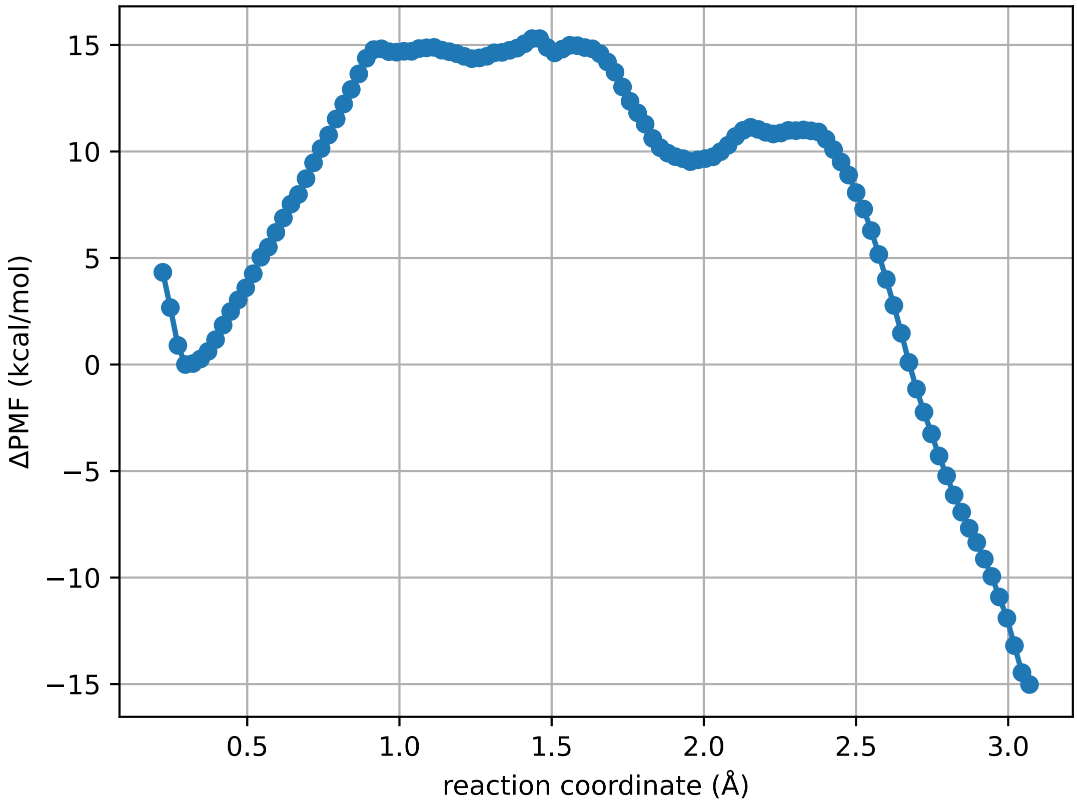

Figure 12. M06-2X/6-31+G(d,p)/MM FESs obtained by means of umbrella sampling MD method corresponding to the inhibition of SARS-CoV-2 M^{pro} by **FGA146**.

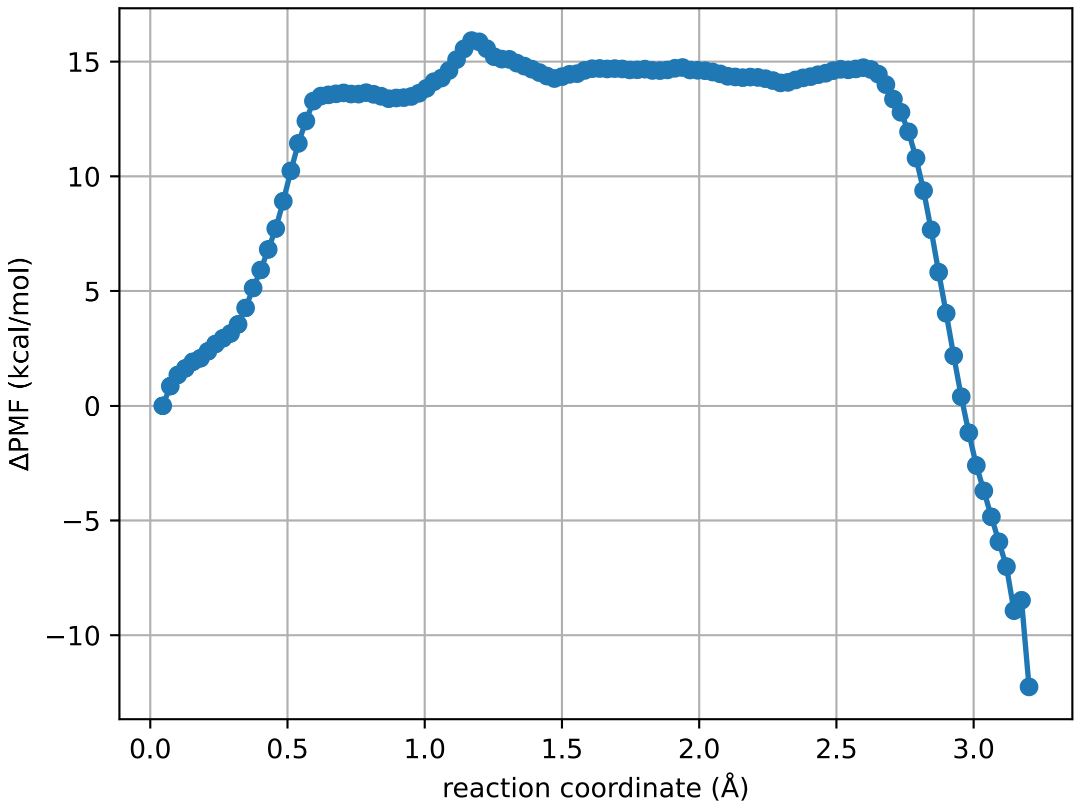

Figure 13. M06-2X/6-31+G(d,p)/MM FESs obtained by means of umbrella sampling MD method corresponding to the inhibition of SARS-CoV-2 M^{pro} by **FGA147**.

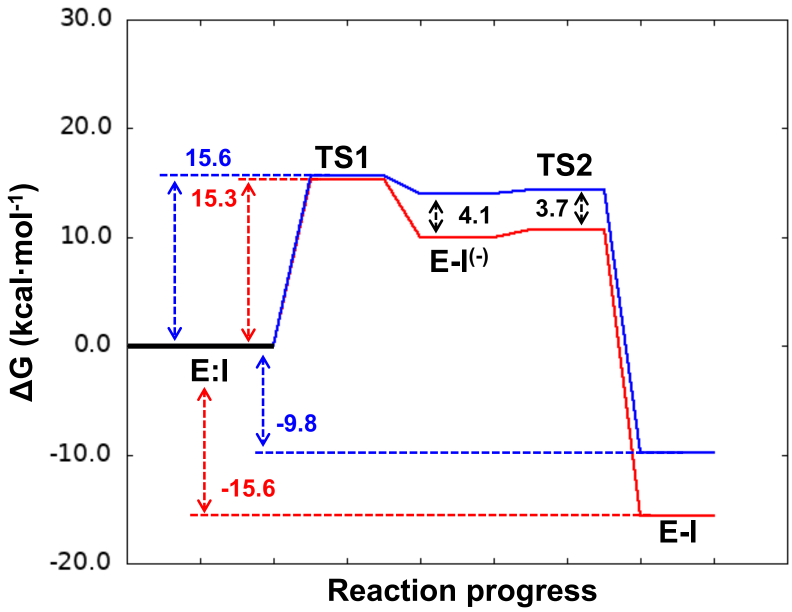

Figure 14. M06-2X/6-31+G(d,p)/MM free energy profiles for covalent complex formation between SARS-CoV-2 Mpro and: FGA146 (red line); and FGA147 (blue line) compounds, derived from data reported in Figures S12 and S13. Energies are in kcal·mol^{-1}.

Computational study of the SARS-CoV-2 M^{pro} inhibition by FGA146 and FGA147. The measured K_i values for both inhibitors, which are around 1-2 µM (see Table 1), would correspond to differences of energy of ca. -14 kcal·mol^{-1}, which is close to the reaction energy of **FGA146** but much larger than the value obtained with **FGA147**. However, it must be taken into account that the K_i values of Table 1 corresponds to the equilibrium between the product covalent complex (**E-I**) and the solvent separated reactant species (**E + I**), while our simulations are not including the binding step to form the reactant non-covalent complex (**E + I** to **E:I** step). Thus, apart from the intrinsic uncertainty associated to the computational methods, it is possible that the binding of **FGA147** into the enzyme active site was more exergonic than the binding of **FGA146**, thus compensating at some extent the differences. Structures of the different states appearing along the reaction optimized at M06-2X/MM level are shown in Figure 4, while a list of key inter-atomic distances obtained on the representative stable states is listed in Table S7 and S8.

Table 7. Key inter-atomic distances (in Å) for the keystates located along the inhibition mechanism of SARS-CoV-2 M^{pro} by the inhibitor **FGA146,** optimized at M06-2X/6-31+G(d,p)/MM level of theory.

**Distances**	**E:I**	**TS1**	**E-I$^{(-)}$**	**TS2**	**E-I**
**Active Site**					
Sγ^{C145}- C19	3.35	2.81	1.97	1.92	1.89
C19 - C20	1.34	1.34	1.44	1.52	1.46
Sγ^{C145}- Hγ^{C145}	1.36	1.59	3.23	2.74	2.93
Nε^{H41}- Hγ^{C145}	2.00	1.31	1.01	1.32	2.54
C20 - Hγ^{C145}	3.55	3.01	3.22	1.42	1.08
**Oxyanion hole**					
O22 - H^{G143}	3.07	3.66	3.90	3.21	3.33
O22 – H^{S144}	4.86	5.02	5.86	5.15	5.15
O22 - H^{C145}	4.24	3.89	4.22	4.02	4.00
**P1´-S1´**					
O23- Nε^{H41}	3.69	3.60	3.37	3.64	3.28
**P1-S1**					
O18 - HE2^{H163}	1.86	2.33	2.25	2.11	2.55
H^{N16H} - O^{F140}	1.90	2.04	2.01	2.22	2.09
H^{N16H} - OE1^{E166}	5.22	5.17	4.80	4.37	4.94
H^{N10H} - O^{H164}	2.61	2.40	2.06	2.18	1.84
**P2-S2**					
H^{N3H} - OE1^{Q189}	2.30	2.16	1.86	2.13	1.98
**P3-S3**					
O1 - H^{E166}	1.80	1.93	1.92	1.84	1.98
H^{N35H} - O^{E166}	1.82	2.01	1.81	1.74	1.74

Table 8. Key inter-atomic distances (in Å) for the key states located along the inhibition mechanism of SARS-CoV-2 M^{pro} by the inhibitor **FGA147,** optimized at M06-2X/6-31+G(d,p)/MM level of theory.

Distances	E:I	TS1	$E\text{-}I^{(-)}$	TS2	E-I
**Active Site**					
$S\gamma^{C145}$- C19	3.04	2.63	1.89	1.86	1.84
C19 - C20	1.33	1.35	1.49	1.53	1.53
$S\gamma^{C145}$- $H\gamma^{C145}$	1.37	1.77	2.95	2.90	2.75
$N\varepsilon^{H41}$- $H\gamma^{C145}$	1.92	1.29	1.06	1.26	1.99
C20 - $H\gamma^{C145}$	3.60	3.15	2.03	1.62	1.11
**Oxyanion hole**					
O22 - H^{G143}	2.99	3.15	3.26	3.25	3.20
O22 – H^{S144}	3.96	4.10	4.23	4.24	4.20
O22 - H^{C145}	2.77	2.83	2.92	2.94	2.95
**P1´-S1´**					
O23- $N\varepsilon^{H41}$	5.35	4.59	3.91	3.89	4.00
**P1-S1**					
O18 - $HE2^{H163}$	2.42	2.36	2.31	2.31	2.30
H^{N16H} - O^{F140}	3.25	3.29	3.34	3.34	3.35
H^{N16H} - $OE1^{E166}$	2.92	2.97	3.05	3.05	3.07
H^{N10H} - O^{H164}	2.38	2.17	2.02	2.02	2.00
**P2-S2**					
H^{N3H} - $OE1^{Q189}$	2.11	2.11	2.11	2.11	2.11
**P3-S3**					
O1 - H^{E166}	2.07	2.07	2.07	2.07	2.07

The estimation of the main interaction energies between residues of SARS-CoV-2 M^{pro} and the inhibitors **FGA146** and **FGA147** computed in the **E:I** and in the **E-I** states are shown in Figure S15. It is important to point out that, due to the nature of the computed interaction (electrostatic plus Lennard−Jones) some of the interactions do not necessary imply close distance protein-inhibitor contacts. Consequently, these energetic results can complement structural analysis derived from the crystallographic studies. Our results show how the pattern of interactions does not dramatically change from the Michaelis complex **E:I** to the final **E-I** covalent product complex, in both reactions. Moreover, as reflected by the similar plots obtained for **FGA146** and **FGA147**, the influence of the P3, the only fragment that distinguishes the two inhibitors, does not dramatically affect the rest of the protein-inhibitor interactions, despite it has an effect in the thermodynamics of the process, more exergonic in the case of **FGA147** than in **FGA146**, which is dictated not only by the QM-MM interactions but by the QM and MM terms. The interaction between the P3 moiety of **FGA146** with Glu166 and Gln189 residues are not observed in the case of **FGA147**, where only a weak interaction has been measured with Met165 (see Figures S18 and S15). This is due to the presence of the methoxy and the indole ring in the former that facilitates direct H-bond interactions with polar residues of the cavity. These differences are related with the different orientation of the methoxy indole ring of **FGA146** and the Cbz of **FGA147** in the S3 cavity, as discussed below. On the other side, in both cases the interactions

with S1′ take place mainly through hydrogen bond interactions with the nitro oxygen atoms of the P1 that is common in both inhibitors. However, while the nitro group of **FGA147** is interacting with the oxyanion hole located in S1′ formed by Gly143, Ser144, and Cys145, this interaction is partially lost and an additional interaction with His41 is observed in the case of **FGA146** (see Figure S15). In addition, some indirect interactions stabilize the P1′ fragment, such as Leu27, Asn28, Pro39, Gly146, and Ser147, in **FGA146**, and Thr25, Asn28, Gly146, and Ser147 in **FGA147**. The specific favorable interactions between the lactam ring on P1 and S1 match in both inhibitors through interactions with F140, Asn142 and His163. The interaction with His172 is exclusive of the **FGA146** while the interaction with Glu166 is only observed in **FGA147**. His164 and Asp187 interact with P2 in both cases, while in the case of **FGA146** an additional interaction with Met165 is detected together with a weak interaction with Asp176. Finally, there are unfavorable interactions such as those between Arg40 and the warhead of both inhibitors, between Arg188 and P2 of **FGA146**, and between Ser1 of chain B and P1 in **FGA147**. Interestingly, in both complexes, Arg40 is ca. 910 Å from P1′ while Arg188 is ca. 57 Å, thus corresponding to electrostatic interactions. Interestingly, the significant unfavorable interactions with Arg40 were already detected when studying the inhibition of M^{pro} with other designed inhibitors[47].

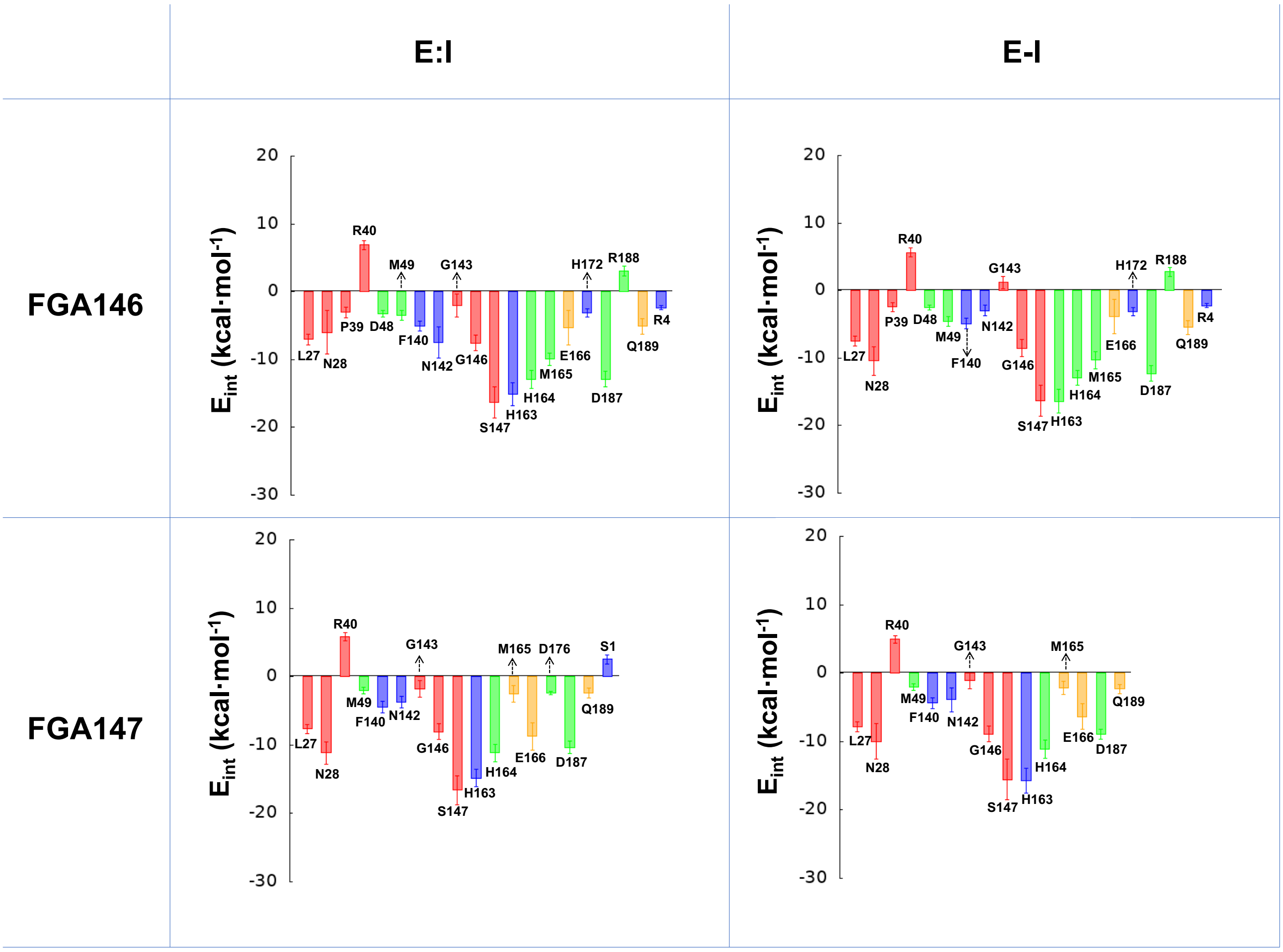

Figure 15. Main average interaction energies (electrostatic plus Lennard-Jones) between residues of SARS-CoV-2 M^{pro} and the inhibitors FGA146 or FGA147, computed in the E:I and the E-I states. The colour of the bars indicate the specific interactions: red, blue, green and orange correspond to P1'::S1', P1:::S1, P2:::S2 and P3:::S3 interactions, respectively. Results obtained as an average over 1000 structures of the AM1/MM MD simulations. Only those interactions larger than 2 kcal·mol^{-1} are reported.

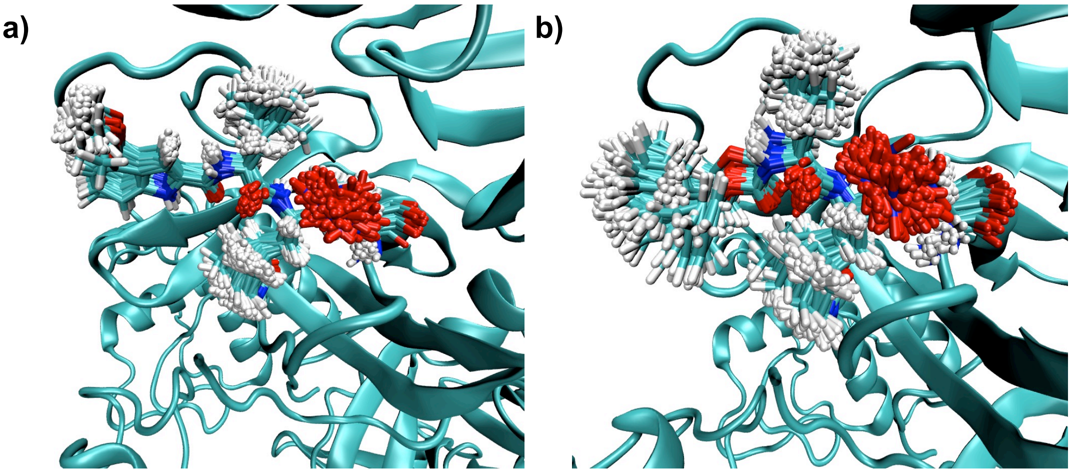

Figure 16. Overlap of structures generated during 100 ns of MM MD simulation of the (a) FGA146 and (b) FGA147 inhibitors, covalently bounded to the active site of SARS-CoV-2 M^{pro} through Cys145. Structures of the inhibitors randomly selected every 100 frames for clarity purposes**.**

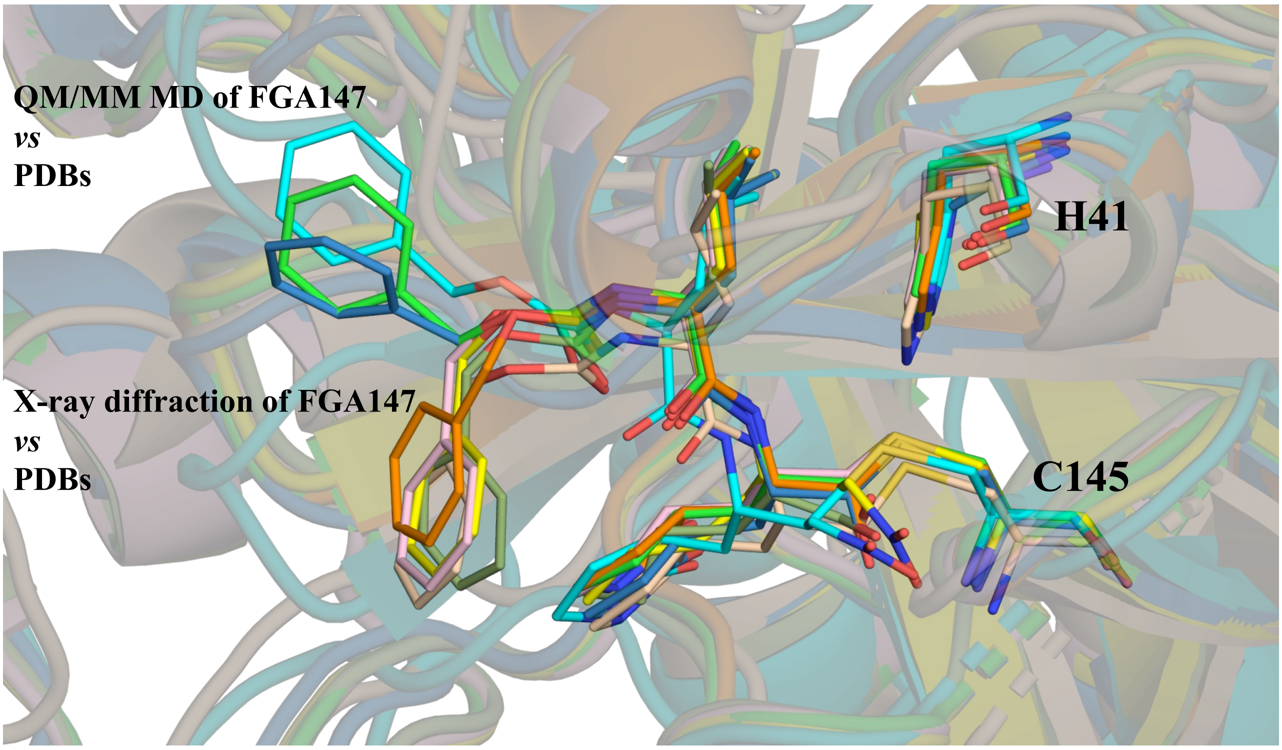

Figure 17. Comparison of QM/MM MD (in cyan) and X-ray structures (in yellow) of covalent complex SARS CoV2-M^{pro}·FGA147. The rest of structures are from previously reported crystallographic structures of SARS CoV2-M^{pro} in complex with other peptidomimetic covalent inhibitors with the same recognition part as **FGA147**. The green (PDB ID 7UKK), orange (PDB ID 7UJG), pink (PDB ID 7TOB), and wheat colors (PDB ID 7SMV) corresponds with the SARS CoV2-M^{pro} complexed with the inhibitor called GC-376. The dark blue color (PDB ID 7UKK) corresponds with the SARS CoV2-M^{pro} complexed with the inhibitor called GC-373. The lime color (PDB ID 7AKU) corresponds with the SARS CoV2-M^{pro} complexed with the inhibitor called Calpeptin.

Comparison of computational and crystallographic structures.

The X-ray structures confirm the predictions based on the QM/MM simulations. As shown in Figure S16 and S18, the comparison of structures derived from M06-2X/MM optimizations with the corresponding complexes derived from experiments confirm a good agreement, both techniques virtually describing the same binding mode of the most active compounds in the active site of M^{pro}. A detailed analysis of the active site of the SARS-CoV-2 M^{pro} in complex with inhibitors **FGA146** and **FGA147** can be derived from Figures 2-5 and Figures S5-S6, together with interatomic distances between protein atoms and inhibitor atoms that are summarized in Table S3 and S7. It is important to stress that the computer simulations were initiated from a previous solved X-ray structure of SARS-CoV-2 M^{pro} complexed with the N3 inhibitor (PDB ID 6LU7)[48].

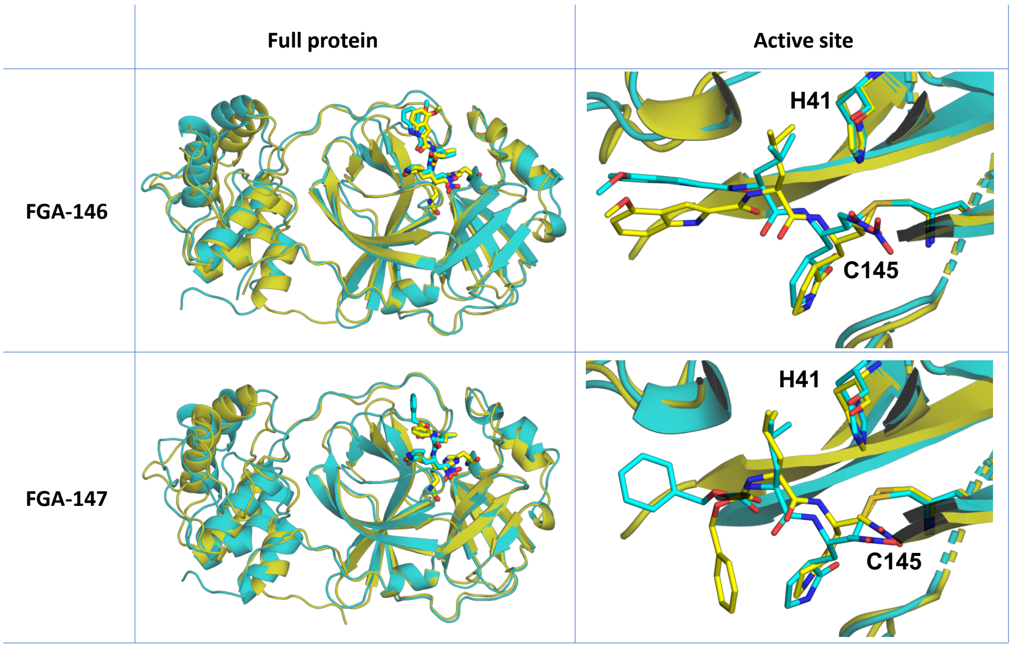

Figure 18. Detail of the FGA146 and FGA147 inhibitors covalently bounded to the active site of SARS-CoV-2 M^{pro} through Cys145. Results derived from X-ray diffraction (in yellow) and M06-2X/MM optimizations (in cyan).

The computational findings of **E-I** product complex of the inhibition with **FGA146** fit with the presence of two different conformations in monomer A and B in the crystal structure. Thus, the nitro group can slightly move away from the "oxyanion hole" and approaching to His41 during the MD simulations, mirroring the crystallographic data of monomer A (Table S7 and Fig. A19 vs Table S3 and Fig. S7, brown). In the case of **FGA147**, the nitro group appears to interact basically with the oxyanion hole in both, simulations and X-ray diffractions. Regarding the **FGA147**, an *a priori* discrepancy between experiments and theory is found regarding the P3 residue side chain of the Cbz group. Thus, the QM/MM MD simulations suggest the P3 residue is oriented, on average, towards the S4 subsite (Figure S18 and S19). This most populated orientation of P3 in **FGA147** is stabilized by an interaction between the phenyl group of Cbz and the Gln189 and Glu166

residues, which is confirmed by the computed favorable interaction (Figure S15). On the contrary, the X-ray structure of M^{pro} complex with **FGA147** shows that side chain of this residue is oriented towards the solvent not having any interactions with protein residues (Figures 2 and S18). In this case, the position of the Cbz group in **FGA147** is forced by a symmetry-related molecule into the crystal, located just above the active site and thus preventing the inhibitor to adopt a conformation that matches the species suggested by computer simulations. However, when considering the conformational space explored with just a short MD simulation with classical force fields (Figure S16b), orientations of P3 similar to the one determined by means of X-ray diffraction were detected. In the case of **FGA146**, the orientations observed for the 4-methoxy-1H-indole-2-carbonyl group appear to be more constrained (Figure S16a). Interestingly, the two possible orientations of P3 in **FGA147** have been detected in previously solved structures of SARS CoV-2 M^{pro} in complex with peptidomimetic covalent inhibitors presenting the same recognition part as **FGA147** (see Figure S17).[49-53] Concerning the rest of the protein (Figure S19), there is a general good agreement between experiments and simulations, except for the very flexible regions exposed to the solvent (the C-terminal loop, S301-G302-V303-T304-F305-Q306 residues). These results confirm the robustness of the results and the low impact of the ligand on the full structure of the protein.

Regarding the kinetics, this is dictated by the first chemical step, with virtually the same activation free energy barrier computed with both inhibitors (15.3 and 15.6 kcal·mol^{-1} with **FGA146** and **FGA147**, respectively). The results suggest that the inhibitory activity of the inhibitors are dictated by both, the favorable protein:inhibitors binding and the chemical steps of the inhibition process. QM-MM averaged interaction energies (electrostatic plus Lennard–Jones) between residues of SARS-CoV-2 M^{pro} and the inhibitors **FGA146** and **FGA147** allows complementing the geometrical analysis based just on short distance (H-bond) interactions. The similar plots obtained for **FGA146** and **FGA147**, indicate that the influence of the P3, that is the only fragment that differentiate them, does not dramatically affect the rest of the protein-inhibitor interactions, but the energy of the **E-I** covalent complex, relative to the solvent separated species, **E + I**.

Physicochemical parameters. Physicochemical parameters were calculated using molinspiration (Molinspiration property engine v2022.08, https://www.molinspiration.com/cgi-bin/properties).

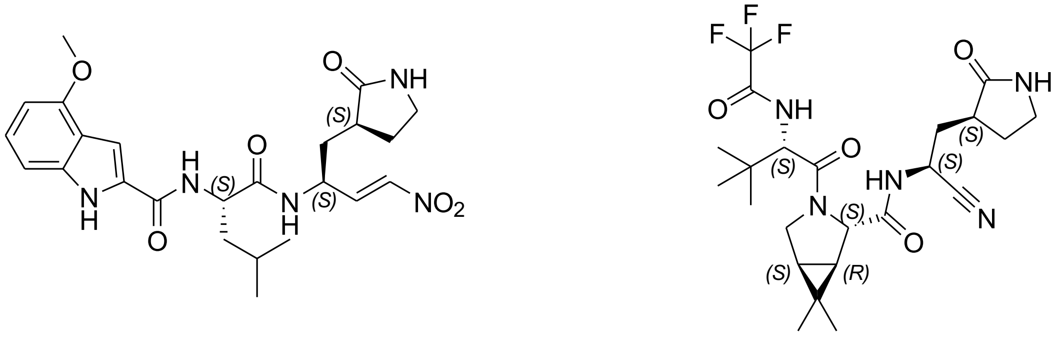

Figure 19. The two compounds for which predicted physicochemical parameters were compared.

Table 9. Comparison of predicted physicochemical parameters for FGA146 and Nirmatrelvir.

Parameter	Predicted values FGA146	Predicted values Nirmatrelvir
milogP	2.17	1.66
TPSA	158.14	131.40
M	458.54	499.53
N(Atoms)	35	35
N(H-Acc)	11	9
N(H-Don)	4	3
N(rot. Bonds)	11	8
Volume	440.27 Å^3	438.18 Å^3
N(Ro5 violations)	1 (too many H-acceptors)	0

Cysteine reactivity. The assay was performed after Lit.[54] with 10 µM probe and cysteine concentrations, respectively. PBS (DPBS Sigma D5652; pH 7.4) was purged with argon and then used as the buffer. Cysteine solutions were prepared freshly and used immediately. The two replicate measurements were recorded on two different Tecan instruments (infinite F200 pro and Spark 10M), which explains the different absolute values for fluorescence.
The reactivity of FGA146 towards free nucleophiles (cysteine) was assessed. First, to gauge reactivity beyond general stability in solution (reaction with organic nucleophiles), second, to assess selectivity for non-active-site cysteine.

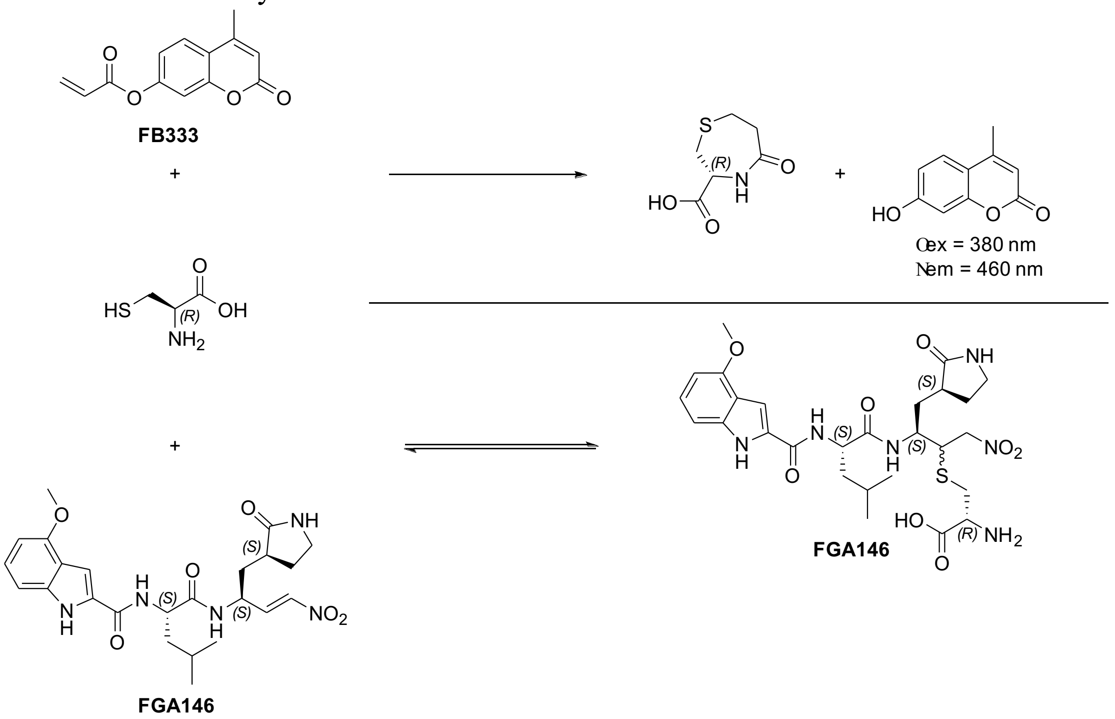

Figure 20. Schematic representation of the competing reactions during the cysteine assay.

The negative control (0 µM) portrays the reaction progress of cysteine with the cysteine-reactive probe FB333 (both 10 µM). With increasing FGA146 concentrations, the reaction of the reporter system is markedly suppressed. For concentrations in the same range as reporter concentrations (6–12.5 µM), the reaction still takes place, but at a lower rate. With excess of nitroalkene (>2.5x concentration), the reporter reaction does not progress measurably.
It is apparent that the reactivity of FGA146 toward free nucleophiles is noteworthy, especially in the context of cellular, GSH-based detoxification mechanisms (concentrations in humans can be µM (plasma) to single-digit mM (intracellular))[55].

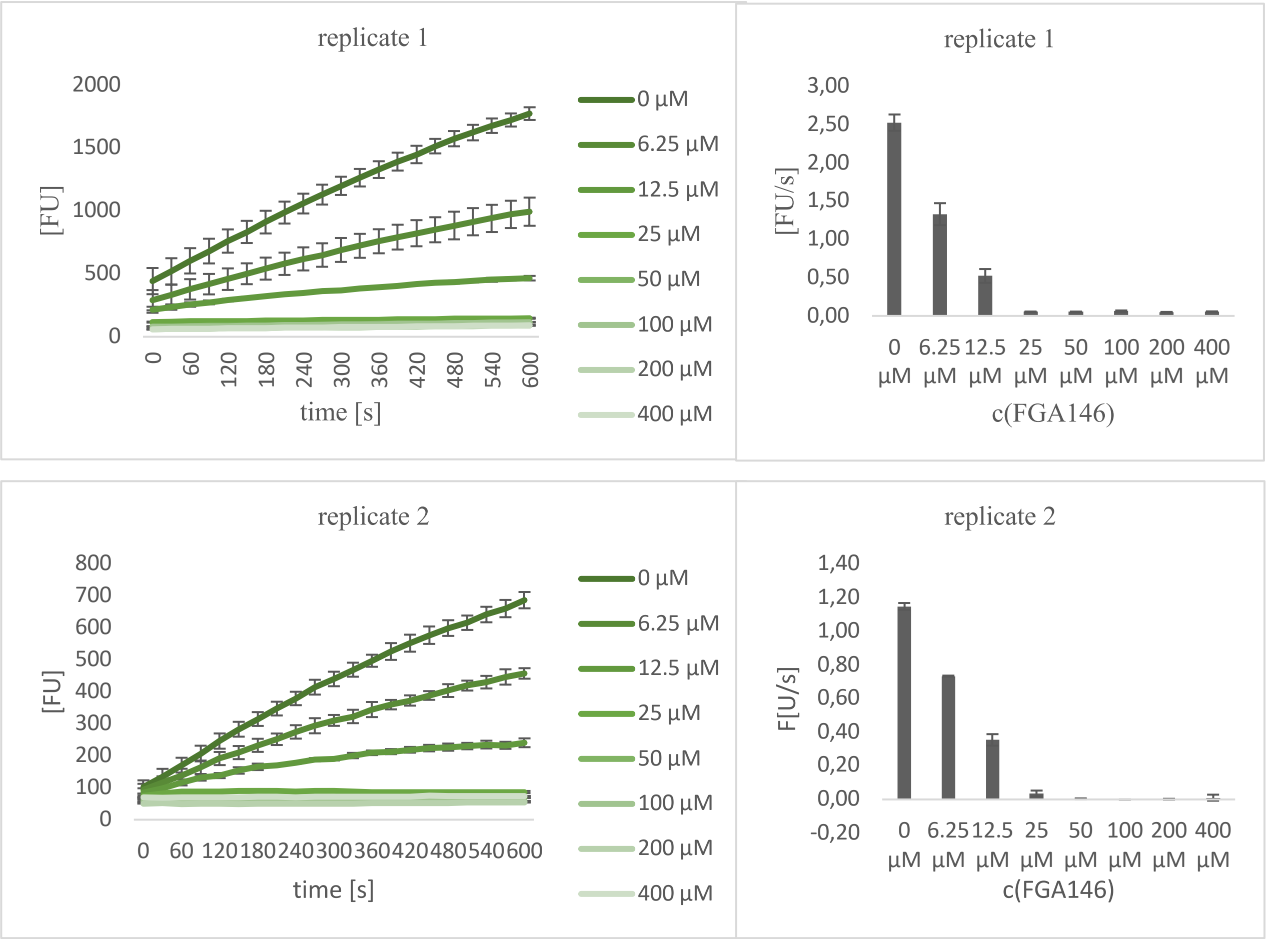

Figure 21. Left F-t-diagrams for reporter reaction in presence of increasing concentrations of FGA146. Right Initial slopes of reporter reaction data in presence of increasing concentrations of FGA146.

Stability. From a 20 mM stock solution in DMSO, FGA146 was diluted to 200 µM in TRIS-buffer (50 mM, pH 7.5), stored at ca. 30 °C and repeatedly analyzed by LCMS (stability in buffer). From a 20 mM stock solution in DMSO, FGA146 was diluted to 200 µM in ACN and repeatedly analyzed by LCMS. Sample was stored at ca. 30 °C or −20 °C in between (stability in DMSO and ACN). Analysis was performed using Mestrenova. LCMS setup: Agilent 1100 series HPLC system coupled to an Agilent 1100 series LC/MSD Trap with electron spray ionization (ESI), Agilent Poroshell 120 EC-C18, 150x2.10 mm, 4 µm column. A linear gradient was used for elution with a ternary pump using [water/ACN/water + 0.1% formic acid] that changes ratios from 80/10/10 to 0/90/10 over the course of 10 min (0.7 mL/min). Areas of peaks in the chromatogram (detection-λ = 326 nm) were used to calculate the change in concentrations. Retention time and mass spectrum recorded in positive ionization mode were used to assign species.

In preparation of experiments with longer incubation periods (metabolic stability, permeability etc.), first general stability in aqueous systems is to be assessed. For this, a TRIS/HCl buffer system at physiological pH was chosen.
In all measurements, only two relevant "species" of FGA146 are identifiable by mass spectrometry: m/z$[M+H_2O+H]^+$ = 504 with t_R = 4.8 min and m/z$[M+H]^+$ = 486 with t_R = 5.1 min.
For quantification by UV AUC, 326 nm is used as detection wavelength. Wavelengths ≤255 nm are unsuitable since extinction coefficients of free and hydrated warhead seem to vastly differ.

Table 10. Stability data of FGA146 in aqueous buffer. For exemplary depiction of chromatograms that were used to generate this table, compare Figure SX below.

t [h]	AUC_{Total} [mAU*min]	AUC_{peak1} [mAU*min]	AUC_{peak1}/ AUC_{Total}	AUC_{peak2} [mAU*min]	AUC_{peak2}/ AUC_{Total}
		t_R = 4.8 min m/z$[M+H_2O+H]^+$ = 504		t_R = 5.1 min m/z$[M+H]^+$ = 486	
0	2.786	0.267	10%	2.519	90%
0.25	2.727	0.541	20%	2.213	80%
0.5	2.744	0.928	34%	1.816	66%
1	2.715	1.271	47%	1.444	53%
1	2.206	1.173	53%	1.033	47%
3	2.171	1.684	78%	0.487	22%
4	1.942	1.772	83%	0.370	17%
5	2.128	1.811	85%	0.317	15%
7	2.164	1.839	85%	0.294	14%
30	2.548	2.116	83%	0.351	14%
31	2.531	2.107	83%	0.351	14%

t = 0 h

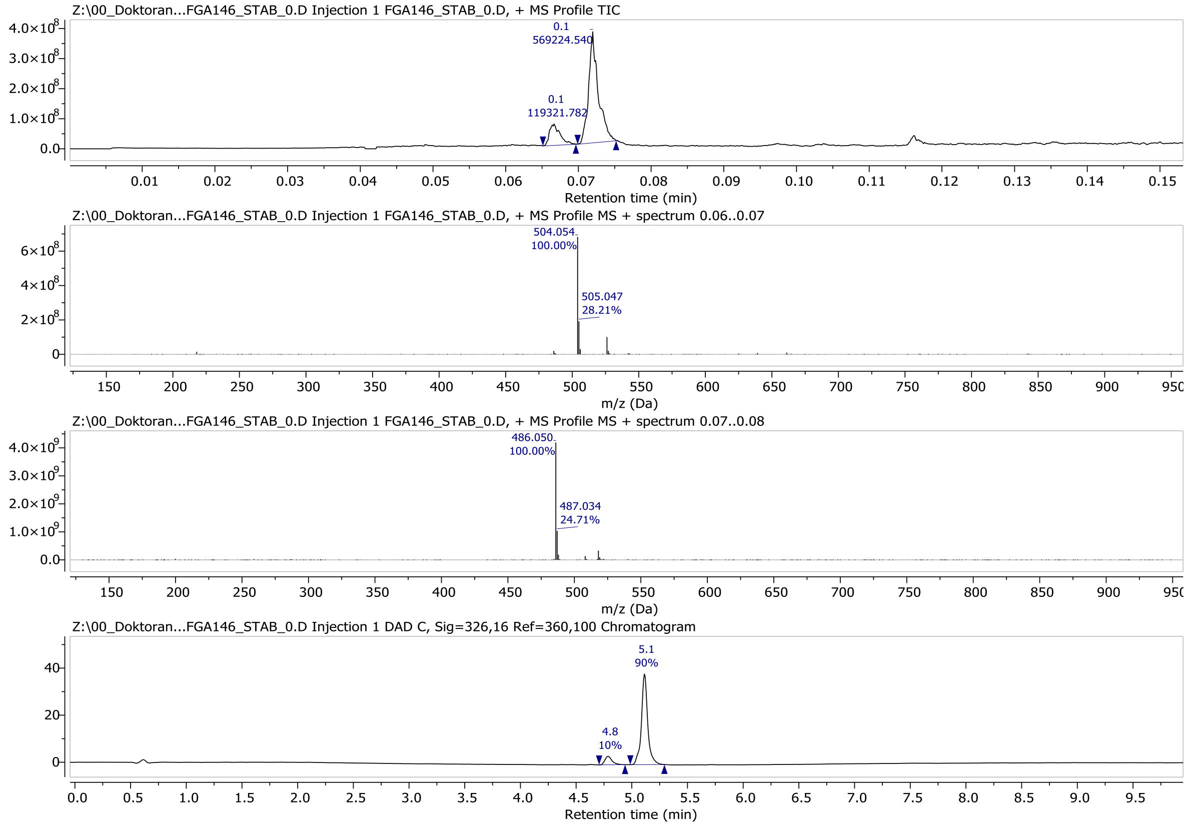

t = 30 h

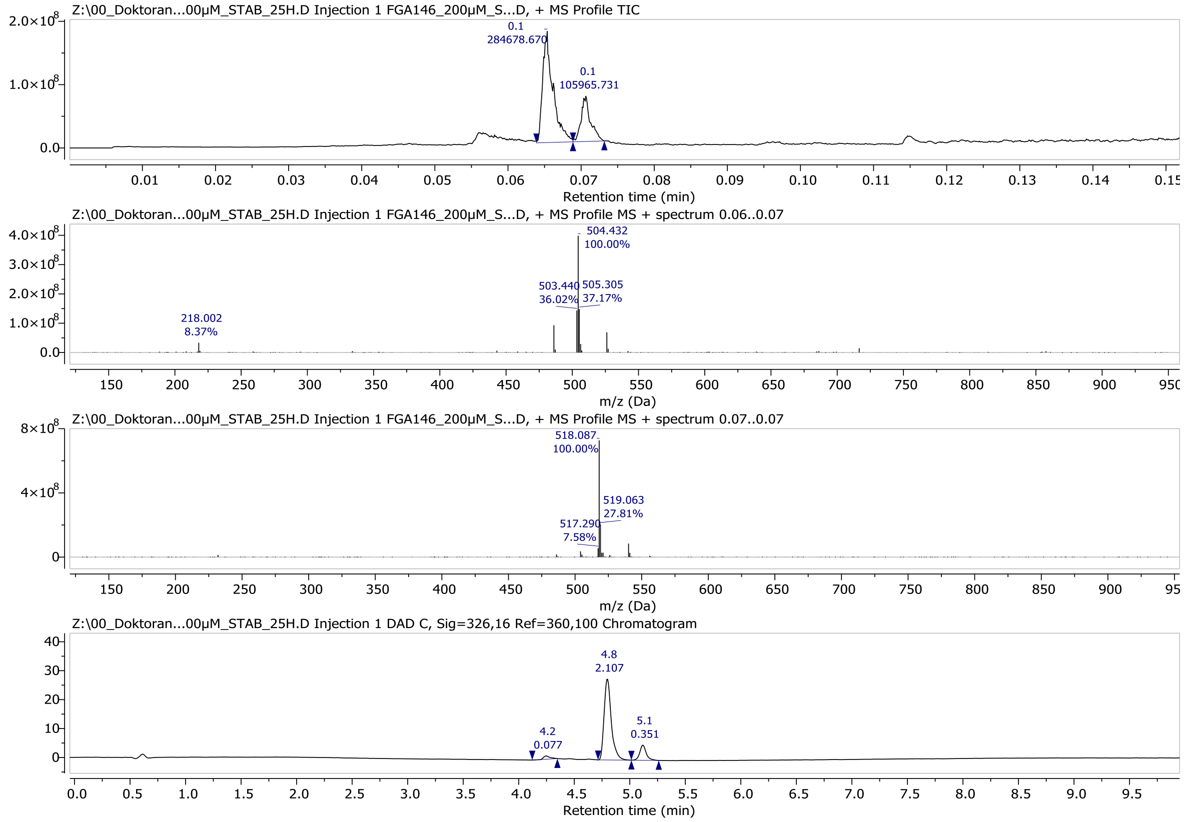

Figure 22. First and last LCMS measurement for the stability of FGA146 in an aqueous buffer. TIC, MS of the two most abundant peaks in their order of retention, and UV-Chromatogram are depicted from top to bottom each. The offset of retention times between the two chromatograms is due to a known instrumental error.

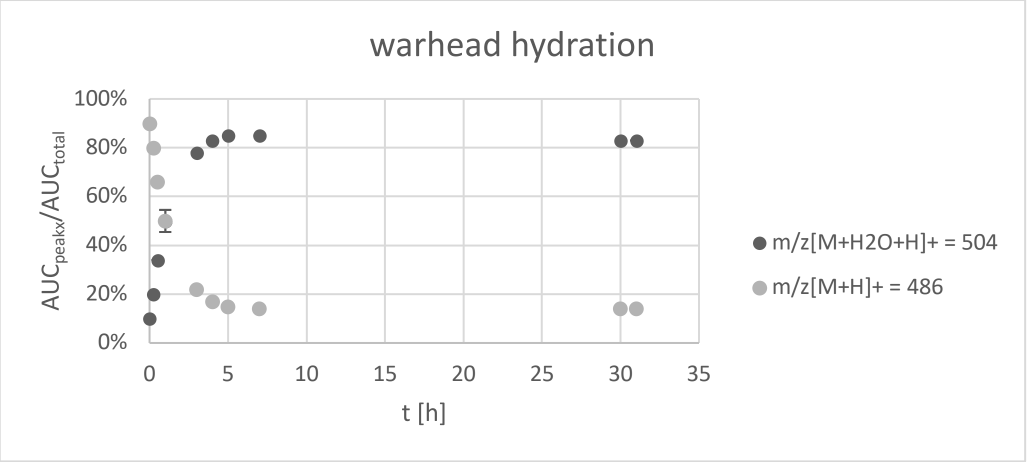

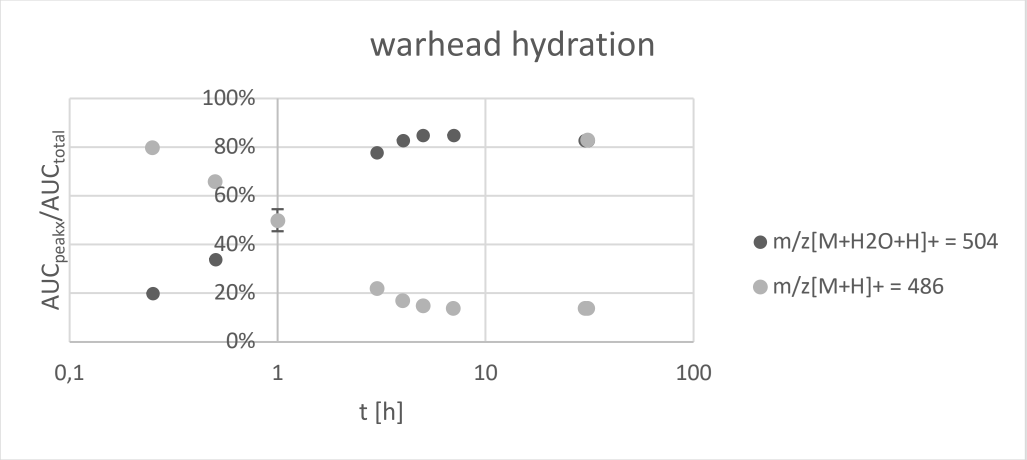

Figure 23. Kinetics of warhead hydration as fraction of the two species (intact inhibitor and putative inhibitor + water product) in linear and logarithmic depiction of time. Only t = 1 h has error bar because for this n = 2, for all others n = 1. Data is combined from two separate time-dependency experiments.

From experience with electrophilic moieties, the LCMS results depicted above indicate the addition of water to the nitro alkene. The product structure was not experimentally elucidated but a reasonable product would be the corresponding 2-hydroxynitroalkane as expected from a Michael-addition-type reaction.

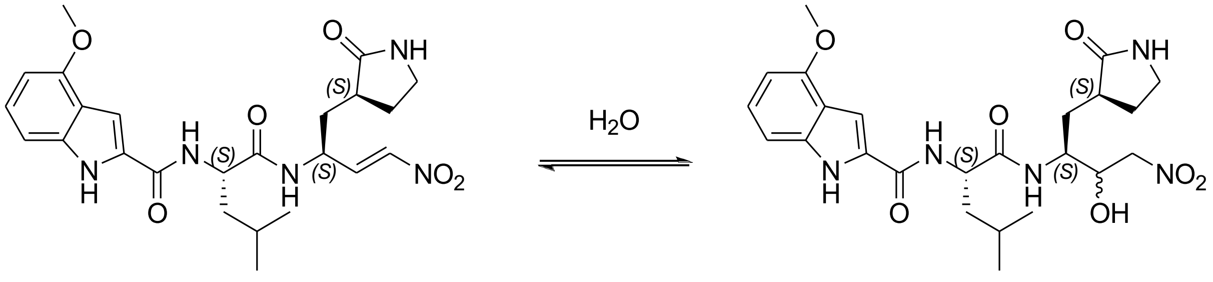

FGA146 **putative product**

Figure 24. Schematic portrayal of the reversible addition of water to the electrophilic warhead of FGA146.

Time to LCMS (<10 min) is enough for 10% conversion to hydrated warhead. 50% conversion can be detected after 1 h, until after 4 h an equilibrium is reached (85/15 hydrated to free warhead). This seems to be the only detected major reaction, but it is expected to impede all studies performed with this substance. Especially so since these results are produced from a minimal system (only buffer at physiological pH). It is expected that all assays in presence of stronger nucleophiles than water (e.g. thiols as DTT in assays or glutathione in cellular context), that take place over a longer period of time (i.e. hours) are ambiguous. Any observed positive effect must be carefully analyzed for artifacts or potential effects beyond the expected inhibition of Cys-proteases.

Dilution assay. M^{Pro} (10 µM) was preincubated with a literature known irreversible M^{Pro} Inhibitor LM188[56] (70 µM); Nirmatrelvir[57] (10 µM) a literature known reversible M^{Pro} Inhibitor; FGA146 (76.4 µM) or DMSO. The preincubation was done in assay buffer (20 mM Tris, 0.1 mM EDTA, 1 mM DTT, 200 mM NaCl, pH = 7.5), at RT for 30 min. After incubation the mixtures were diluted 100fold with assay buffer and substrate (Dabcyl-KTSAVLQSGFRKME-Edans) was added. The proteolytic activity of the enzyme was measured as described for the enzymatic assays using the TECAN Infinite F200 PRO plate reader, except the substrate concentration was 25 µM and the activity was measured over 1 h.

Inhibition assay[58]. Trypsin: Enzyme: bovine trypsin purchased from Sigma-Aldrich (St. Louis, Missouri, USA), [Enzyme]: 3.5 nM, Buffer: 50 mM Tris, 5 mM EDTA, 100 mM NaCl, pH 8, Substrate: Cbz-FR-AMC purchased from Bachem (Bubendorf, Switzerland), [Substrate]: 40 µM (K_M: 85.5 µM), Ex./Em. [nm]: 380/460

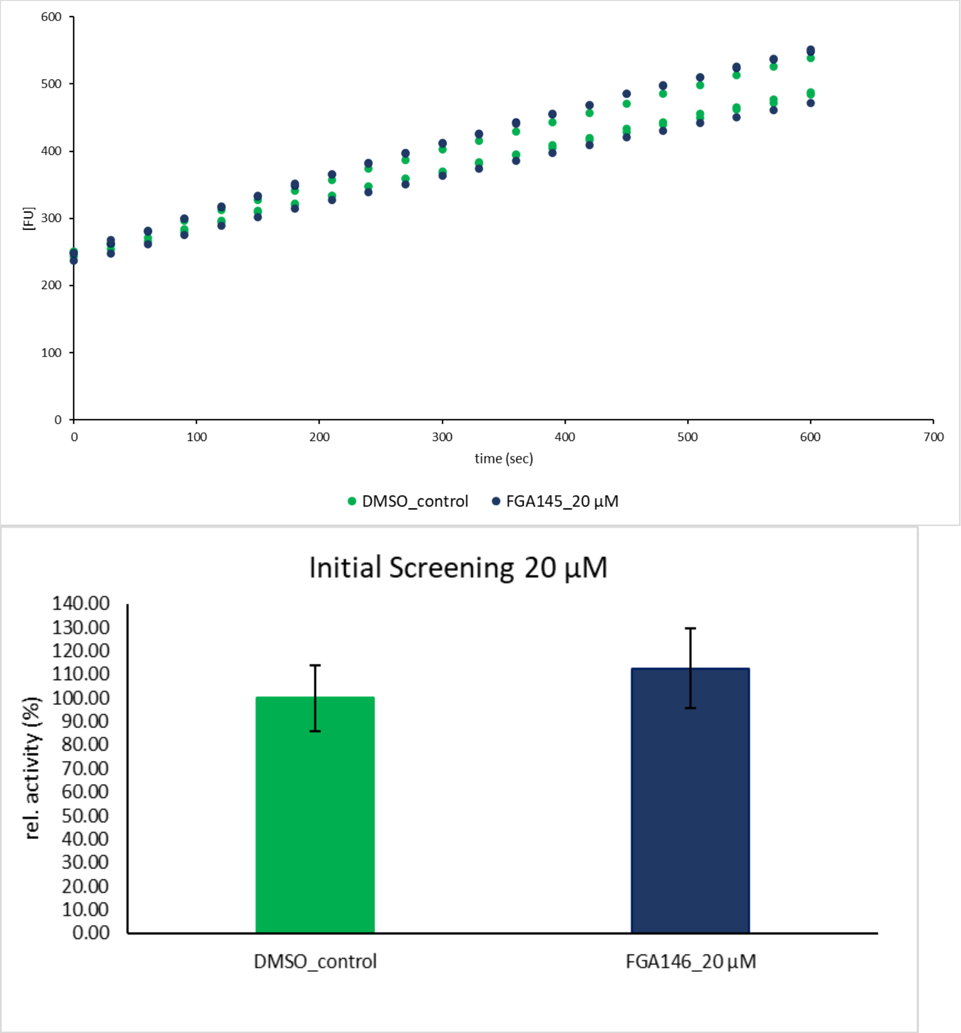

Figure 25. Screening results against bovine trypsin. Above F-t-diagram depicting substrate conversion in presence and absence of FGA146. Below initial slope of F-t-diagram in absence and presence of FGA146 as indicated.

The activity against bovine trypsin was tested at 20 µM inhibitor concentration. The relative activity compared to the DMSO control was 112.5±16.9% and hence no inhibition was detected.

DNMT2 MST-Displacement Assay. Assay was conducted according to literature[59]. The average of quadruple measurements is depicted for two independent measurements each. The positive control was measured without DNMT2 to detect totally unbound FTAD, additionally Sinefungin (SFG), a literature described DNMT2 inhibitor, was used as a second positive control. SFG and FGA146 concentrations in this assay were 100 µM each.
FGA146 was not able to displace FTAD from the active site of DNMT2. Since FGA146 overlaps completely with the negative control (DMSO), FGA146 is defined as a non-binder.

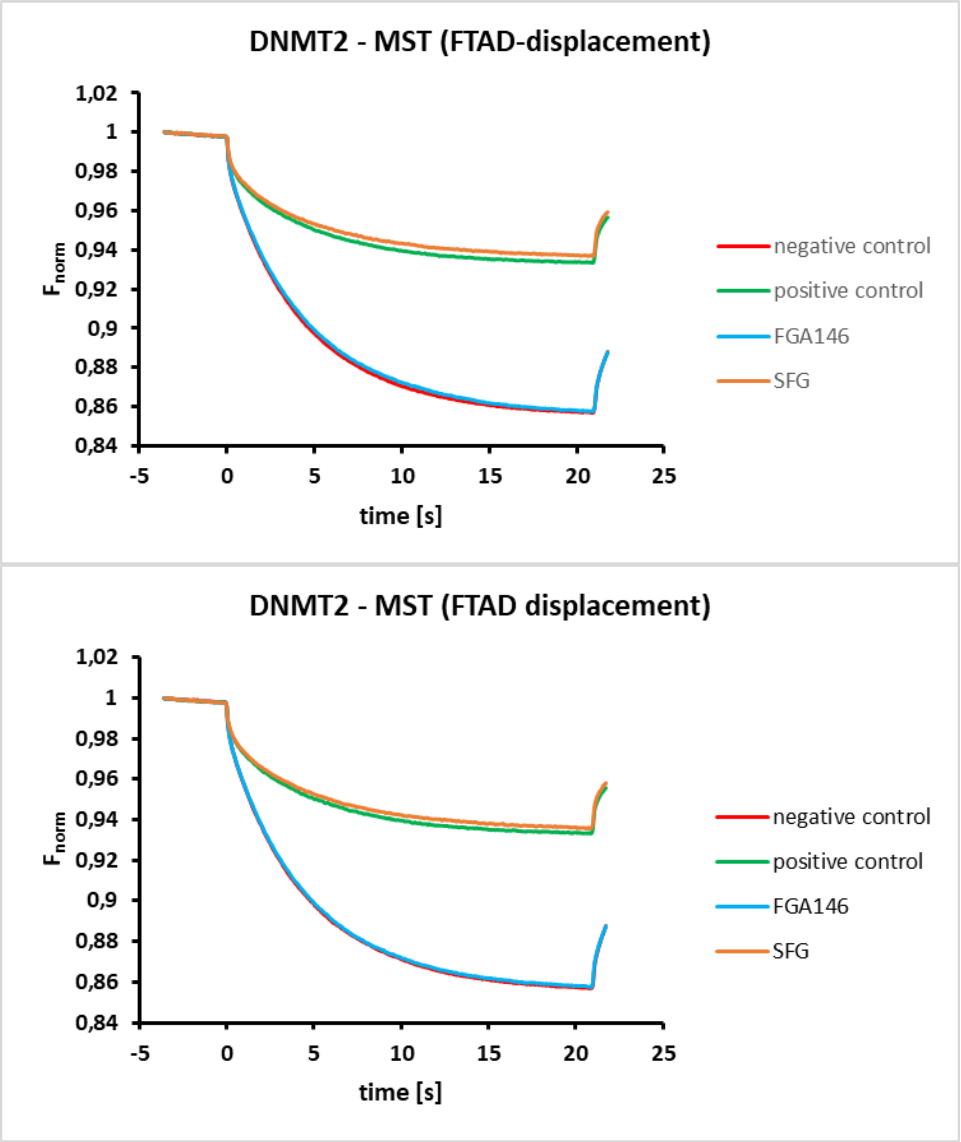

Figure 26. Microscale Thermophoresis displacement experiment against DNMT2.

Other potential off-targets. A search in ChEMBL database for the term “nitroalkene” gave the following papers (excluded 2 synthesis papers using nitroalkenes as reactants)

- https://doi.org/10.1021/acsmedchemlett.6b00276: Nitroalkenes against rhodesain and cruzain
- https://doi.org/10.1021/jm900326c: Nitroalkene fatty acids against PPARγ
- https://doi.org/10.1016/j.ejmech.2012.08.013: Nitroalkene as head group against minor groove binders (anti-infective)

Results one and three are irrelevant due to different organisms as targets. Restult 2 is unlikely to be important, as fatty acid structure deviates from peptidic structure of inhibitors under investigation here.

logP determination by HPLC

In preparation for permeability assays and to verify calculated lipophilicity calculations, the experimental logP was determined. After unsuccessful evaluation by a classical extraction method for determination, an HPLC-based estimation of hydrophobicity by retention time was utilized. Following a publication[60], a set of 9 compounds was chosen for preparing a calibration curve of hydrophobicity indices and logPs.

FGA146 was dissolved in DMSO/ACN (20:80) at 1.6 mg/mL and analyzed via LC (detection-λ = 254 nm, n = 1). Calibration of hydrophobicity was performed with a mixture of thiourea (Merck Millipore, 818591), Theophylline-Monohydrate (Carl Roth), Phenyltetrazole (BLDPharm, BD4691), Benzimidazole (Sigma Aldrich, 194123), Colchicine (Biomol, Cay9000760), Acetophenone (Thermo Fisher Scientific, A12727.AP), Indole (Sigma Aldrich, W259306), Butyrophenone (Merck Schuchardt, 847780025), Valerophenone (Alfa Aesar, A10525) all dissolved in one solution to ca. 1 mg/mL each in 50:50 [50 mM NH_4OAc buffer pH 7.2/ACN][60] (n = 1). Analysis was performed using Mestrenova. LC setup: Alliance waters e2695 LC module coupled to a 2998 PDA detector, Macherey Nagel (720041.40), NUCLEOSIL 120-5 C18 (end-capped), 5 µm, 250x4 mm. A gradient was used for elution with a binary pump using [50 mM NH_4OAc buffer pH 7.2/ACN] (1 mL/min):

0–1.5 min: 100/0
1.5–10.5 min: 100/0 → 0/100
10.5–11.5 min: 0/100
11.5–12.0 min: 0/100 → 100/0
12.0–15.0 min: 100/0

Table 11. Correlations of retention time with reported CHIs and logPs, respectively.

Compound	Chromatographic Hydrophobicity Index (publication)	t_R [min] (measured)	logP (literature)
Theophylline	15.76	6.94	−0.02[61]
Phenyltetrazole	20.18	6.75	−1.0[62]
Benzimidazole	30.71	8.48	0.7[63]
Colchicine	41.37	8.91	1.34[63]
Acetophenone	64.90	9.96	1.7[64]
Indole	69.15	10.31	2.24[65]
Propiophenone	78.41	10.77	2.59[63]
Butyrophenone	88.49	11.39	3.06[63]
Valerophenone	97.67	11.95	3.49[63]

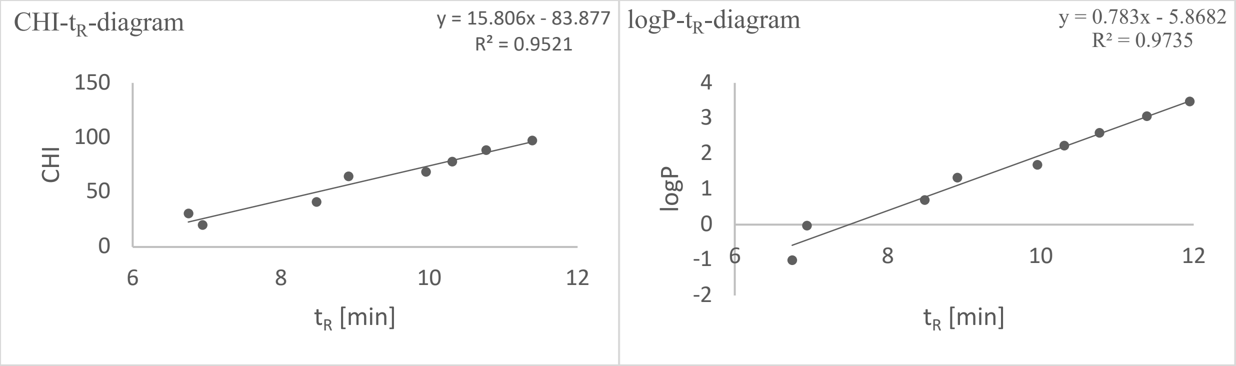

Figure 27. Correlations of retention time with reported CHIs and logPs, respectively.

Correlation between retention time and reported CHIs is sufficient, except for inverted elution of theophylline and 5-phenyl-1*H*-tetrazole. This is however in line with literature data on logP of these two compounds; the correlation between retention and logP is also of sufficient quality. For calculation of unknown logP with this method, the following equation is used:

$$\log P = 0.783 * t_R - 5.8682$$

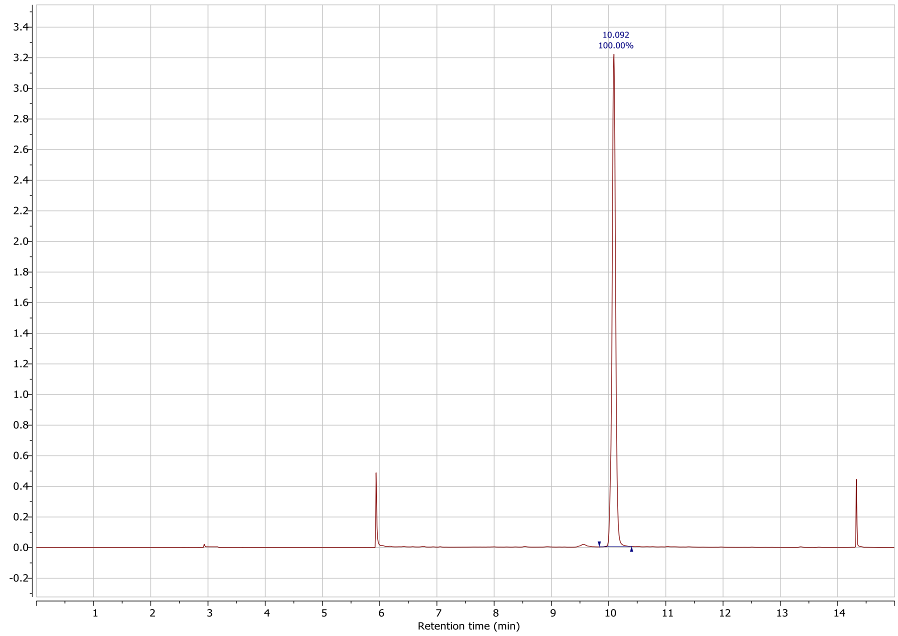

Figure 28. Chromatogram of FGA146 (t_R = 10 min). Signals at 6 and >14 min are due to impurities from column that are detected in all measurements and are therefore not taken into account.

t_R = 10 min
logP = 0.783*10–5.8682 = 2.0
The $\log P_{exp}$ of 2.0 is in good agreement with the predicted $\log P_{calc}$ (2.17).

Permeability. General principle after Lit.[66] Incubation setup: donor (top) plate (Sigma Aldrich, MAIPNTR10), 5 µL artificial membrane (1 % (w/v) L-α-Phosphatidylcholin, Sigma Aldrich P3556, in *n*-dodecane, Sigma Aldrich 8205430100), acceptor (bottom) plate (Greiner, 655074). Measurement setup – UV spectroscopy: UV-transparent measurement plate (Greiner UV-Star®, 655801), Tecan Spark® well plate reader, 200 µL sample volume, λ = 200–650 nm. Analysis was performed with AUC function in GraphPad Prism using the indicated wavelength range.

FGA146 was diluted from 20 mM stock solution in DMSO to 100–400 µM in a buffered (DPBS Sigma D5652; pH 7.4 or TRIS 50 mM pH 7.4) aqueous solution with varying final contents of ACN or DMSO as indicated. ("donor solution"). Similarly prepared solutions (buffer + solvent) were used as "acceptor solutions". 150 µL of donor solution was applied onto the artificial membrane which was applied first to the donor plate. This was sealed (Greiner, 676070, Viewseal sealer). 400 µL of acceptor solution was applied to the acceptor plate. Incubation setup was assembled and left for 2–7 h. After this time, acceptor solution was analyzed with UV spectroscopy. Reference solutions were prepared by simply mixing the indicated volumes of donor and acceptor solutions at the start of the incubation period and analyzed later with the acceptor solutions. Acceptor solution without contact to compound solution was used as buffer reference. Calculations of effective permeability P_e could not be performed due to low permeation. The experiment was performed on three days in at least duplicates each.

50% DMSO/PBS, 400 µM donor concentration (incubation time: 5 h)

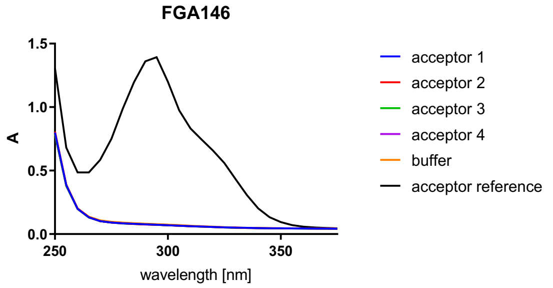

5% ACN/PBS, 100 µM donor concentration (incubation time: 2 h)

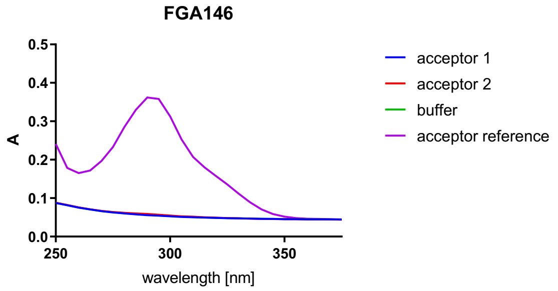

20% DMSO/TRIS, 300 µM donor concentration (incubation time: 7 h)

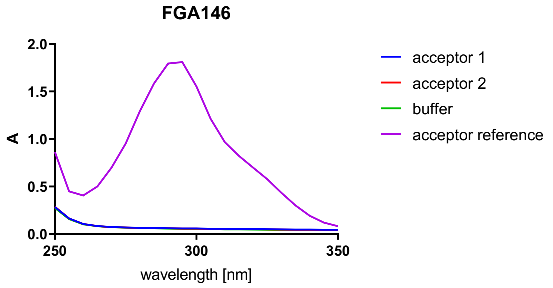

Figure 29. UV-spectra of acceptor and reference solutions of FGA146 after PAMPA. The respective conditions for PAMPA are indicated.

After the incubation period, the reference solution was analyzed by LCMS as described under “stability".

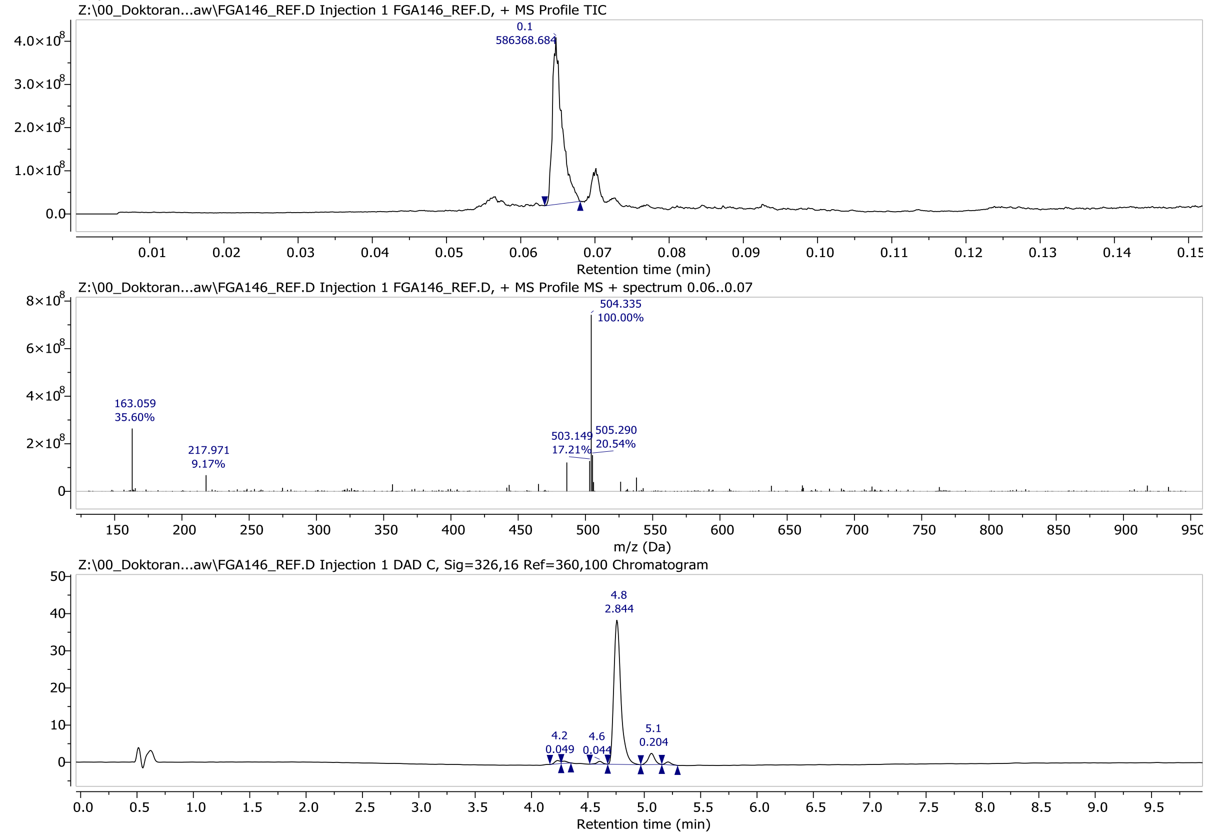

Figure 30. TIC, MS of the most abundant TIC-signal, and UV-Chromatogram of FGA146 reference solution post incubation period. The offset of retention times between the two chromatograms is due to a known instrumental error.

No permeation can be observed in any of the UV-quantified experiments. From LCMS-experiment it can be inferred that the most dominant species after incubation is the putative 2-hydroxy nitroalkane, which is in line with the described stability measurements.

Supplementary References

1. Amendola, G. et al. Lead Discovery of SARS-CoV-2 Main Protease Inhibitors through Covalent Docking-Based Virtual Screening. *J Chem Inf Model* **61**, 2062-2073 (2021).
2. Désilets, A., Longpre, J.M., Beaulieu, M.E. & Leduc, R. Inhibition of human matriptase by eglin c variants. *FEBS Lett* **580**, 2227-32 (2006).
3. Phoo, W.W. et al. Structure of the NS2B-NS3 protease from Zika virus after self-cleavage. *Nature Communications* **7**, 13410 (2016).
4. Klein, P. et al. New Cysteine Protease Inhibitors: Electrophilic (Het)arenes and Unexpected Prodrug Identification for the Trypanosoma Protease Rhodesain. *Molecules* **25**(2020).
5. Leatherbarrow, R.J. GraFit 6. Vol. Erithacus Software Limited: East Gristead, West Sussex (2007).
6. Ludewig, S., Kossner, M., Schiller, M., Baumann, K. & Schirmeister, T. Enzyme kinetics and hit validation in fluorimetric protease assays. *Curr Top Med Chem* **10**, 368-82 (2010).
7. Hoffmann, M. et al. SARS-CoV-2 Cell Entry Depends on ACE2 and TMPRSS2 and Is Blocked by a Clinically Proven Protease Inhibitor. *Cell* **181**, 271-280.e8 (2020).
8. Müller, C. et al. Broad-spectrum antiviral activity of the eIF4A inhibitor silvestrol against corona- and picornaviruses. *Antiviral Res* **150**, 123-129 (2018).
9. Kabsch, W. XDS. *Acta Crystallographica Section D* **66**, 125-132 (2010).
10. Vagin, A. & Teplyakov, A. Molecular replacement with MOLREP. *Acta Crystallogr D Biol Crystallogr* **66**, 22-5 (2010).
11. Murshudov, G.N., Vagin, A.A. & Dodson, E.J. Refinement of macromolecular structures by the maximum-likelihood method. *Acta Crystallogr D Biol Crystallogr* **53**, 240-55 (1997).
12. Emsley, P., Lohkamp, B., Scott, W.G. & Cowtan, K. Features and development of Coot. *Acta Crystallogr D Biol Crystallogr* **66**, 486-501 (2010).
13. Winn, M.D. et al. Overview of the CCP4 suite and current developments. *Acta Crystallogr D Biol Crystallogr* **67**, 235-42 (2011).
14. Chen, V.B. et al. MolProbity: all-atom structure validation for macromolecular crystallography. *Acta Crystallogr D Biol Crystallogr* **66**, 12-21 (2010).
15. Braun, N.J. et al. Structure-Based Macrocyclization of Substrate Analogue NS2B-NS3 Protease Inhibitors of Zika, West Nile and Dengue viruses. *ChemMedChem* **15**, 1439-1452 (2020).
16. Millies, B. et al. Proline-Based Allosteric Inhibitors of Zika and Dengue Virus NS2B/NS3 Proteases. *J Med Chem* **62**, 11359-11382 (2019).
17. Phoo, W.W. et al. Structure of the NS2B-NS3 protease from Zika virus after self-cleavage. *Nat Commun* **7**, 13410 (2016).
18. Zhai, X. & Meek, T.D. Catalytic Mechanism of Cruzain from Trypanosoma cruzi As Determined from Solvent Kinetic Isotope Effects of Steady-State and Pre-Steady-State Kinetics. *Biochemistry* **57**, 3176-3190 (2018).
19. Vicik, R. et al. Aziridide-based inhibitors of cathepsin L: synthesis, inhibition activity, and docking studies. *ChemMedChem* **1**, 1126-41 (2006).
20. Ettari, R. et al. Development of peptidomimetics with a vinyl sulfone warhead as irreversible falcipain-2 inhibitors. *J Med Chem* **51**, 988-96 (2008).
21. Zvornicanin, S.N. et al. Crystal Structures of Inhibitor-Bound Main Protease from Delta- and Gamma-Coronaviruses. *Viruses* **15**(2023).
22. Wang, Y.C. et al. Structural basis of SARS-CoV-2 main protease inhibition by a broad-spectrum anti-coronaviral drug. *Am J Cancer Res* **10**, 2535-2545 (2020).
23. Jin, Z. et al. Structure of Mpro from SARS-CoV-2 and Discovery of its Inhibitors. *Nature* **582**, 289-293 (2020).
24. Świderek, K. & Moliner, V. Revealing the Molecular Mechanisms of Proteolysis of SARS-CoV-2 Mpro from QM/MM Computational Methods. *Chemical Science* **11**, 10626-10630 (2020).
25. Arafet, K. et al. Mechanism of Inhibition of SARS-CoV-2 Mpro by N3 Peptidyl Michael Acceptor Explained by QM/MM Simulations and Design of New Derivatives with Tunable Chemical Reactivity. *Chemical Science* **12**, 1433-1444 (2021).

26. Martí, S., Arafet, K., Lodola, A., Mulholland, A.J., Świderek, K. & Moliner, V. Impact of Warhead Modulations on the Covalent Inhibition of SARS-CoV-2 Mpro Explored by QM/MM Simulations. *ACS Catalysis* **12**, 698-708 (2022).
27. Wang, J., Wang, W., Kollman, P.A. & Case, D.A. Automatic Atom Type and Bond Type Perception in Molecular Mechanical Calculations. *Journal of Molecular Graphics and Modelling* **25**, 247-260 (2006).
28. Olsson, M.H.M., Sondergaard, C.R., Rostkowski, M. & Jensen, J.H. PROPKA3: Consistent Treatment of Internal and Surface Residues in Empirical pKa Predictions. *Journal of Chemical Theory and Computation* **7**, 525-537 (2011).
29. Jorgensen, W.L., Chandrasekhar, J., Madura, J.D., Impey, R.W. & Klein, M.L. Comparison of Simple Potential Functions for Simulating Liquid Water. *Journal of Chemical Physics* **79**, 926-935 (1983).
30. Duan, Y. et al. A Point-charge Force Field for Molecular Mechanics Simulations of Proteins Based on Condensed-phase Quantum Mechanical Calculations. *Journal of Computational Chemistry* **24**, 1999-2012 (2003).
31. Phillips, J.C. et al. Scalable Molecular Dynamics with NAMD. *Journal of Computational Chemistry* **26**, 1781-1802 (2005).
32. Grest, G.S. & Kremer, K. Molecular Dynamics Simulation for Polymers in the Presence of a Heat Bath. *Physical Review A* **33**, 3628-3631 (1986).
33. Roe, D.R. & Cheatham, T.E. PTRAJ and CPPTRAJ: Software for Processing and Analysis of Molecular Dynamics Trajectory Data. *Journal of Chemical Theory and Computation* **9**, 3084-3095 (2013).
34. Martí, S. QMCube (QM3): An All-purpose Suite for Multiscale QM/MM Calculations. *Journal of Computational Chemistry* **42**, 447-457 (2021).
35. M. J. Frisch et al. Gaussian 09 (Revision A.1). (Wallingford, CT, 2009).
36. Zhao, Y. & Truhlar, D.G. The M06 Suite of Density Functionals for Main Group Thermochemistry, Thermochemical Kinetics, Noncovalent Interactions, Excited States, and Transition Elements: Two New Functionals and Systematic Testing of Four M06-class Functionals and 12 Other Functionals. *Theoretical Chemistry Accounts* **120**, 215-241 (2008).
37. Hehre, W.J., Radom, L., Schleyer, P.V.R. & Pople, J.A. *Ab Initio Molecular Orbital Theory*, (John Wiley, New York, 1986).
38. Arafet, K., Ferrer, S. & Moliner, V. Computational Study of the Catalytic Mechanism of the Cruzain Cysteine Protease. *ACS Catalysis* **7**, 1207-1215 (2017).
39. Arafet, K., Świderek, K. & Moliner, V. Computational Study of the Michaelis Complex Formation and the Effect on the Reaction Mechanism of Cruzain Cysteine Protease. *ACS Omega* **3**, 18613-18622 (2018).
40. Arafet, K., González, F.V. & Moliner, V. Quantum Mechanics/Molecular Mechanics Studies of the Mechanism of Cysteine Proteases Inhibition by Dipeptidyl Nitroalkenes. *Chemistry-A European Journal* **26**, 2002-2012 (2020).
41. Arafet, K., González, F.V. & Moliner, V. Elucidating the Dual Mode of Action of Dipeptidyl Enoates in the Inhibition of Rhodesain Cysteine Proteases. *Chemistry – A European Journal* **27**, 10142-10150 (2021).
42. Chan, H.T.H. et al. Discovery of SARS-CoV-2 Mpro Peptide Inhibitors from Modelling Substrate and Ligand Binding. *Chemical Science* **12**, 13686-13703 (2021).
43. Henkelman, G. & Jónsson, H. Improved tangent estimate in the nudged elastic band method for finding minimum energy paths and saddle points. *The Journal of Chemical Physics* **113**, 9978-9985 (2000).
44. Torrie, G.M. & Valleau, J.P. Non-physical Sampling Distributions in Monte_Carlo Free-Energy Estimation-Umbrella Sampling. *Journal of Computational Physics* **23**, 187-199 (1977).
45. Kumar, S., Bouzida, D., Swendsen, R.H., Kollman, P.A. & Rosenberg, J.M. The Weighted Histogram Analysis Method for Free-Energy Calculations on Biomolecules.1. The Method. *Journal of Computational Chemistry* **13**, 1011-1021 (1992).
46. Dewar, M.J.S., Zoebisch, E.G., Healy, E.F. & Stewart, J.J.P. The Development and Use of Quantum Mechanical Molecular-models. 76. AM1: A New General Purpose Quantum Mechanical Molecular Model. *J. Am. Chem. Soc.* **107**, 3902-3909 (1985).

47. Martí, S., Arafet, K., Lodola, A., Mulholland, A.J., Swiderek, K. & Moliner, V. Impact of Warhead Modulations on the Covalent Inhibition of SARS-CoV-2 M(pro) Explored by QM/MM Simulations. *ACS Catal* **12**, 698-708 (2022).
48. Jin, Z. et al. Structure of M(pro) from SARS-CoV-2 and discovery of its inhibitors. *Nature* **582**, 289-293 (2020).
49. Günther, S. et al. X-ray screening identifies active site and allosteric inhibitors of SARS-CoV-2 main protease. *Science* **372**, 642-646 (2021).
50. Kovalevsky, A. et al. Unmasking the Conformational Stability and Inhibitor Binding to SARS-CoV-2 Main Protease Active Site Mutants and Miniprecursor. *Journal of Molecular Biology* **434**, 167876 (2022).
51. Lu, J. et al. Crystallization of Feline Coronavirus Mpro With GC376 Reveals Mechanism of Inhibition. *Frontiers in Chemistry* **10**(2022).
52. Nashed, N.T. et al. Autoprocessing and oxyanion loop reorganization upon GC373 and nirmatrelvir binding of monomeric SARS-CoV-2 main protease catalytic domain. *Communications Biology* **5**, 976 (2022).
53. Sacco, M.D. et al. The P132H mutation in the main protease of Omicron SARS-CoV-2 decreases thermal stability without compromising catalysis or small-molecule drug inhibition. *Cell Research* **32**, 498-500 (2022).
54. Barthels, F. et al. 2-Sulfonylpyrimidines as Privileged Warheads for the Development of S. aureus Sortase A Inhibitors. *Front Mol Biosci* **8**, 804970 (2021).
55. Jeong, E.M. et al. Real-Time Monitoring of Glutathione in Living Cells Reveals that High Glutathione Levels Are Required to Maintain Stem Cell Function. *Stem Cell Reports* **10**, 600-614 (2018).
56. Müller, P. et al. Investigation of the Compatibility between Warheads and Peptidomimetic Sequences of Protease Inhibitors-A Comprehensive Reactivity and Selectivity Study. *Int J Mol Sci* **24**(2023).
57. Owen, D.R. et al. An oral SARS-CoV-2 M(pro) inhibitor clinical candidate for the treatment of COVID-19. *Science* **374**, 1586-1593 (2021).
58. Maus, H. et al. SAR of novel benzothiazoles targeting an allosteric pocket of DENV and ZIKV NS2B/NS3 proteases. *Bioorg Med Chem* **47**, 116392 (2021).
59. Zimmermann, R.A., Schwickert, M., Meidner, J.L., Nidoieva, Z., Helm, M. & Schirmeister, T. An Optimized Microscale Thermophoresis Method for High-Throughput Screening of DNA Methyltransferase 2 Ligands. *ACS Pharmacol Transl Sci* **5**, 1079-1085 (2022).
60. Valkó, K., Bevan, C. & Reynolds, D. Chromatographic Hydrophobicity Index by Fast-Gradient RP-HPLC: A High-Throughput Alternative to log P/log D. *Anal Chem* **69**, 2022-9 (1997).
61. Schierle, S. et al. Boosting Anti-Inflammatory Potency of Zafirlukast by Designed Polypharmacology. *J Med Chem* **61**, 5758-5764 (2018).
62. Borhade, S.R., Svensson, R., Brandt, P., Artursson, P., Arvidsson, P.I. & Sandstrom, A. Preclinical characterization of acyl sulfonimidamides: potential carboxylic acid bioisosteres with tunable properties. *ChemMedChem* **10**, 455-60 (2015).
63. Reis, J. et al. Design and synthesis of chromone-based monoamine oxidase B inhibitors with improved drug-like properties. *Eur J Med Chem* **239**, 114507 (2022).
64. OECD Guideline for the Testing of Chemicals. 1-11 (2004).
65. Toulmin, A., Wood, J.M. & Kenny, P.W. Toward prediction of alkane/water partition coefficients. *J Med Chem* **51**, 3720-30 (2008).
66. Kansy, M., Senner, F. & Gubernator, K. Physicochemical high throughput screening: parallel artificial membrane permeation assay in the description of passive absorption processes. *J Med Chem* **41**, 1007-10 (1998).
