# Supplementary material for: Peptidyl nitroalkene inhibitors of main protease rationalized by computational and crystallographic investigations as antivirals against SARS-CoV-2
Source: Commun Chem. 2024 Jan 18;7:15. doi: 10.1038/s42004-024-01104-7 (PMC10796436; doi:10.1038/s42004-024-01104-7)
Supplement: Supplementary file 3 — Description of Additional Supplementary Files [file 42004_2024_1104_MOESM3_ESM.pdf]

Description of Additional Supplementary Files

File name: Supplementary Data 1

Description: cif file of structure 8BGA

File name: Supplementary Data 2

Description: cif file of structure 8BGD

File name: Supplementary Data 3

Description: NMR Spectra

File name: Supplementary Data 4

Description: : EC50 calculations

File name: Supplementary Data 5

Description: ITC experiments

File name: Supplementary Data 6

Description: Dilution assays
