# Supplementary material for: Peptidyl nitroalkene inhibitors of main protease rationalized by computational and crystallographic investigations as antivirals against SARS-CoV-2
Source: Commun Chem. 2024 Jan 18;7:15. doi: 10.1038/s42004-024-01104-7 (PMC10796436; doi:10.1038/s42004-024-01104-7)
Supplement: Supplementary file 6 — Supplementary Data 3 [file 42004_2024_1104_MOESM6_ESM.docx]

***tert*-Butyl ((2*S*)-1-hydroxy-3-(2-oxopyrrolidin-3-yl)propan-2-yl)carbamate 1**

***
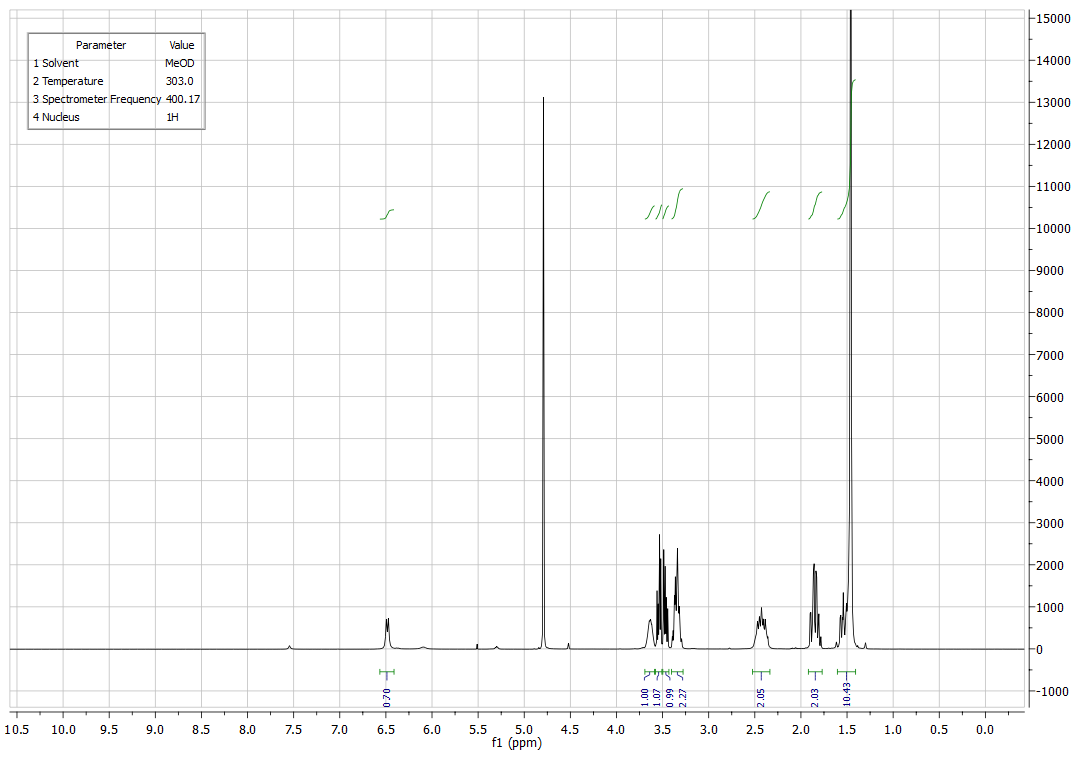
***

***
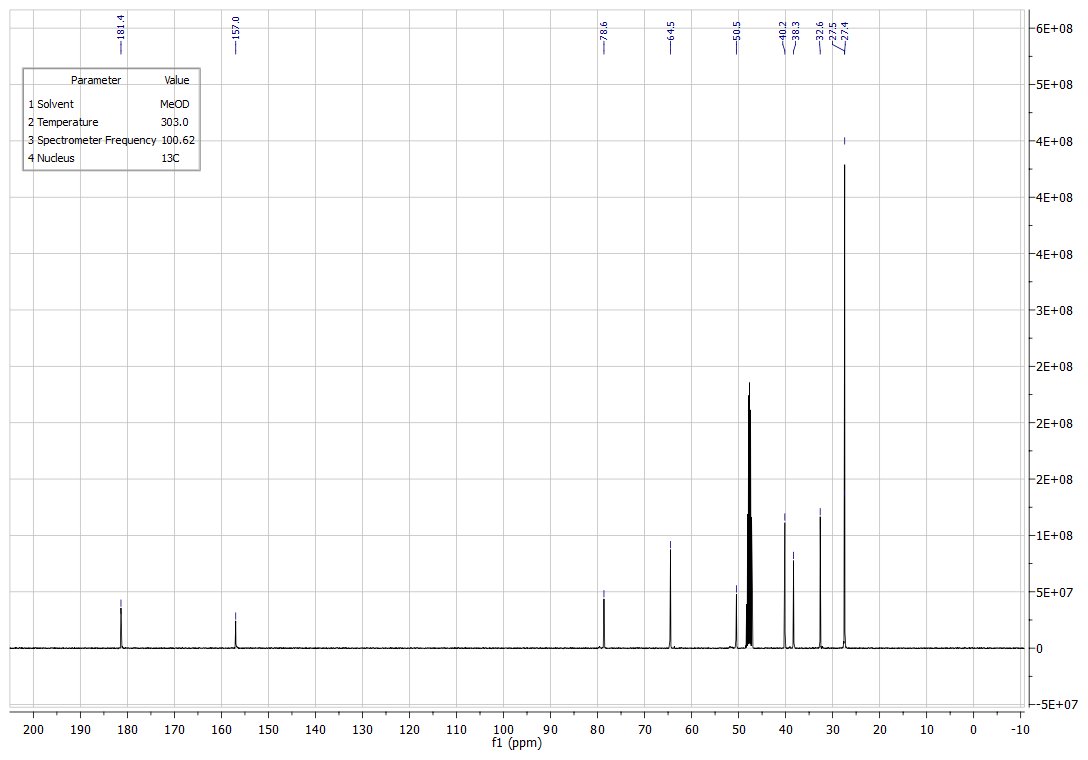
***

***tert*-Butyl ((2*S*)-3-hydroxy-4-nitro-1-(2-oxopyrrolidin-3-yl)butan-2-yl)carbamate 2.**


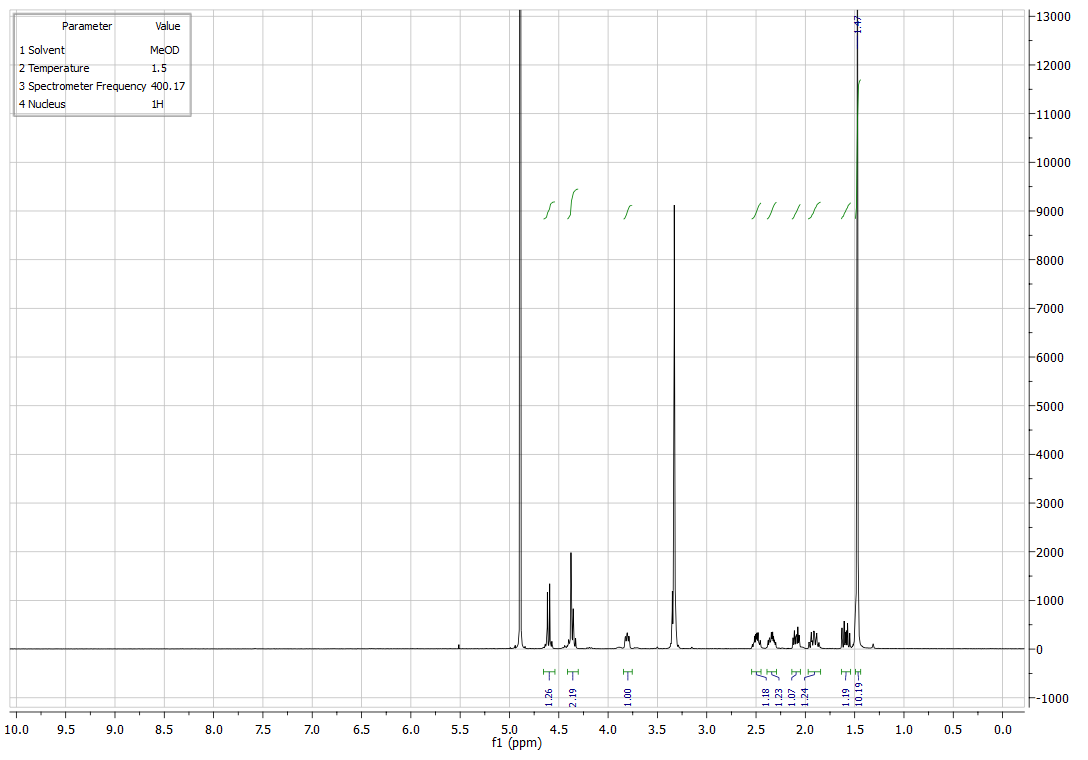


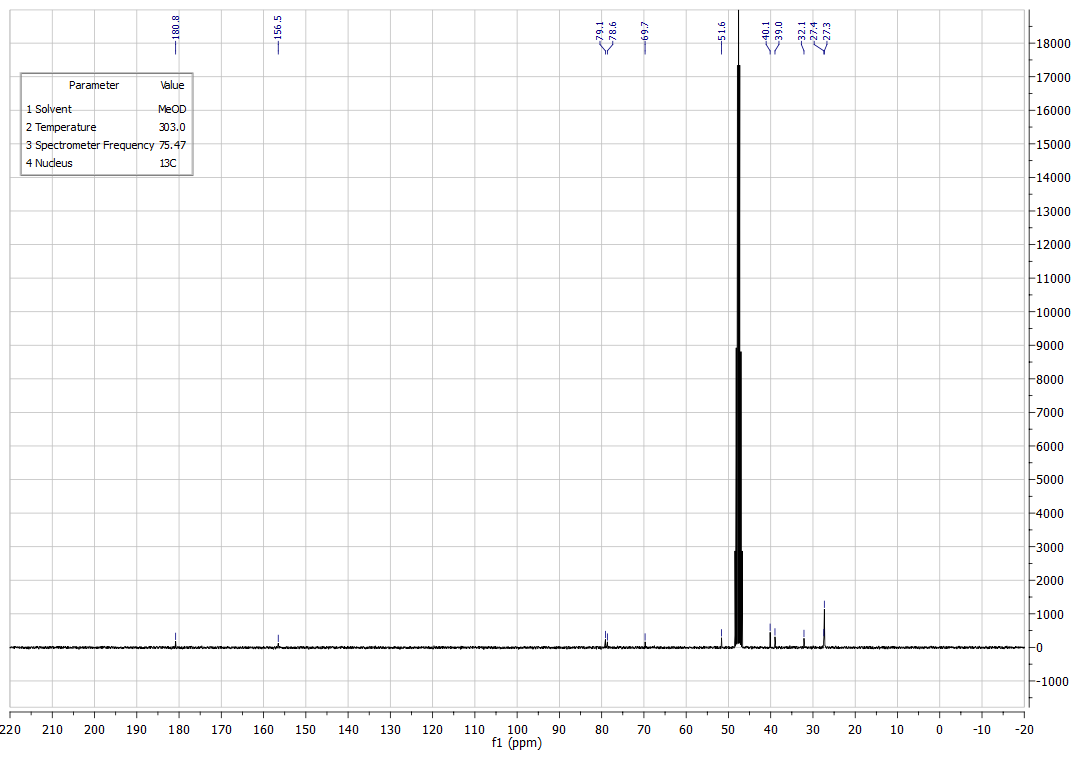


**Benzyl ((2*S*)-1-(((2*S*)-3-hydroxy-4-nitro-1-(2-oxopyrrolidin-3-yl)butan-2-yl)amino)-4-methyl-1-oxopentan-2-yl)carbamate 3*.***

*
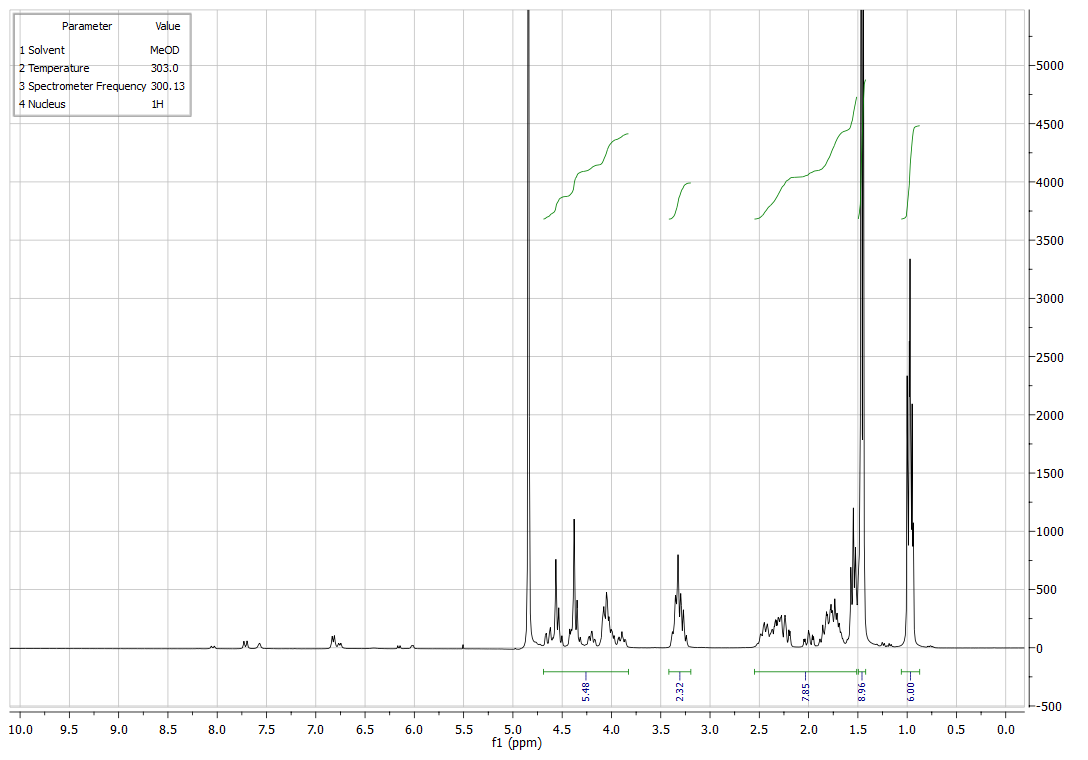
*

*
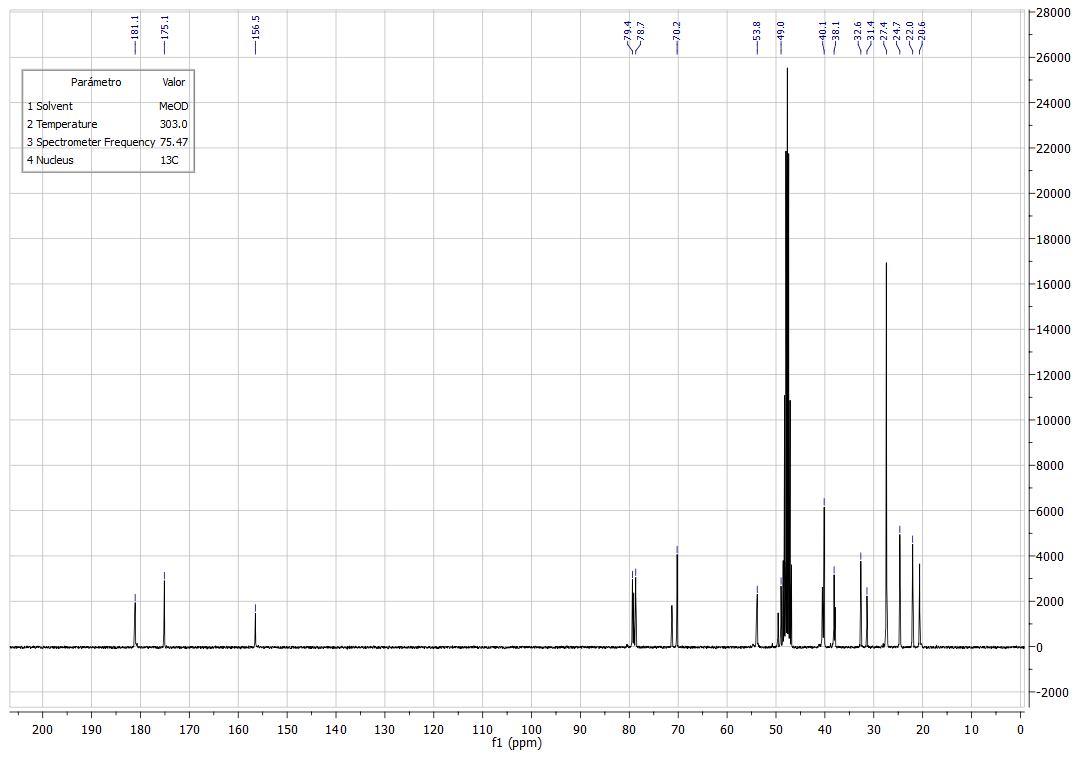
*

*
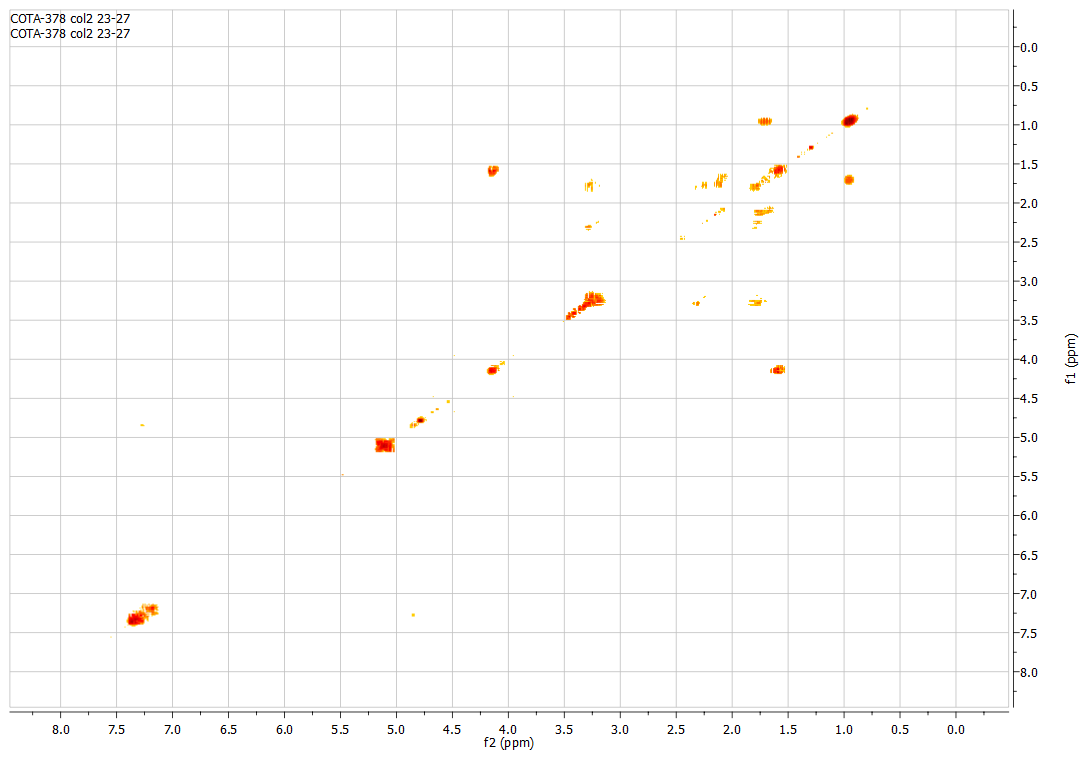

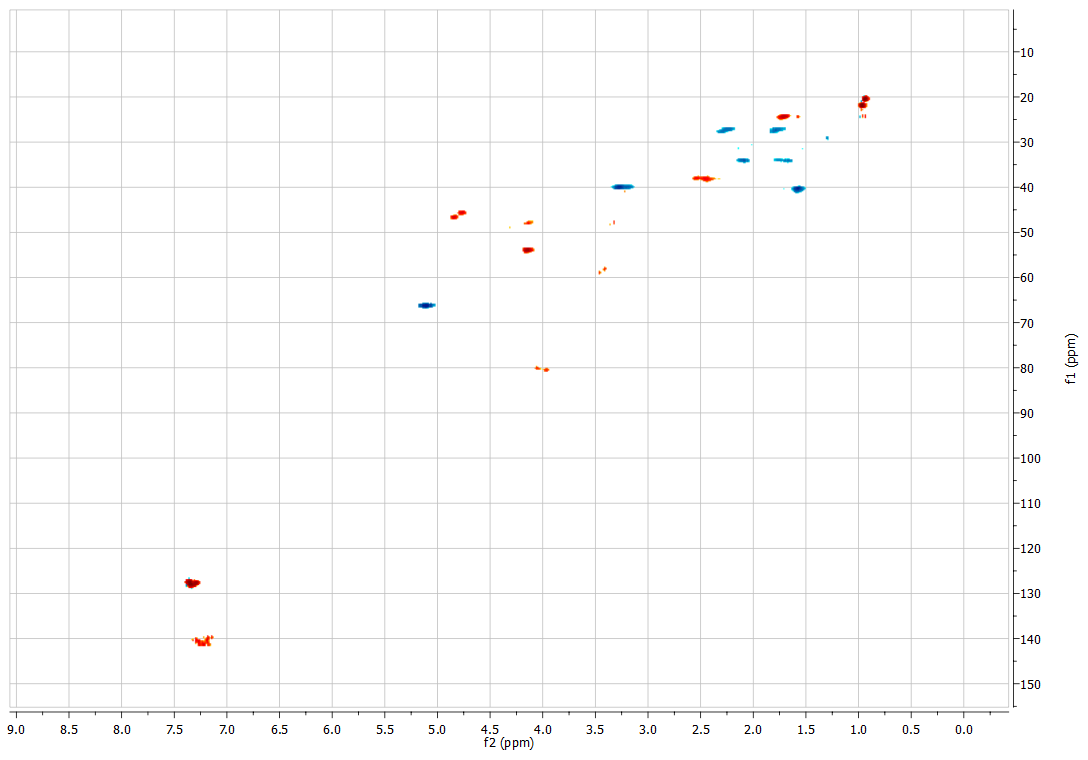
*

***tert*-Butyl ((3*S*)-2-hydroxy-1-nitro-5-phenylpentan-3-yl)carbamate 4.**

*
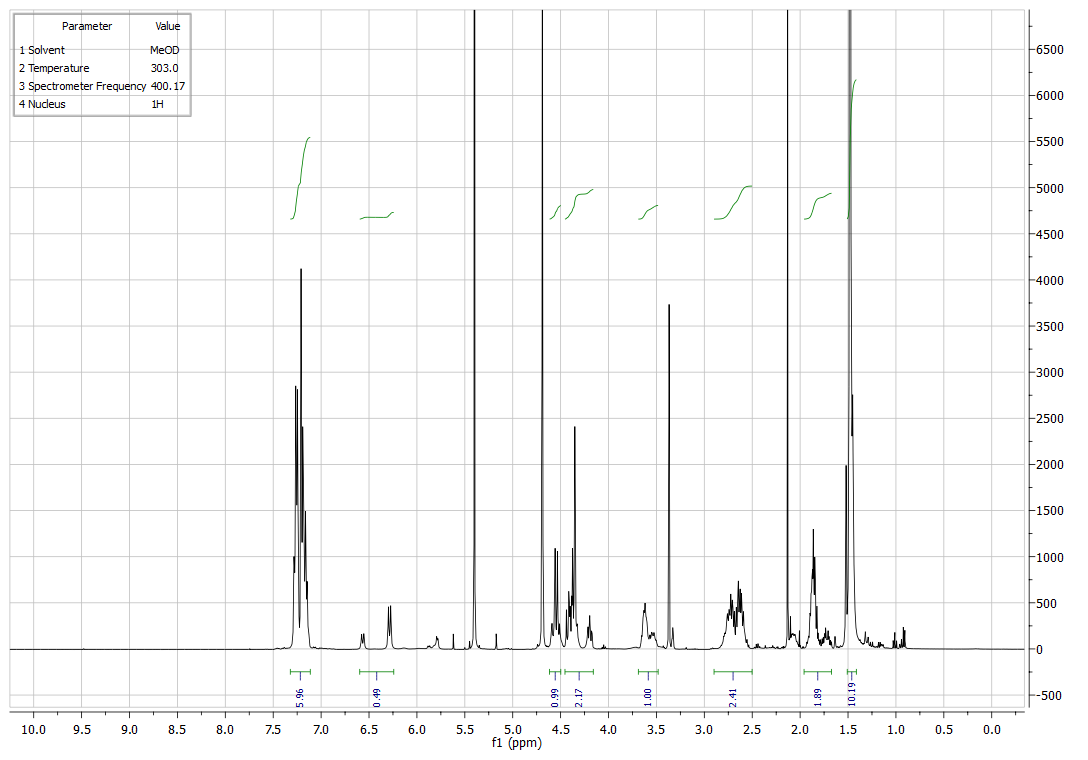
*

*
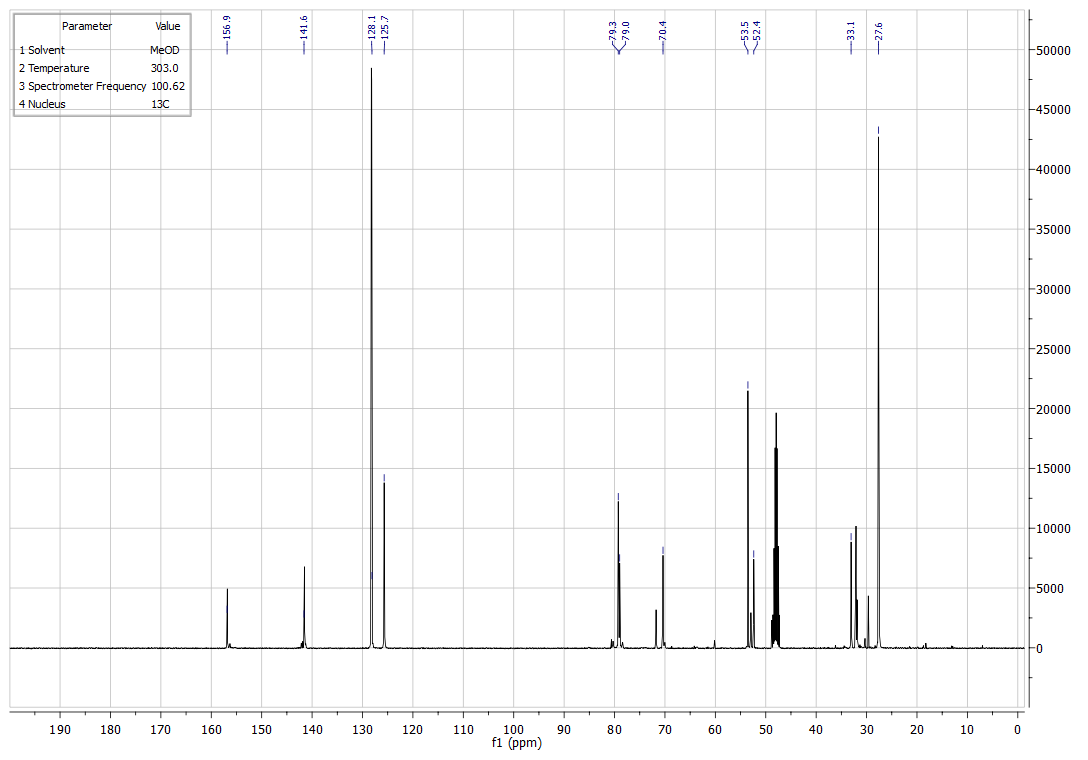
*

**Methyl (*tert*-butoxycarbonyl)-*L*-alanyl-*L*-valinate 6.**

*
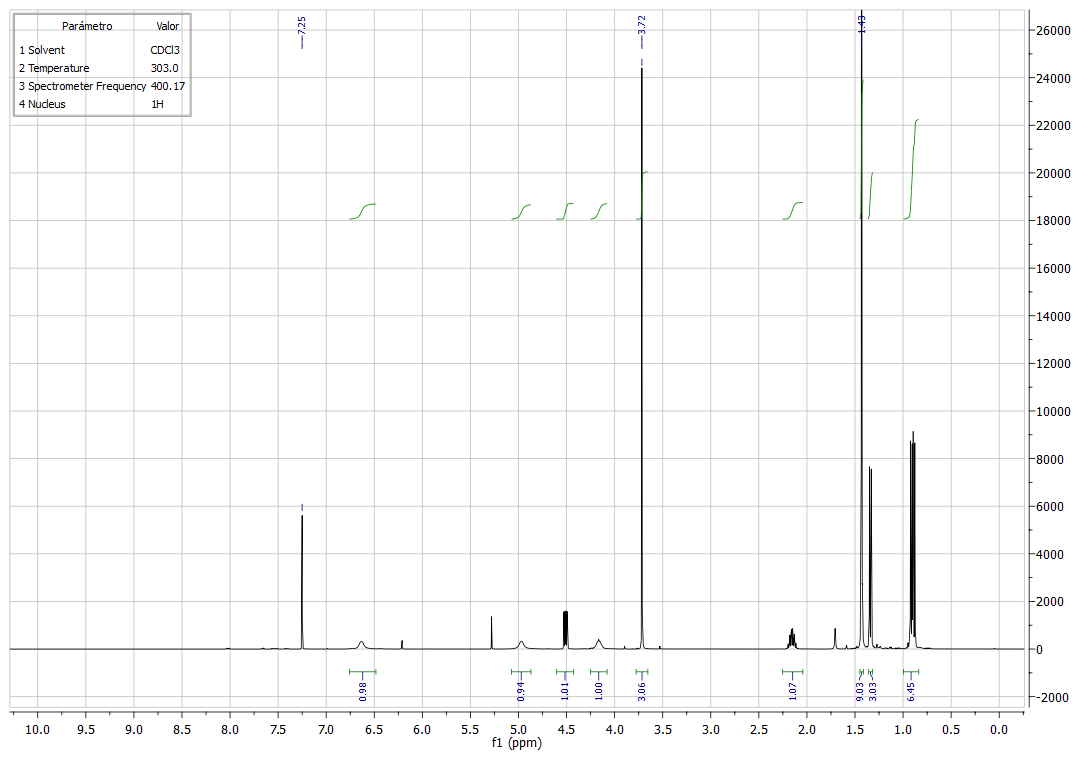
*

*
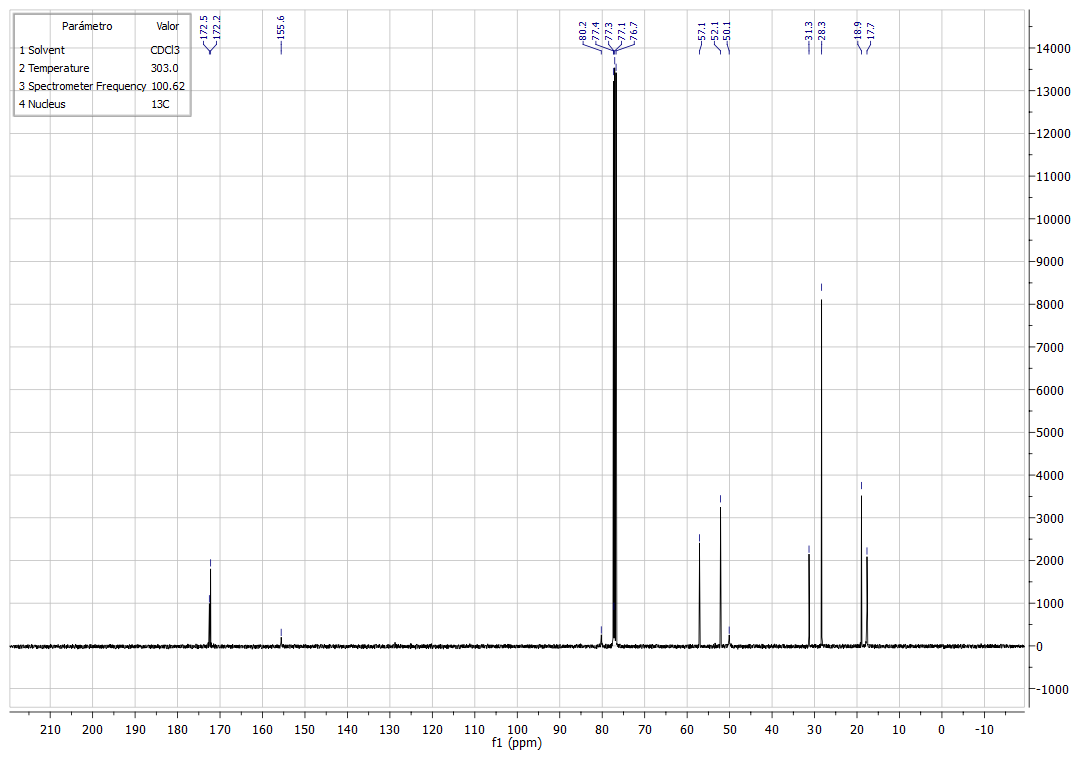
*

**Benzyl (*tert*-butoxycarbonyl)-*L*-alanyl-*L*-valyl-*L*-leucinate 7.**

*
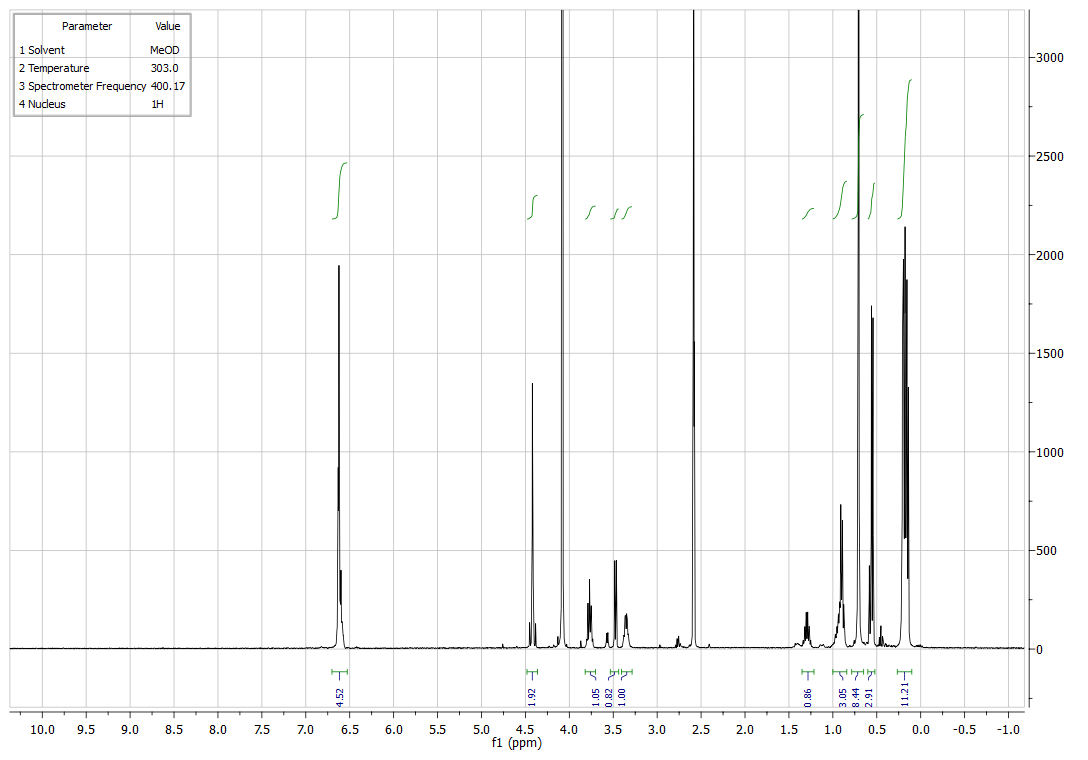
*

*
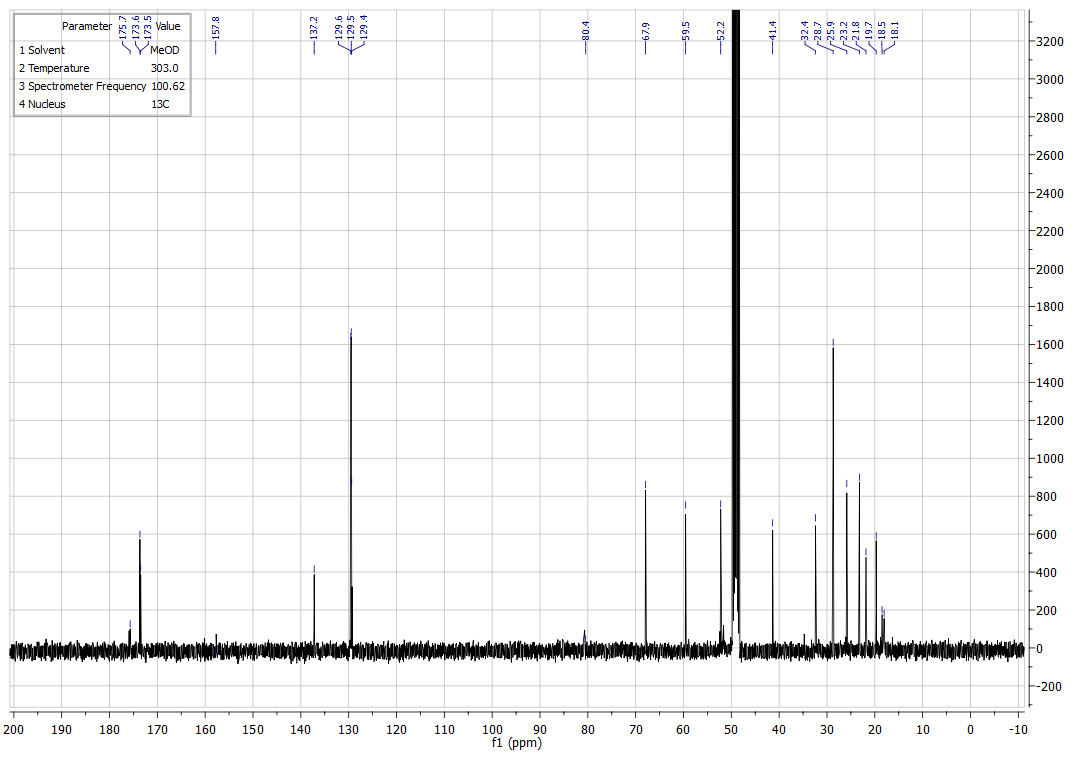
*

***tert*-Butyl ((2*S*)-1-(((2*S*)-1-(((2*S*)-1-(((3*S*)-2-hydroxy-1-nitro-5-phenylpentan-3-yl)amino)-4-methyl-1-oxopentan-2-yl)amino)-3-methyl-1-oxobutan-2-yl)amino)-1-oxopropan-2-yl)carbamate 8.**


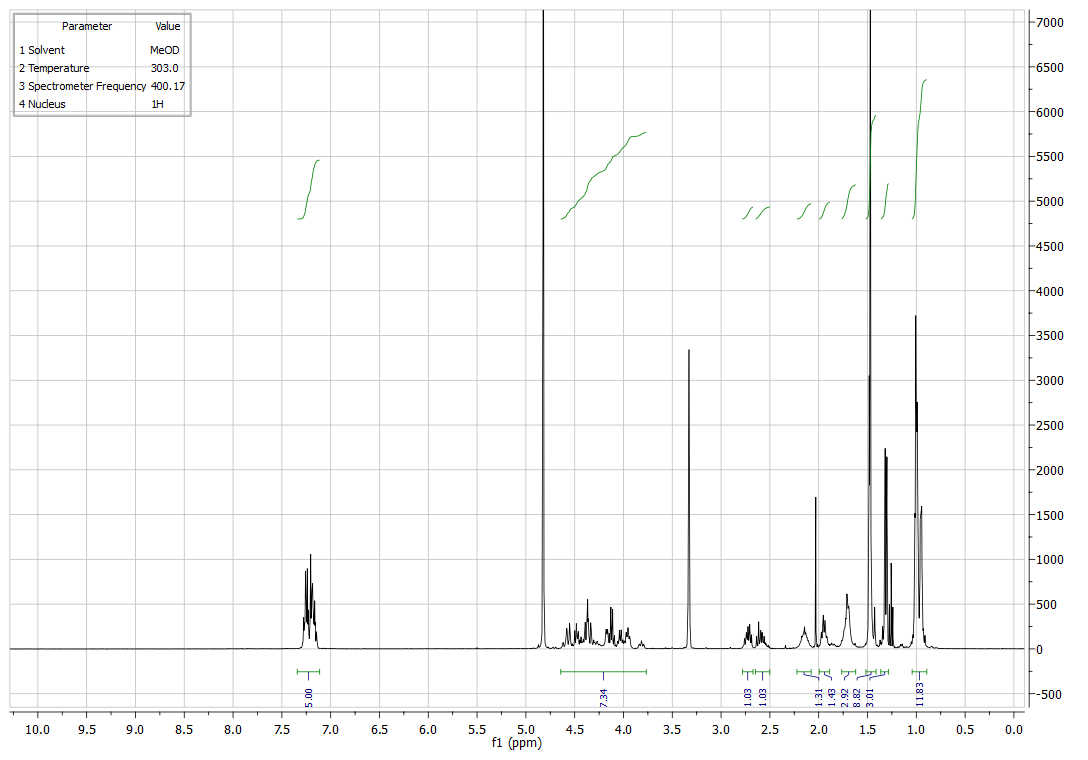


*
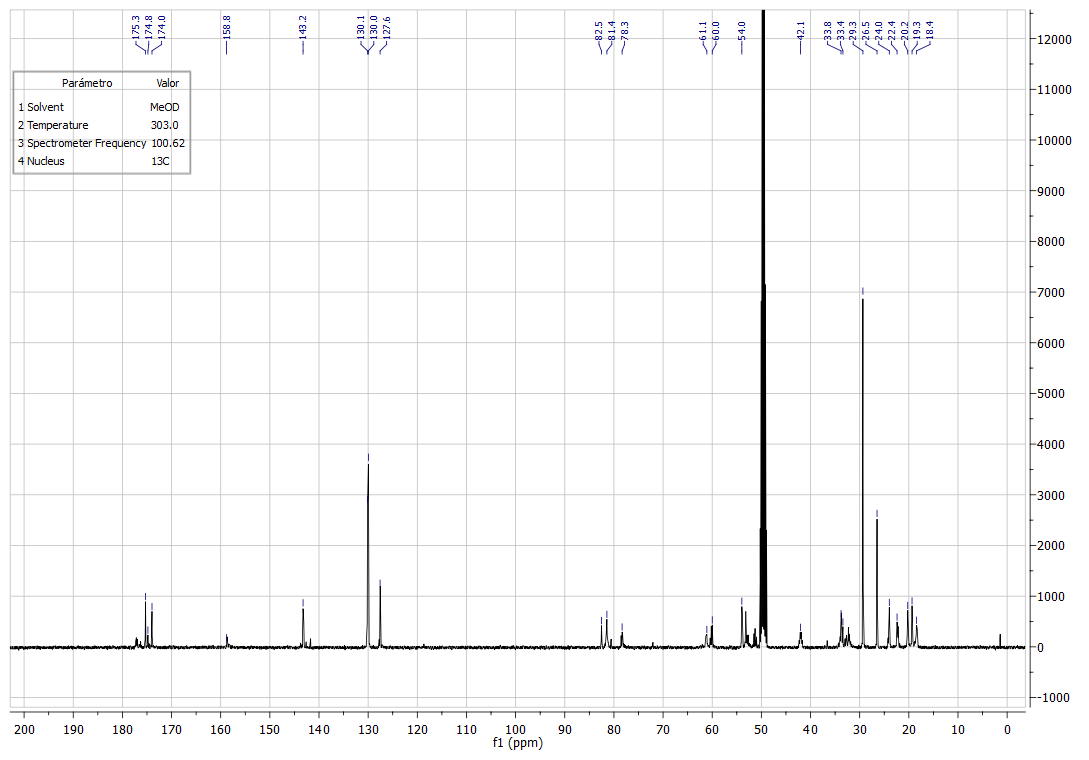
*

**FGA86**

*
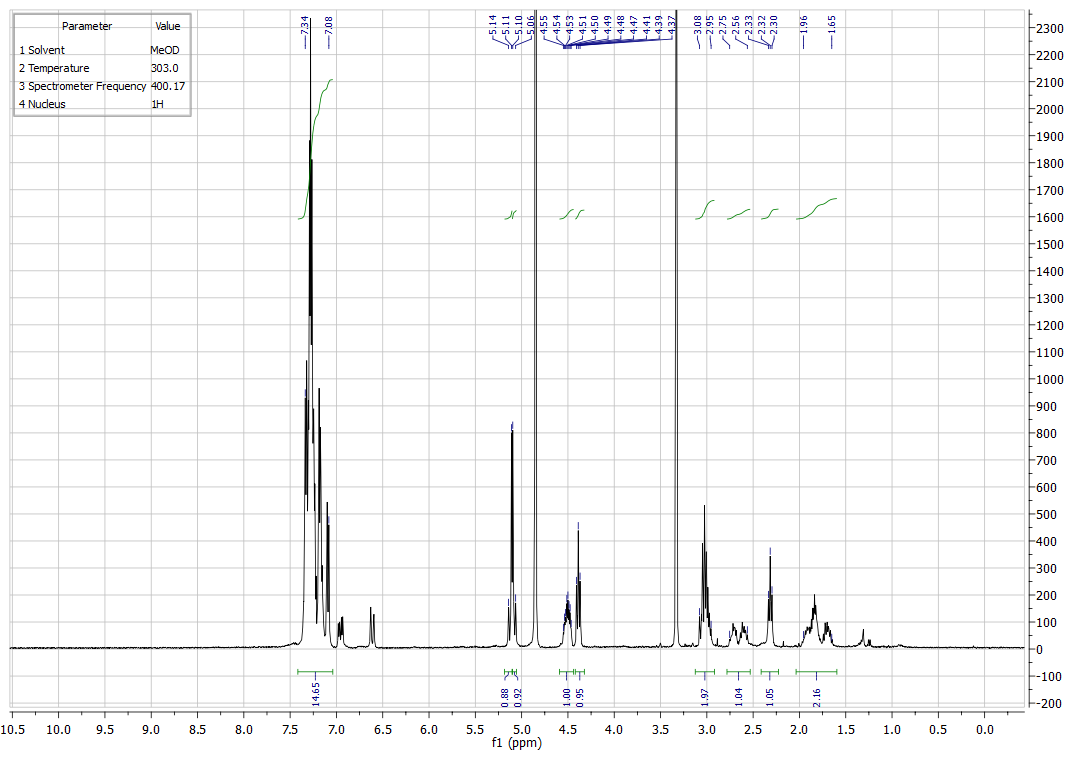
*

*
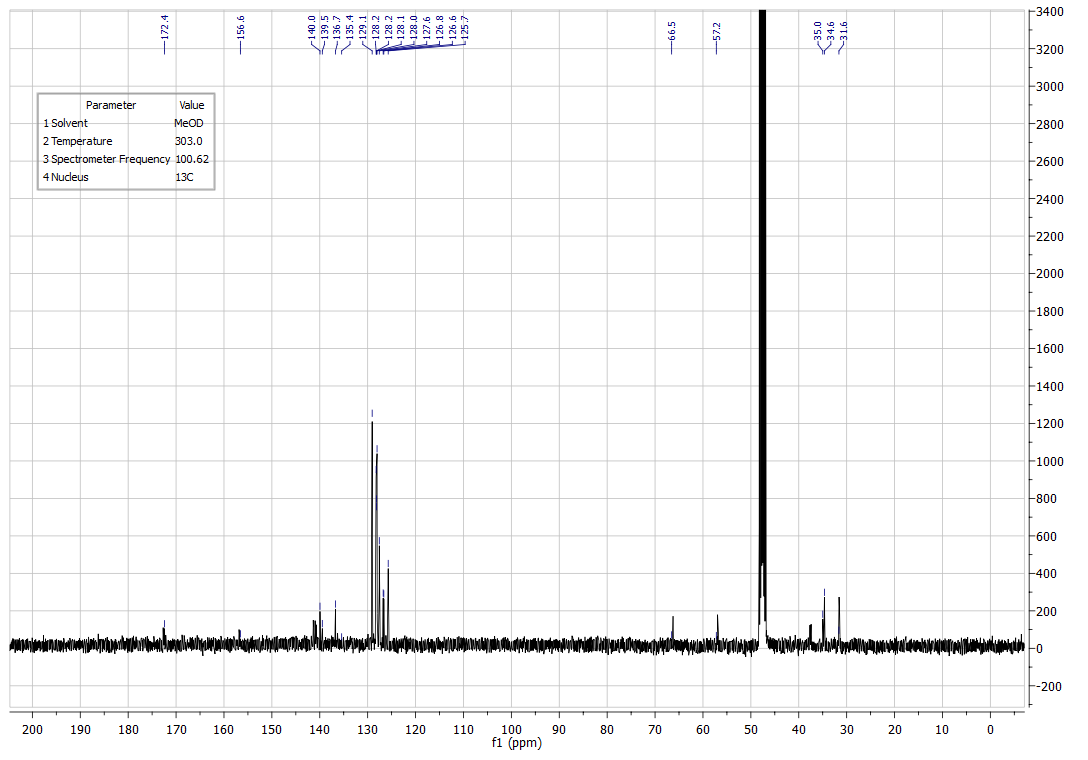
*

**FGA145**

*
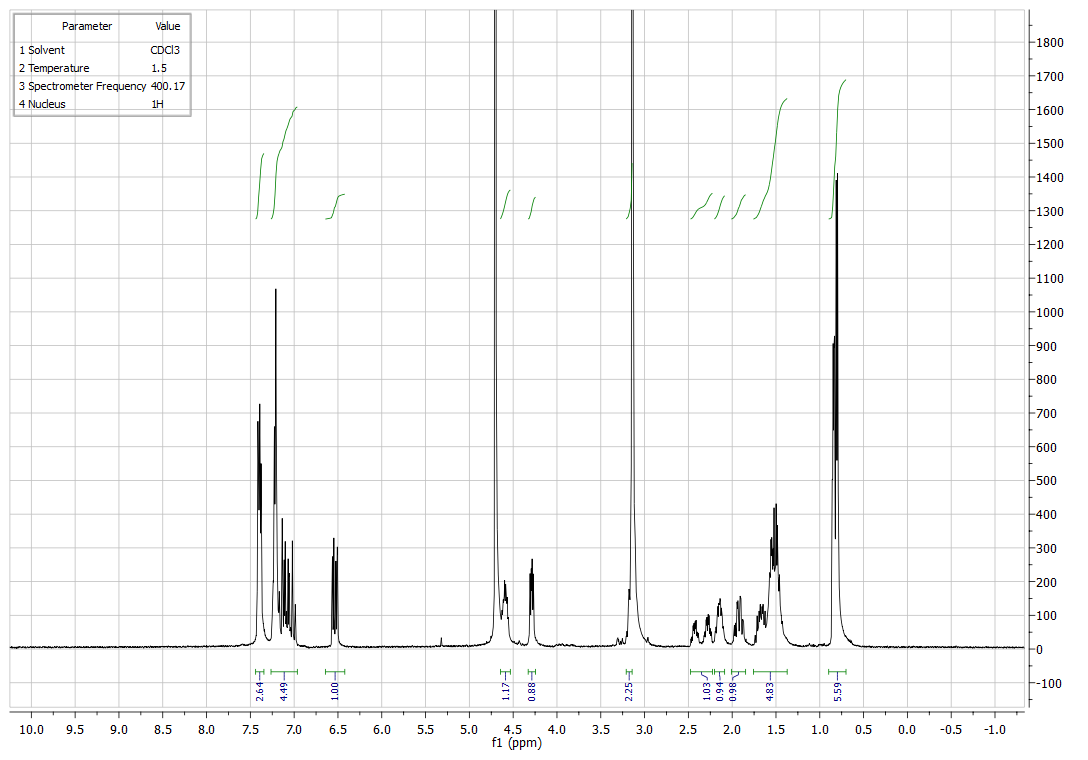
*

*
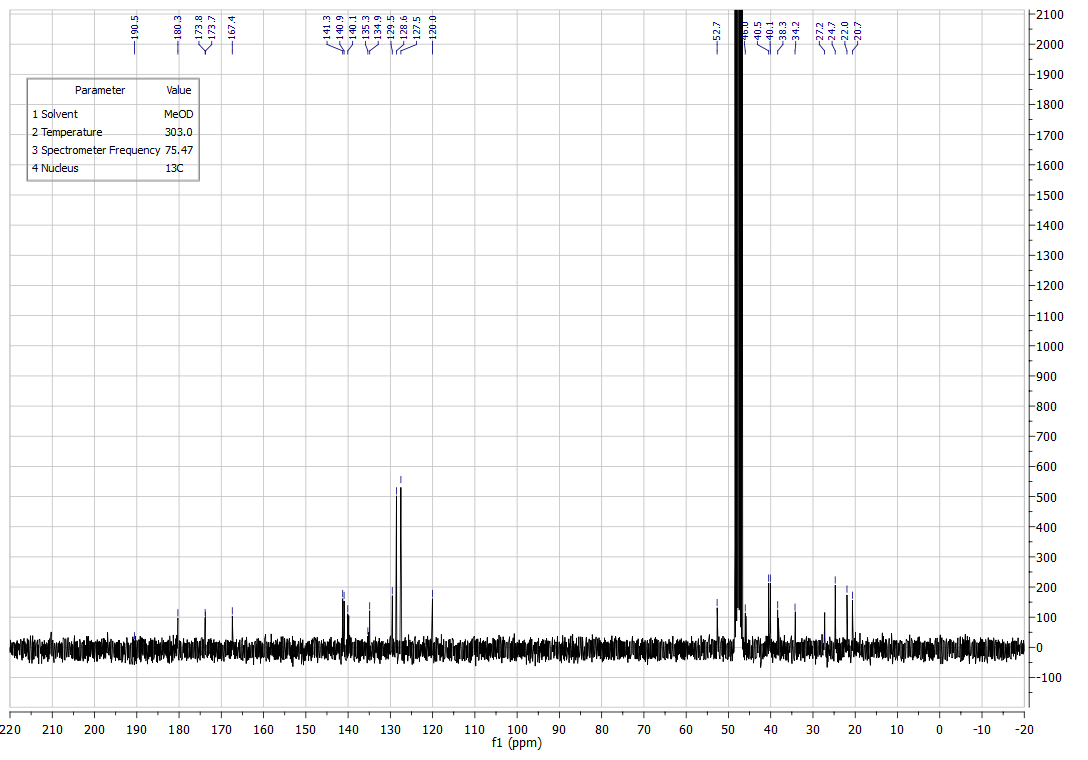
*

**FGA146**

*
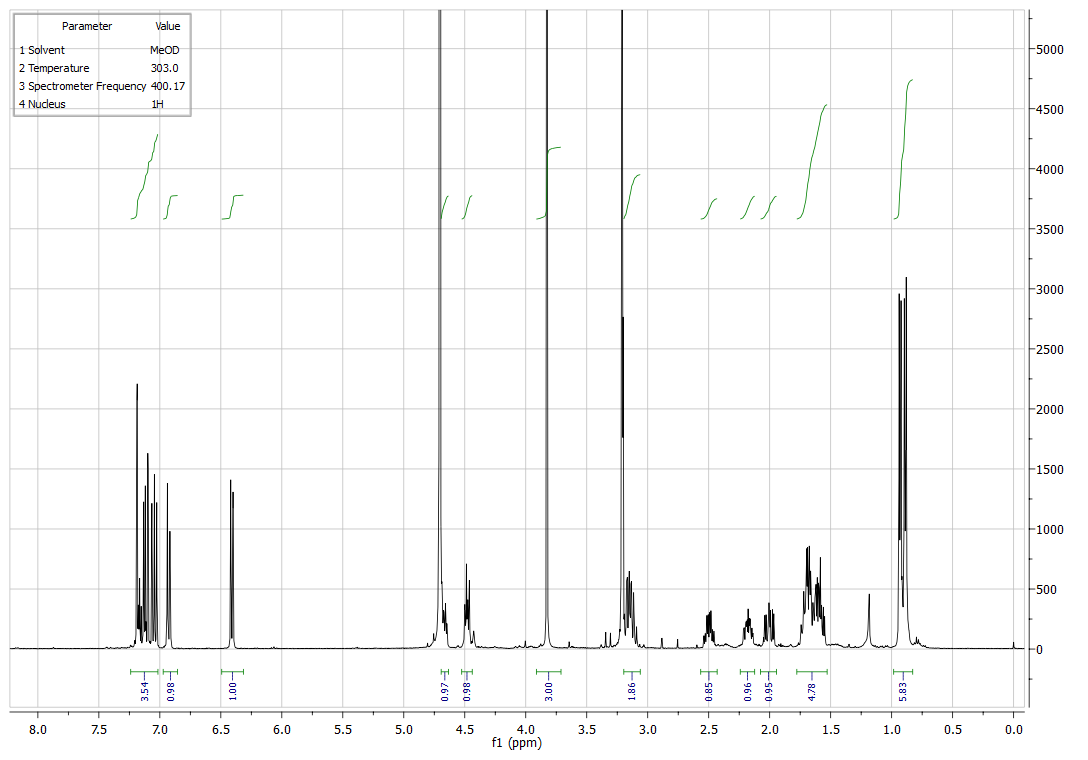
*

*
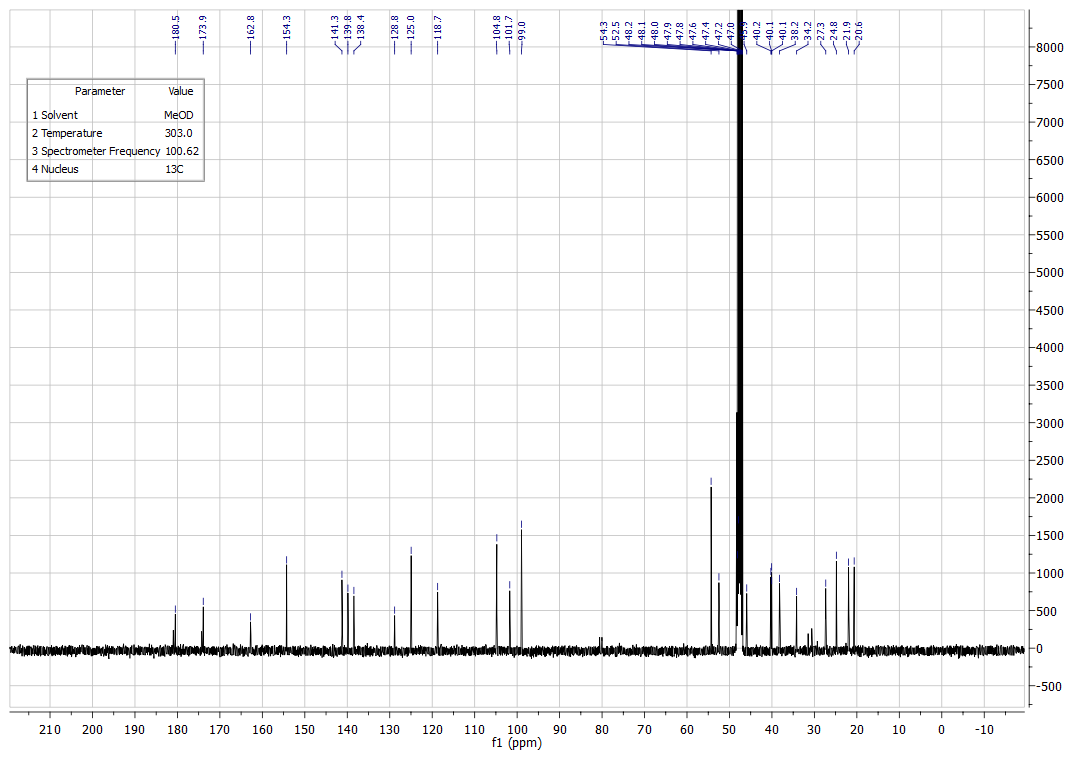
*

**FGA147**

*
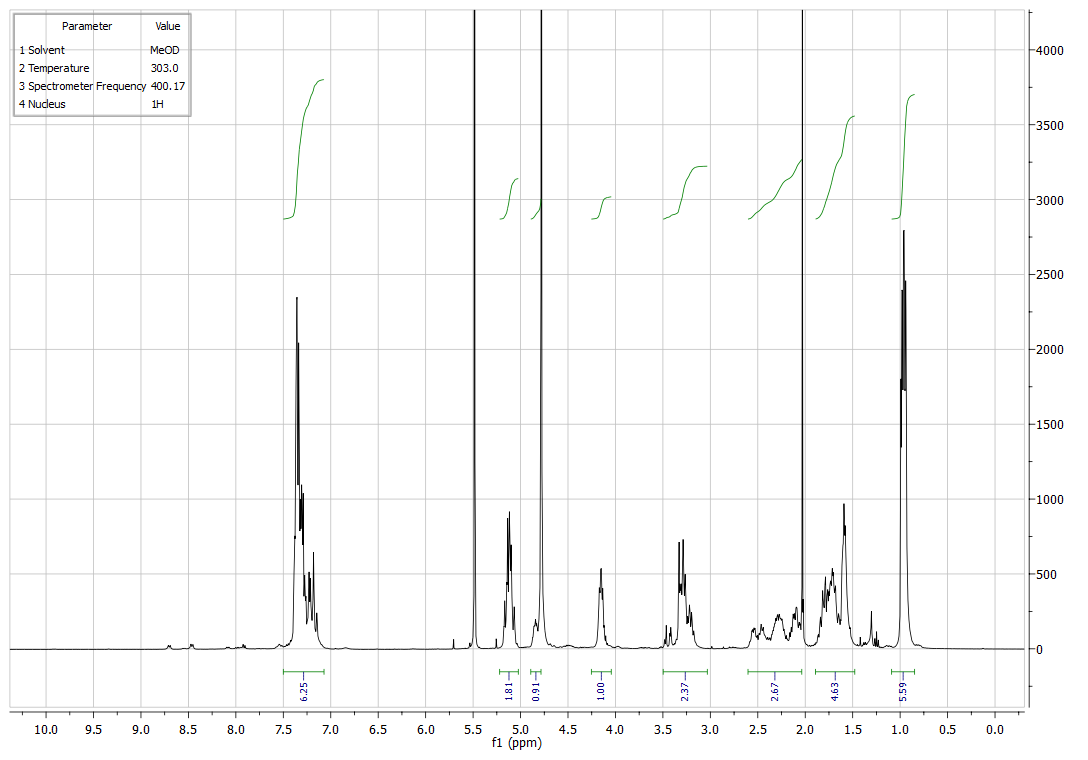
*

**
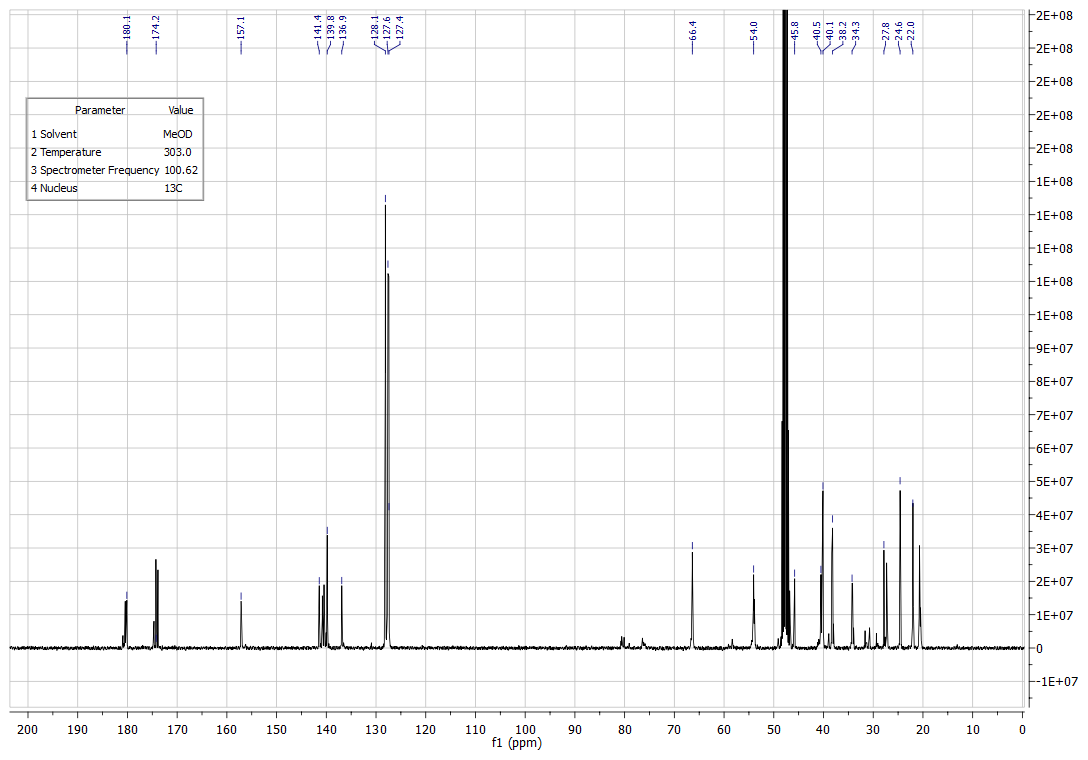
**

**
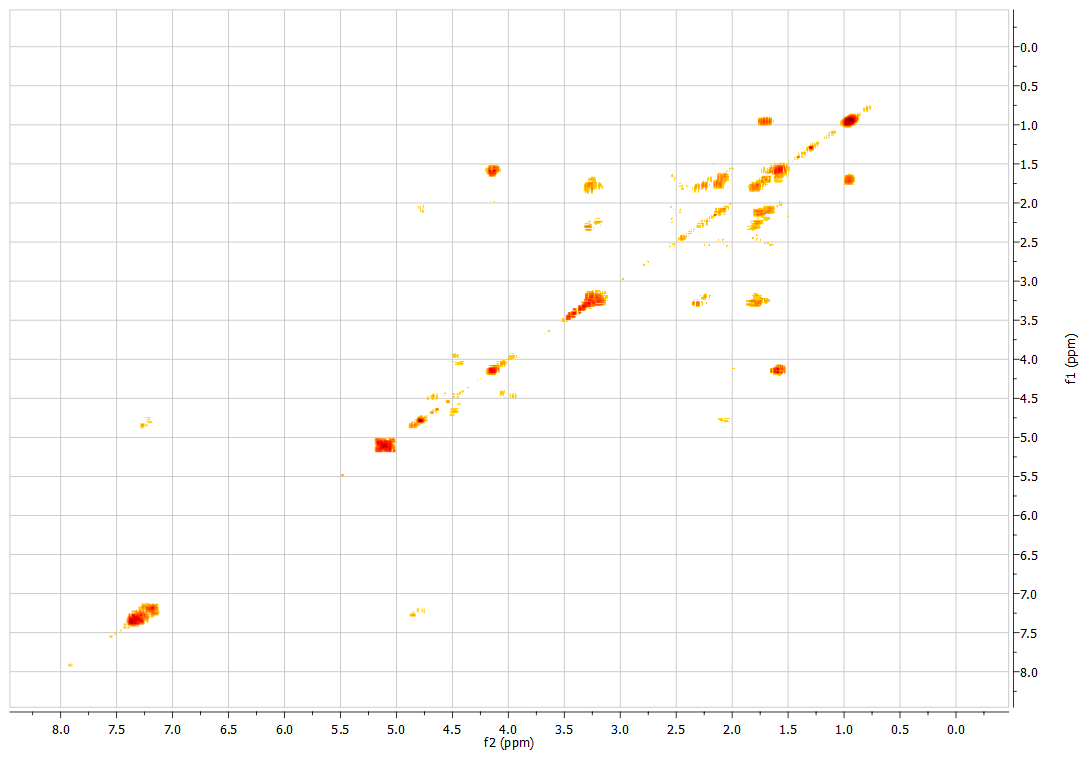
**

**
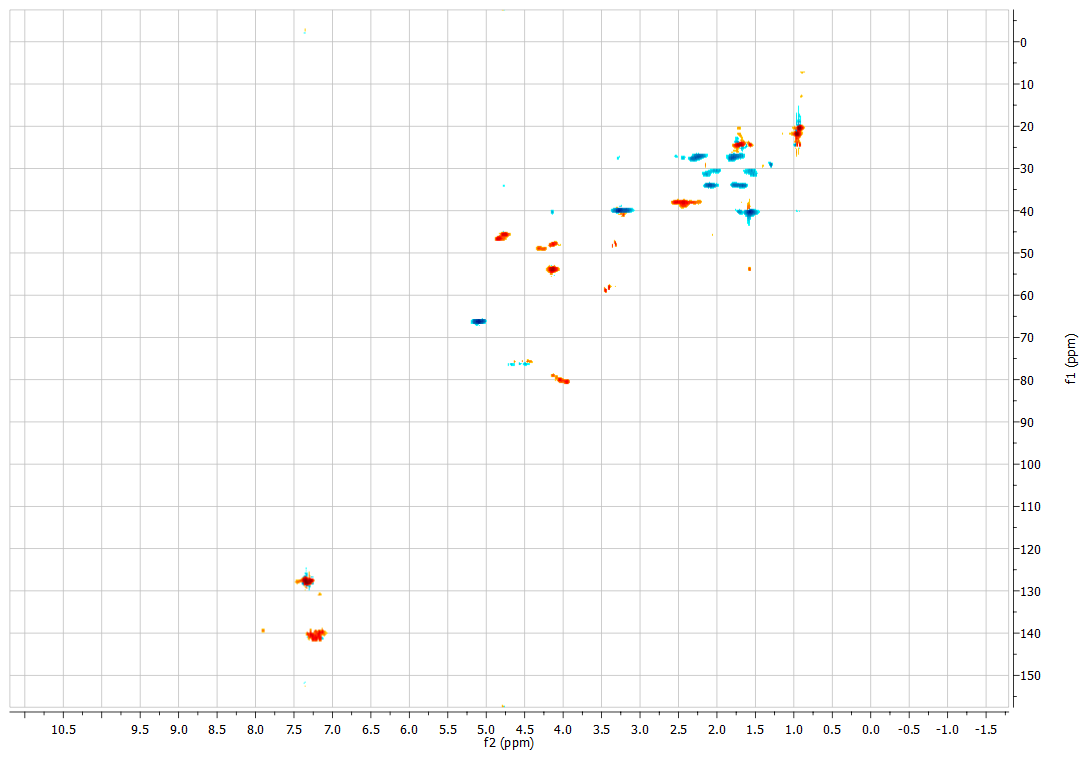
**

**FGA159**

**
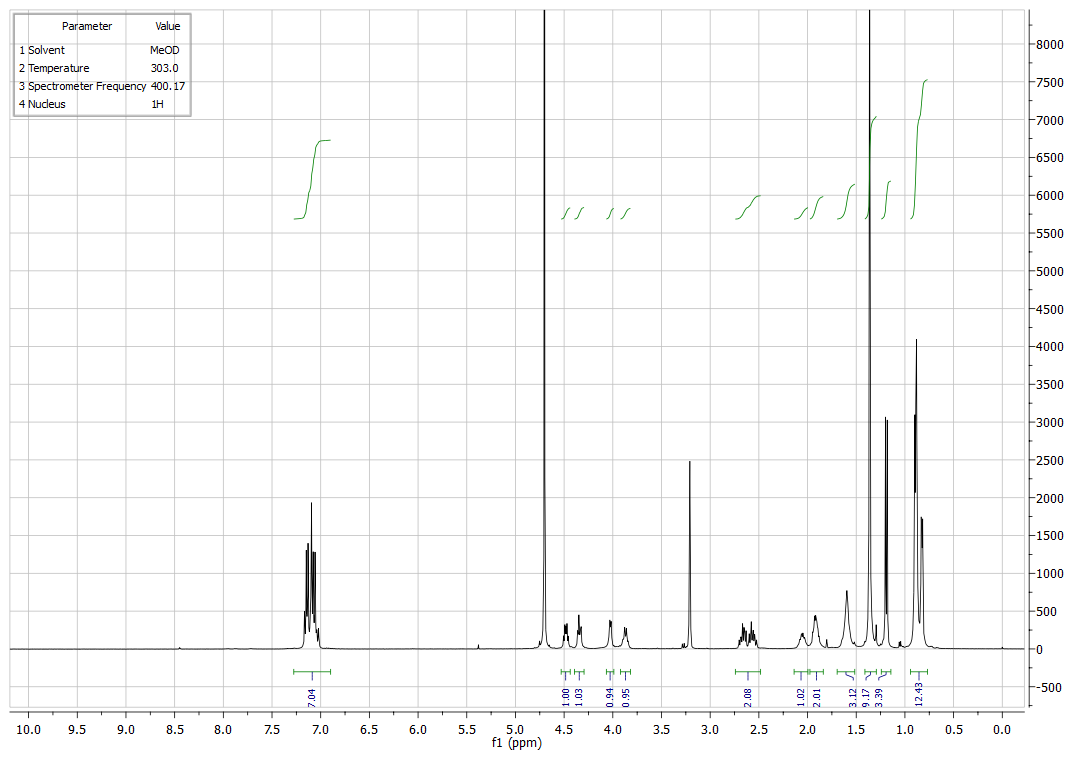
**

**
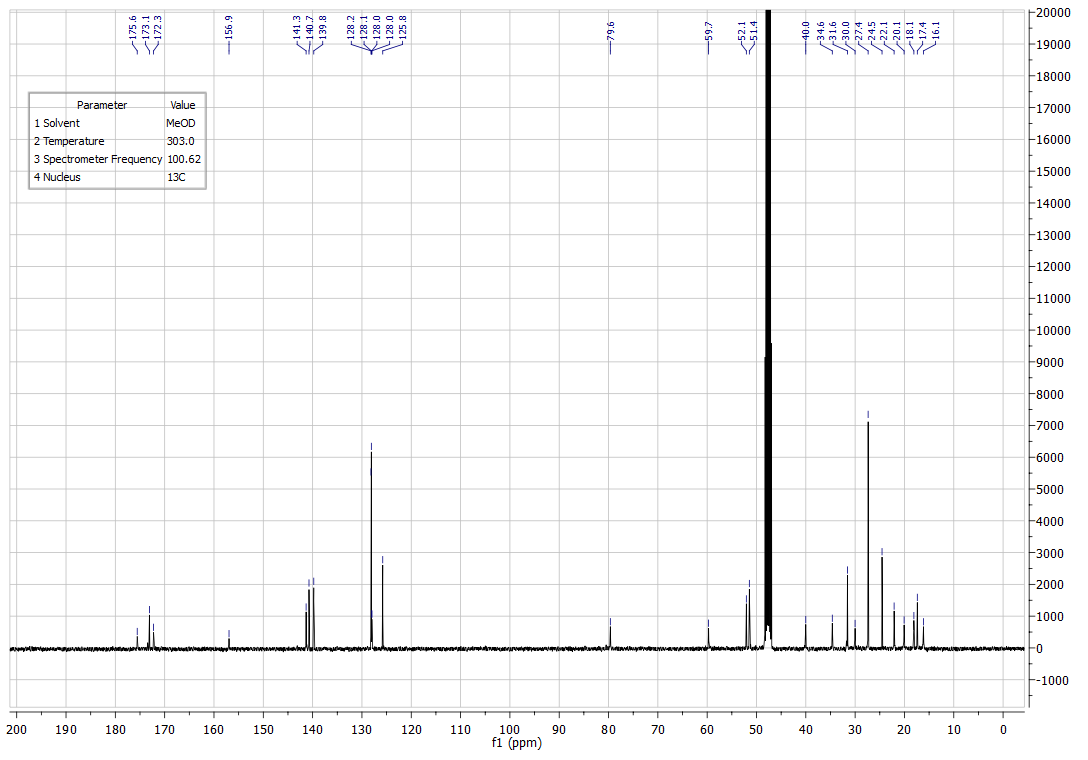
**

**
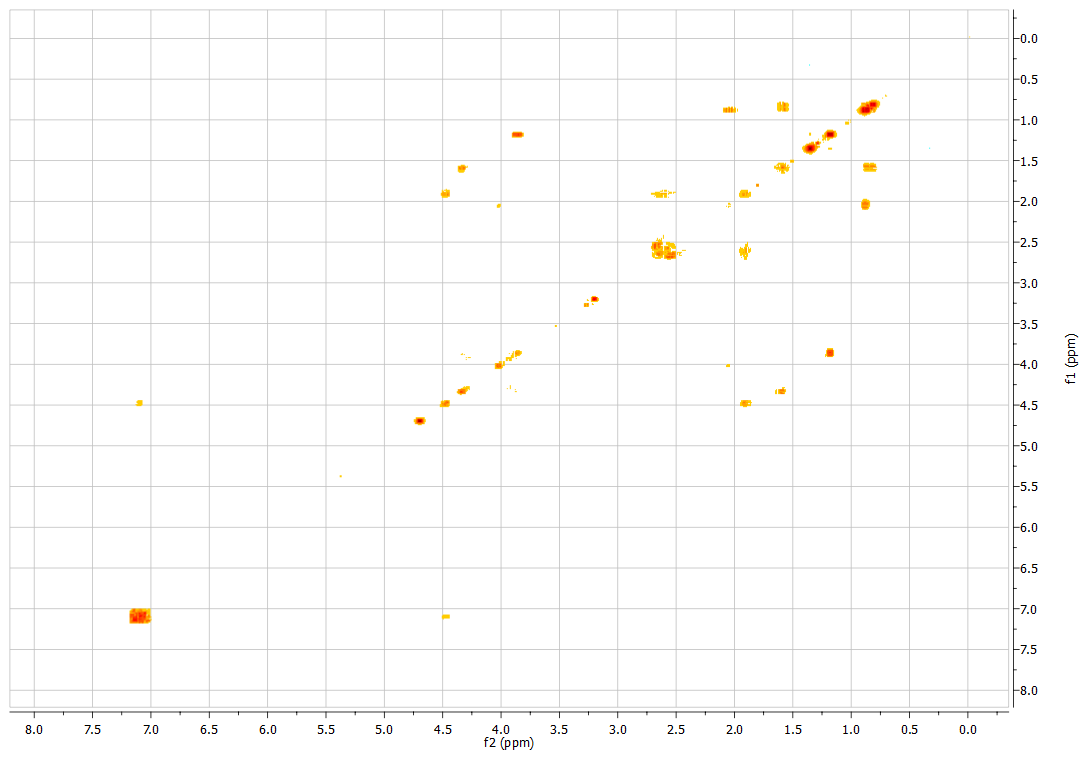
**

**
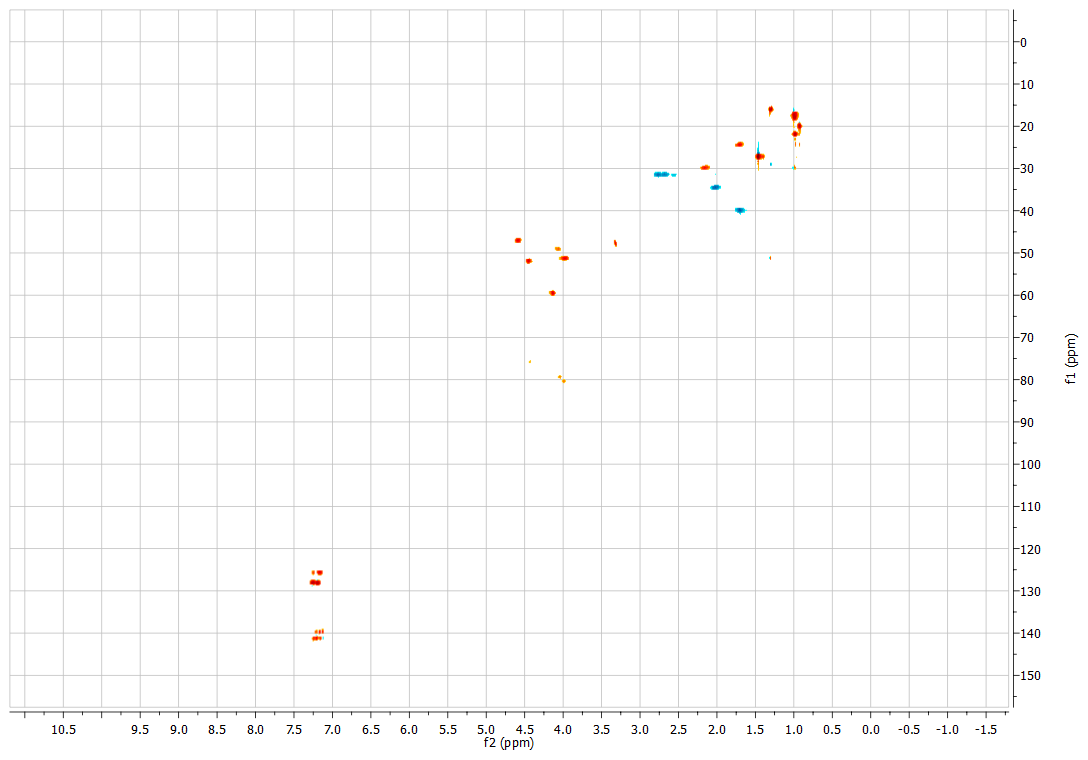
**

**FGA177**

*
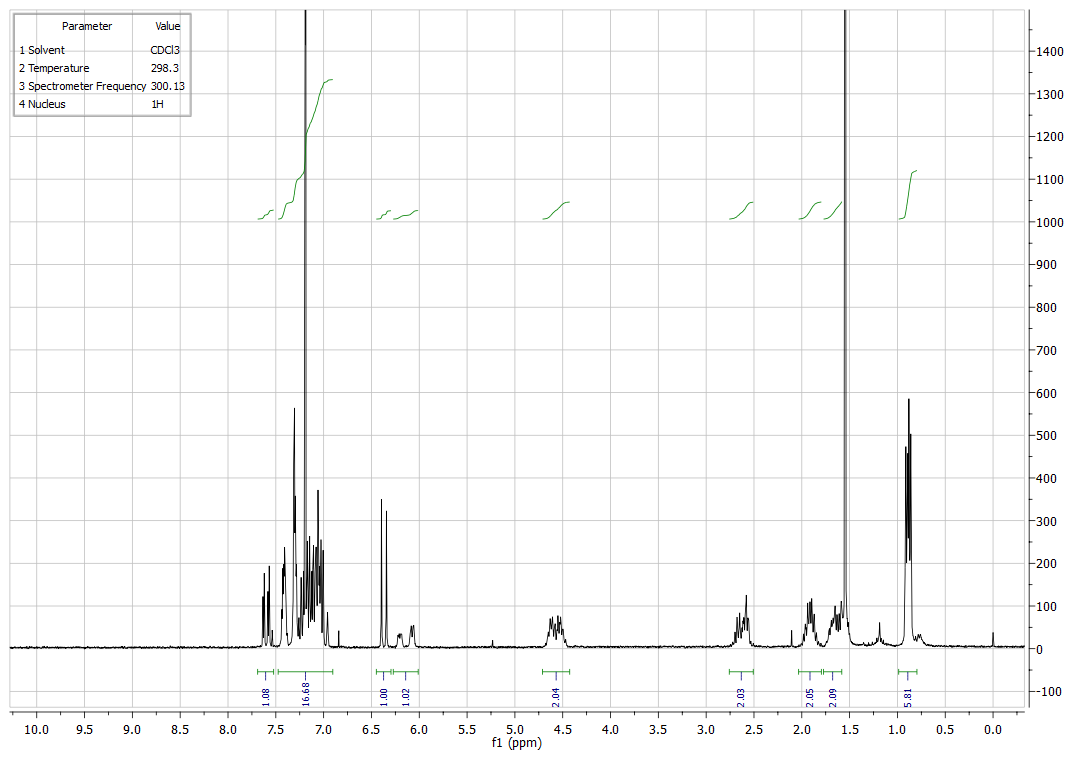
*

*
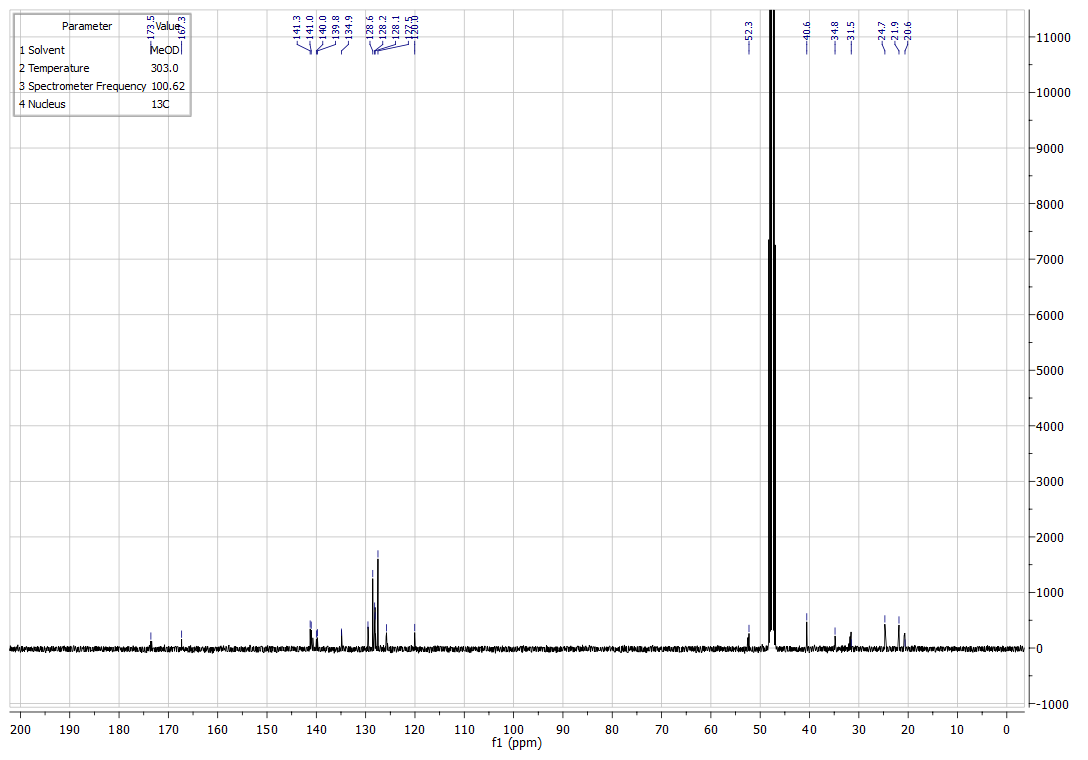
*

**FGA177**-COSY

*
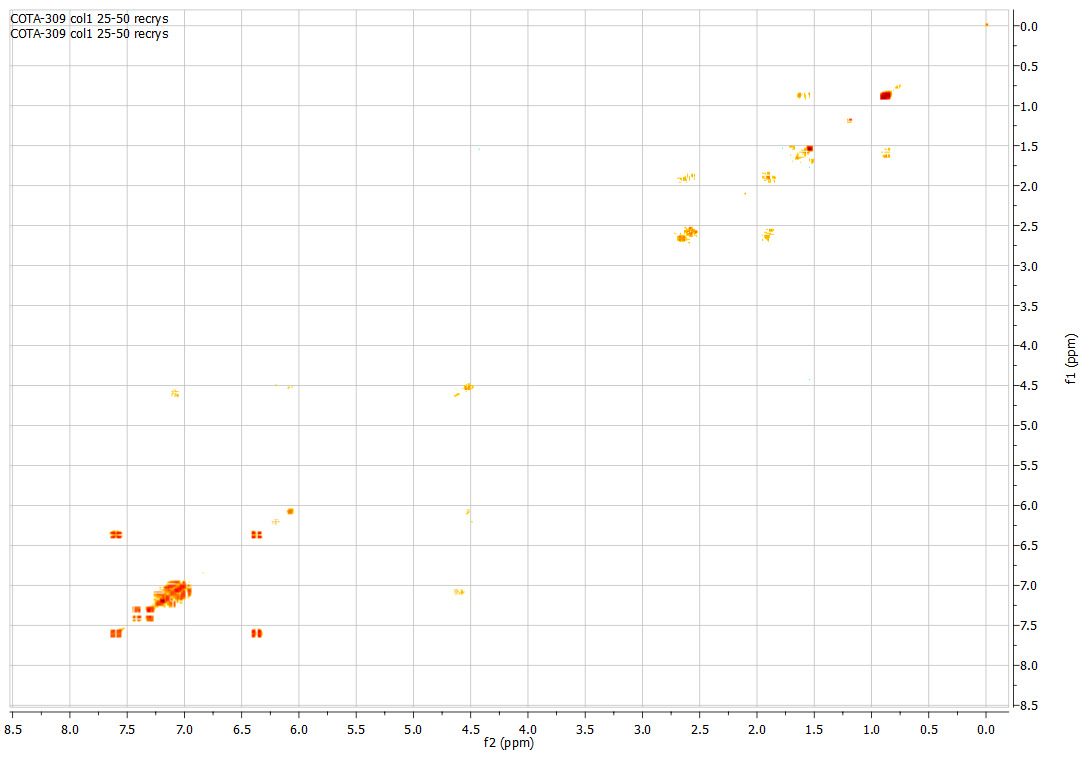
*

**FGA177**-HSQC

*
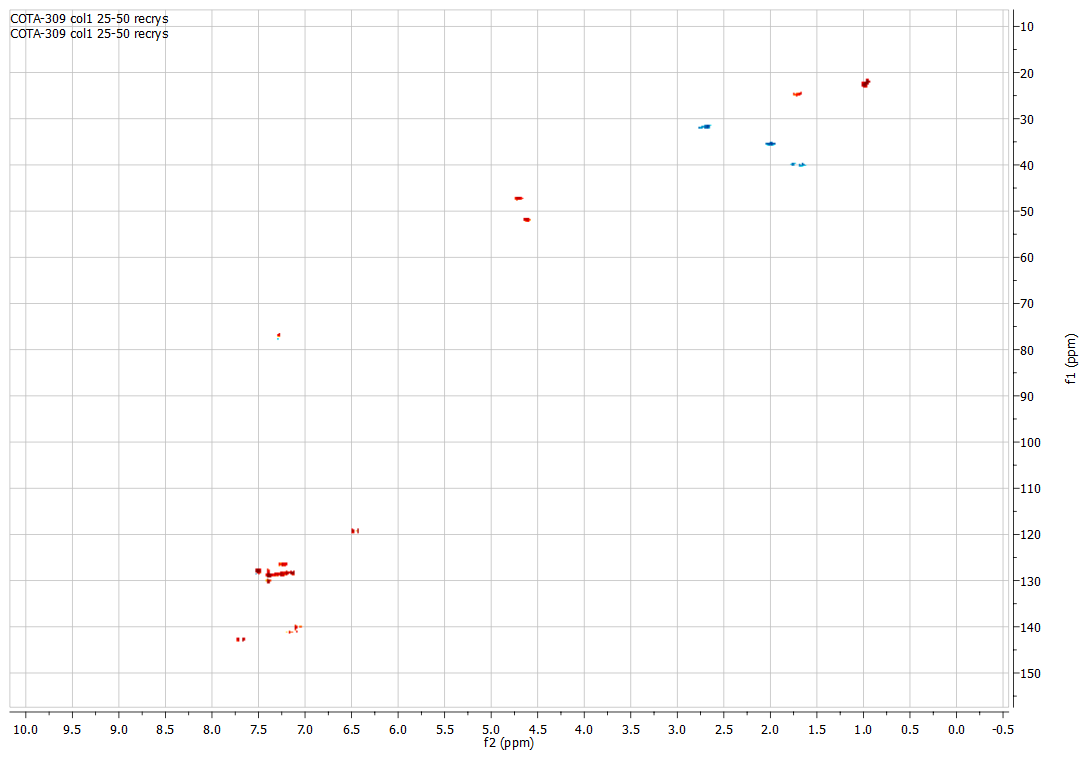
*
